# Targeting Stat3 with conditional knockout or PROTAC technology alleviates renal injury by Limiting pyroptosis

**DOI:** 10.1016/j.ebiom.2025.105739

**Published:** 2025-05-08

**Authors:** Ming-lu Ji, Jia-nan Wang, Ming-fei Wu, Chuan-hui Xu, Meng-meng Zhang, Wen-bao Chang, Xin-fei Mao, Chao Li, Ju-tao Yu, Dan-feng Zhang, Xiao-guo Suo, Shao-xi Diao, Nan-nan Ma, Ying Chen, Rui Hou, Hao Lu, Shuai-shuai Xie, Yu-hang Dong, Qi Zhu, Xin Chen, Tao Xu, Wei Shao, Juan Jin, Jia-gen Wen, Xiao-wu Dong, Wen-bin Wang, Jin-xin Che, Xiao-ming Meng

**Affiliations:** a Inflammation and Immune-Mediated Diseases Laboratory of Anhui Province, The Key Laboratory of Anti-inflammatory of Immune Medicines, Ministry of Education, Anhui Institute of Innovative Drugs, School of Pharmacy, Anhui Medical University, Hefei, 230032, China; b Hangzhou Institute of Innovative Medicine, Institute of Drug Discovery and Design, College of Pharmaceutical Sciences, Zhejiang University, Hangzhou, 310058, China; c Department of Nephrology, The Second Affiliated Hospital of Anhui Medical University, Hefei, 230002, China; d Anhui Provincial Corps Hospital of Chinese People's Armed Police Force, Hefei, 230032, China; e Anhui Provincial Chest Hospital, Hefei, 230022, China; f Department of General Surgery, The Second Affiliated Hospital of Anhui Medical University, Hefei, 230002, China; g Department of Vascular Surgery, The Second Affiliated Hospital of Anhui Medical University, Hefei, 230002, China

**Keywords:** Stat3, Histone modification, Pyroptosis, Renal injury, PROTAC

## Abstract

**Background:**

Acute kidney injury (AKI) is a critical clinical syndrome with high morbidity, mortality, and no effective treatment in clinical practice. The role of the Signal Transducer and Activator of Transcription 3 (Stat3) in AKI remains controversial, and its complex regulatory mechanisms must be further explored.

**Methods:**

We generated renal tubular epithelial cells Stat3 conditional knockout (cKO) mice and used them in cecal ligation and puncture (CLP) and ischaemia-reperfusion (I/R) induced AKI models. Additionally, proteolysis-targeting chimaera (PROTAC) compound E034 was designed and synthesised. We also utilised human kidney tissues, mouse renal tubular epithelial cells (mTECs) and HK-2 cells for further studies, including immunohistochemistry, Western blot analysis, Real-time PCR, chromatin immunoprecipitation (ChIP) and RNA sequencing, scanning electron microscopy (SEM) and Co-Immunoprecipitation (Co-IP) assay.

**Findings:**

An upregulation of total Stat3 protein was observed in AKI mouse models, which correlated with patient biopsy results. This increase may be attributed to histone H3K27 acetylation. Stat3 knockout in renal tubular epithelial cells significantly reduced AKI injury and inflammation in mice. Mechanistically, Stat3 induces the transcription of tripartite motif-containing protein 21 (Trim21), triggering a cascade that activates gasdermin D (Gsdmd), resulting in pyroptosis. Administration of E034, which selectively targets Stat3 for ubiquitination and degradation, significantly alleviated renal injury in a low-dose, single-dose regimen.

**Interpretation:**

In the context of renal injury, PROTAC emerges as a promising modality by explicitly targeting the Stat3/Trim21/Gsdmd axis, which our study has identified as a potential therapeutic target, potentially endowing clinically significant therapeutic strategies.

**Funding:**

This work was supported by the National Key R&D Program (2022YFC2502503), the 10.13039/501100001809National Natural Science Foundation of China (No. 82270738), the 10.13039/501100001809National Natural Science Foundation of China (No. 82400806) and the Graduate Research and Practice Innovation Project of Anhui Medical University (YJS20230059).

**Research in context**

**Evidence before this study**

AKI is a severe clinical syndrome characterised by high morbidity and mortality, with no effective treatments currently available. Phosphorylation of Stat3 up-regulates the transcriptional upsurge of key AKI-related molecules, playing a crucial role in AKI development. However, besides phosphorylated Stat3, other post-translational modifications of Stat3 may also influence its transcription factor function. The upregulation of total Stat3 protein has been suggested as a pivotal factor in AKI, yet exploration from this perspective remains scarce. Current small-molecule drugs targeting Stat3 phosphorylation also have non-negligible drawbacks.

**Added value of this study**

Using Stat3 conditional knockout model animals, our study elucidated the pro-inflammatory and pyroptosis-inducing effects of Stat3 in AKI and the underlying mechanism. We introduced the PROTAC compound E034 as an innovative approach. E034 selectively degrades Stat3 via ubiquitination, an innovative mechanism in AKI treatment. In *in-vivo* experiments, a single, low-dose administration of E034 effectively reduces renal damage. Pre-clinical studies further demonstrate its potential in alleviating kidney injury and pyroptosis, offering a strategy for targeting Stat3 and highlighting the superiority of PROTAC-based therapy over traditional methods.

**Implications of all the available evidence**

This study significantly deepens our understanding of AKI pathophysiology, especially the Stat3 pathway and its regulation. Clinically, it provides a promising therapeutic avenue for patients with AKI, filling a critical void in existing treatments. The discovery of E034 may inspire another treatment protocol, improving patient prognosis and reducing the high morbidity and mortality associated with AKI. Future research could focus on the long-term effects of E034, its safety in more significant patient populations, and its combination with other therapies, which may reshape the clinical management strategies for AKI.

## Introduction

Acute kidney injury (AKI) is a critical clinical syndrome characterised by abrupt deterioration of renal function. It is associated with significant morbidity, mortality, and poor prognosis, exerting a pervasive impact on nearly every body system.^1^^,^^2^ Its multifactorial aetiology involves ischaemia-reperfusion (I/R) injury,^3^ sepsis,^4^^,^^5^ exposure to nephrotoxic agents,^6^ and use of contrast media,^7^ among other factors. Hallmark pathological manifestations of AKI include renal tubular epithelial apoptosis, inflammatory cell infiltration, and mitochondrial oxidative stress.^8^, ^9^, ^10^ Presently, no effective AKI prevention or cure exists in clinical practice.

Signal transducer and activator of transcription protein 3 (Stat3) orchestrate many intracellular signalling cascades, exerting a pivotal influence on cellular processes such as proliferation, differentiation, and apoptosis.^11^^,^^12^ Several studies have established that inhibiting the phosphorylation-dependent activation of Stat3 can significantly ameliorate both the acute and chronic phases of renal injury.^13^, ^14^, ^15^ However, recent evidence indicates other post-translational modifications are also crucial for its transcriptional activity, and conflicting views exist on the role of Stat3 in AKI.^16^, ^17^, ^18^ This gap has sparked profound interest and highlighted the need for further research.

Interestingly, our investigation revealed that in AKI mouse models and human renal biopsy tissues, there is not only an upregulation of the phosphorylation state of Stat3 but also a significant induction of its total protein expression, which is predominantly localised in renal tubular cells. To delve deeper into the impact of total Stat3 protein changes on AKI, we generated a conditional knockout (cKO) mouse model of renal tubular epithelial cells lacking Stat3 rather than commonly used methods inhibiting Stat3 phosphorylation activation. Furthermore, we engineered proteolysis-targeting chimaera (PROTAC) designed to degrade Stat3. These findings advance our understanding of the role of Stat3 in AKI and may pave the way for targeted Stat3 therapies for clinical AKI treatment.

## Methods

### Experimental design

The objectives of this study were to examine the role of Stat3 in the pathogenesis of AKI and to elucidate the underlying mechanisms. To achieve these objectives, we designed a study utilising human kidney tissues, two mouse models of AKI, and *in vitro* models of renal tubular cell injury to ascertain the role of Stat3. Patients with AKI and acute tubular necrosis, confirmed by renal biopsy, were enrolled at the Second Affiliated Hospital of Anhui Medical University. Nontumor kidney tissue from patients who underwent nephrectomy due to renal cell carcinoma was used as a standard control for analysing Stat3 expression in kidney tissues. We endeavoured to use littermates for the animal studies, which aids in randomisation. The sample size for the animal studies was based on our experience with similar studies. The sample size (n) for each experimental group is indicated in the figure legends, with approximately six mice per group. Within the littermate groups, AKI was induced by exposure to cecal ligation and puncture (CLP) and I/R in mice with or without Stat3 expression. PROTAC, designated E034, was designed and synthesised to intervene in the AKI model. For cell studies, a minimum of three experimental replicates were conducted in Mouse tubular epithelial cells (mTECs) and HK-2 with knockdown or overexpression of Stat3. The number of replicates is presented in the figure legends. Two independent, blinded pathologists performed all immunohistochemical quantifications. Statistical tests were selected based on the variables' nature, the data distribution assumptions, and the effect size.

### Reagent validation

All reagents used in this study, including antibodies and cell lines, were commercially sourced. Antibody information, including catalogue numbers, vendors and Research Resource Identifier (RRID), is provided in Table S1. The full Western blot membranes for antibody specificity detection are shown in Figures S16–S23. The detailed documentation of reagent validation is available in Table S2. Source Information for Model Organisms is shown in Table S3. Cell lines were authenticated through STR profiling, and the STR results are included in Table S4 and the files ‘mTECs STR Identification’ and ‘HK-2 STR Identification’. The sh RNA and si RNA sequences used are shown in Table S5. The primer sequences used are shown in Table S6.

### Ethics statement

All animal experiments conducted at Anhui Medical University, China, were approved by the Animal Experimentation Ethics Committee and performed following the Guide for the Care and Use of Laboratory Animals (Approval number: LLSC20232176). All experiments with human samples were approved by the Biomedical Ethics Committee of Anhui Medical University (Approval number: 83243416).

### Human specimens

Stat3 expression was evaluated via immunohistochemistry in tissue samples from 4 patients with AKI and 4 paracancerous kidney tissue biopsies from renal carcinoma patients. Para-carcinoma samples were derived from patients undergoing renal cell carcinoma resection, with histopathological confirmation of non-tumorous tissues located ≥2 cm from the tumour margin to exclude malignant infiltration. In contrast, samples were collected from patients meeting KDIGO criteria for AKI, supported by clinical and laboratory evidence of acute renal dysfunction. Sex (male/female), self-reported by participants and recorded in medical records, was collected as a demographic variable. No sex-specific inclusion/exclusion criteria were applied; the cohort comprised both male and female individuals. Demographic data included: for the para-carcinoma group, 2 males and 2 females (range 45–67 years, all East Asian); and for the AKI group, 3 males and 1 female (range 44–74 years, all East Asian). All samples were retrospectively collected from the Second Affiliated Hospital of Anhui Medical University between March 2024 and March 2025, anonymised, and approved by institutional ethics committees. For detailed information, please refer to Table S7. The study adhered to the Declaration of Helsinki, with written informed consent obtained from all participants, and focused on Stat3 expression in renal injury contexts.

### Animal studies and generation of kidney-specific Stat3 knockout mice

6–8-week-old male C57BL/6 mice (approximately 20–22 g in weight) were provided by the Experimental Animal Centre of Anhui Medical University. In most studies on AKI in mice, male mice have been the preferred subject because of the observed delay in the onset of renal impairment in female mice in the murine model of AKI.^19^ Only male mice were included to ensure consistency, excluding females to control for sex-related biological differences in disease progression. Sex was confirmed by physical examination upon receipt and recorded in study logs.

Methods for CLP- and I/R-induced AKI were derived from previous reports.^20^, ^21^, ^22^ 1) CLP-Induced AKI: C57 mice were anaesthetised via intraperitoneal injection of sodium pentobarbital (50 mg/kg). The abdomen was sterilised, and the skin and peritoneum were incised to expose the caecum. The caecum was doubly ligated with a 4/0 silk suture at a point one-third away from its distal end. An 18G needle was then used to create 2–3 punctures in the ligated segment, and the caecum was gently squeezed to ensure spillage of intestinal contents. After repositioning the caecum, the abdominal wall was sutured layer by layer. Postoperatively, mice received subcutaneous injections of physiological saline (10 mL/kg) for fluid resuscitation. Early success criteria for modelling: At 24 h post-surgery, animals exhibited typical septic symptoms, including hypothermia (<35 °C), lethargy, reduced activity, piloerection, and loss of appetite. Additionally, serum creatinine (CRE) and blood urea nitrogen (BUN) levels were ≥2-fold higher than preoperative baselines or histological examination of kidney tissues revealed tubular dilation, brush border loss, and luminal casts, confirming successful AKI induction. 2) I/R-Induced AKI: C57 mice were anaesthetised, and bilateral dorsal lumbar incisions were made to expose the renal arteries. Non-traumatic vascular clamps were applied to occlude the renal arteries for 40 min, followed by reperfusion upon clamp removal. The abdominal wall was sutured layer by layer, and postoperative subcutaneous fluid resuscitation was performed. Sham-operated mice underwent the same procedure but with renal artery exposure only (without clamping). Early success criteria for modelling: At 24 h post-reperfusion, animals displayed oliguria or anuria, and serum CRE and BUN levels were ≥1.5-fold higher than those in sham-operated controls, indicating significant renal dysfunction. Histological analysis of kidney tissues confirmed AKI with findings of swelling and necrosis of tubular epithelial cells and numerous casts within the renal tubules.

For adeno-associated virus serotype 9 (AAV9)-mediated Stat3 knockdown or overexpression in mice, adeno-associated viruses were obtained from Hanbio (Shanghai, China). The Stat3 silencing sequence has been reported previously.^23^ At least six mice were included in each group.

Since global Stat3 knockout is embryonically lethal, we generated a Stat3 cKO mouse line (C57BL/6 background) in which Stat3 is specifically depleted in distal convoluted tubules and collecting duct. Stat3^flox/flox^ (FF) mouse lines were constructed by Shanghai Model Organisms Centre, Inc. (Shanghai, China). Stat3 cKO mice were generated by mating Stat3^flox/flox^ mice with mice expressing Cre recombinase under the control of a kidney-specific promoter (cadherin-16). All mice were genotyped by PCR before and after the experiments.

### Cell lines and culture conditions

For cell culture studies, the human HK-2 cell line (purchased from Procell) underwent recent STR profiling by the vendor, with accompanying certification provided (see uploaded STR reports)—the murine renal tubular epithelial cell line (mTECs), immortalised and gifted by the HY.Lan Lab (The Chinese University of Hong Kong) was tested for STR identity and mycoplasma contamination by Shanghai Biowing Applied Biotechnology Co. Ltd. Both cell lines showed confirmed genetic authenticity and negative mycoplasma results, with detailed reports from the testing companies included as Supplementary material. mTECs and HK-2 were cultured in HyClone Dulbecco's modified Eagle's medium (DMEM)/F12 containing 5% foetal bovine serum (FBS) under 5% CO_2_ condition. The silencing and overexpression experiments of Stat3 and Trim21 were conducted by transfecting cells with specific sh RNA, si RNA and overexpression plasmids, respectively (The corresponding sequences are shown in Table S5). After overnight starvation in DMEM/F12 medium with 0.5% FBS, cells were treated with LPS (20 μg/mL) for 24 h or subjected to an anoxic condition featuring 94% nitrogen, 5% CO_2_, and 1% O_2_ for 12 h, followed by exposure to 5% CO_2_ condition for 3 h to establish an anoxic reoxygenation (H/R) model.

### Renal histology and immunohistochemistry

Paraffin-embedded mouse kidney sections of 4 μm in thickness were prepared using a standard procedure, including fixation, dehydration, waxing, and embedding. Periodic acid-schiff (PAS) and haematoxylin and eosin (H&E) staining were performed, followed by light microscopy histological examinations. The extent of tubular damage, including tubular dilation, atrophy, and cast formation, was assessed by three experienced renal pathologists blinded to the experimental groups. The scoring criteria were as follows: 0 = normal; 1 = 10%; 2 = 10–25%; 3 = 26–50%; 4 = 51–75%; 5 = 76–95%; 6 = more than 96%.

Immunohistochemical staining was performed according to the manufacturer's instructions. Antibodies specific for Stat3, Trim21, and F4/80 were incubated overnight at 4 °C, followed by a 1-h incubation with secondary antibodies at 22–26 °C. After staining with diaminobenzidine (DAB) and counterstaining with haematoxylin, the immunoreactivity of the sections was visualised under a microscope (Olympus IX83, Japan).

### Western blot analysis

Protein lysates from the kidney tissues and cultured cells were prepared according to standard protocols. Western blot analysis was performed as previously described.^21^^,^^24^, ^25^, ^26^ The primary and secondary antibodies used in this study have been described previously. Signals were detected using the Odyssey infrared imaging system. The results were quantitatively analysed using the ImageJ software.

### RNA isolation and quantitative real-time PCR

Total RNA was extracted from cells or tissues using AG RNAex Pro Reagent, following the manufacturer's instructions. Real-time PCR was performed using 2× SYBR Green Pro Taq HS Premix∗ on Opticon 2 real-time PCR detection system (Bio-Rad) using CFX96 real-time RT-PCR detection system, as stated in our team's published article.^27^ The primers used in this study are listed in Supplementary Table S6.

### Immunofluorescence

Embedded kidney tissue sections (4-μm thick) were dewaxed and returned to room temperature after antigen retrieval using 1 × EDTA working solution diluted from 50 × EDTA repair solution (pH = 8.0). Alternatively, the cells were attached to coverslips and fixed with paraformaldehyde. The samples were incubated overnight with the desired primary antibodies in a 10% bovine serum albumin (BSA) solution. After washing with phosphate-buffered saline (PBS), the samples were incubated for 2 h at room temperature with both goat anti-rabbit IgG-rhodamine and goat anti-mouse IgG-rhodamine secondary antibodies (Bioss Biotechnology, Beijing, China). The tissue sections and cells were counterstained with DAPI and visualised using a fluorescence microscope (Leica, Bensheim, Germany).

### Cell viability assay

Cell viability was determined using the MTT assay based on the purple formazan product formation principle by mitochondrial dehydrogenase in viable cells. Briefly, mTECs were seeded in 96-well plates and cultured with different known concentrations of MM-102, C646, and E034 for 24 h. Subsequently, 5 mg/mL MTT solution was added to each well, and the cells were incubated at 37 °C for 4 h. After incubation, the supernatant was removed, and 150 μL of DMSO was used to dissolve formazan crystals. Optical density (OD) was measured at a wavelength of 492 nm (Multiskan MK3; Thermo Fischer Scientific Corp).^28^^,^^29^

### Chromatin immunoprecipitation (ChIP) assay

The manufacturer's protocol was followed to prepare cell samples for analysis. SimpleChIP® Enzymatic Chromatin IP Kit was used to perform the ChIP assay. During immunoprecipitation, the following antibodies were used: rabbit monoclonal antibody specific for p-Stat3, rabbit monoclonal antibody specific for H3K27ac, mouse polyclonal antibody specific for P300, mouse polyclonal antibody specific for Cbp and normal rabbit IgG as a negative control for non-specific binding. Subsequently, a set of specific primers was used to purify the immunoprecipitated DNA, which was analysed using PCR to quantify enriched target DNA sequences.

### Co-Immunoprecipitation (Co-IP) assay

NP40 lysis buffer was added to the cells to prepare the samples. Rabbit anti-Trim21 antibody was added to bind the target protein. The mixture was incubated with rotation at 4 °C for 16 h. Protein A/G magnetic beads were used to capture the antibody–protein complexes. The captured proteins were eluted, and the presence of Trim21 was analysed by western blotting.

### Scanning electron microscopy (SEM)

The cells were cultured on 10-mm diameter glass slides in 1 mL of F12 medium containing 5% serum in 24-well plates. The samples were fixed with an electron microscope fixative solution (2.5% glutaraldehyde) for 4–12 h. The samples were rinsed in PBS and soaked in fresh PBS at room temperature for 10 min. This process was repeated three times. The solution was aspirated, and the samples were washed in 30%, 50%, 70%, 80%, and 90% ethanol at 4 °C for 5 min each. The solution was aspirated, 100% ethanol was added, and the samples were soaked at 4 °C for 10 min. This process was repeated twice. Critical point drying was immediately performed on the samples. After gold sputtering, images were collected using a microscope. The instruments used in the experiment were obtained from the Electron Microscopy Centre of Anhui Medical University. They included a scanning electron microscope (Gemini SEM 300, Carl Zeiss, Germany), a critical point dryer (QUORUM K850, Quorum, UK), and an ion sputterer (Cressington 108 Auto, Cressington, UK).

### Synthesis process for E034

Intermediate 3 was synthesised by subjecting raw material 1 and raw material 2 to Castro-Stephens coupling reaction, followed by de-BOC protection. Subsequently, Intermediate 3 was combined with raw material 4 in an acid-amine condensation reaction and de-BOC protection to yield Intermediate 5, which was then reacted with raw material 6 in an acid-amine condensation reaction to produce Intermediate 7, which was further derivatised through a deethylation reaction to yield degrader E034 (48.5 mg, white solid, yield: 55%, purity: 99.02%). Common chemicals were sourced from commercial suppliers and used as received without additional purification. The progression of the reactions was tracked using analytical thin-layer chromatography (TLC) on silica gel HSGF254 plates (Qingdao Haiyang Chemical Plant, China). Column chromatography was carried out using silica gel (200–300 mesh) obtained from Qingdao Dingkang Chemical Inc. 1H NMR, 19F NMR, and 31P NMR spectra were recorded in DMSO-d6 solution using Bruker 400 MHz NMR instrument (Bruker, Germany). Mass spectra were acquired using a QE Plus Hybrid Orbitrap high-resolution mass spectrometer (Thermo Fisher Scientific, USA). HPLC spectra were measured by an Agilent 1260 Series System equipped with a Cosmosil 5C18-AR-Ⅱ column (4.6 mm × 250 mm). Structural identification data are provided in Figures S9a and S10a–e.

### Statistical analysis

Data are expressed as mean ± standard error of the mean (SEM). The two-tailed Student's t-test was employed to compare the two groups. One-way ANOVA was utilised for analyses involving multiple groups. For multiple-group comparisons, the Kruskal–Wallis test was utilised. Statistical significance was set at a P-value of less than 0.05. Graphs were created using GraphPad Prism 5.0 (GraphPad Software Inc.).

### Role of the funding source

Funders had no role in the study design, data collection, data analyses, interpretation, or writing.

## Results

### Acetylation at H3K27 drives Stat3 upregulation

**Fig. 1:**
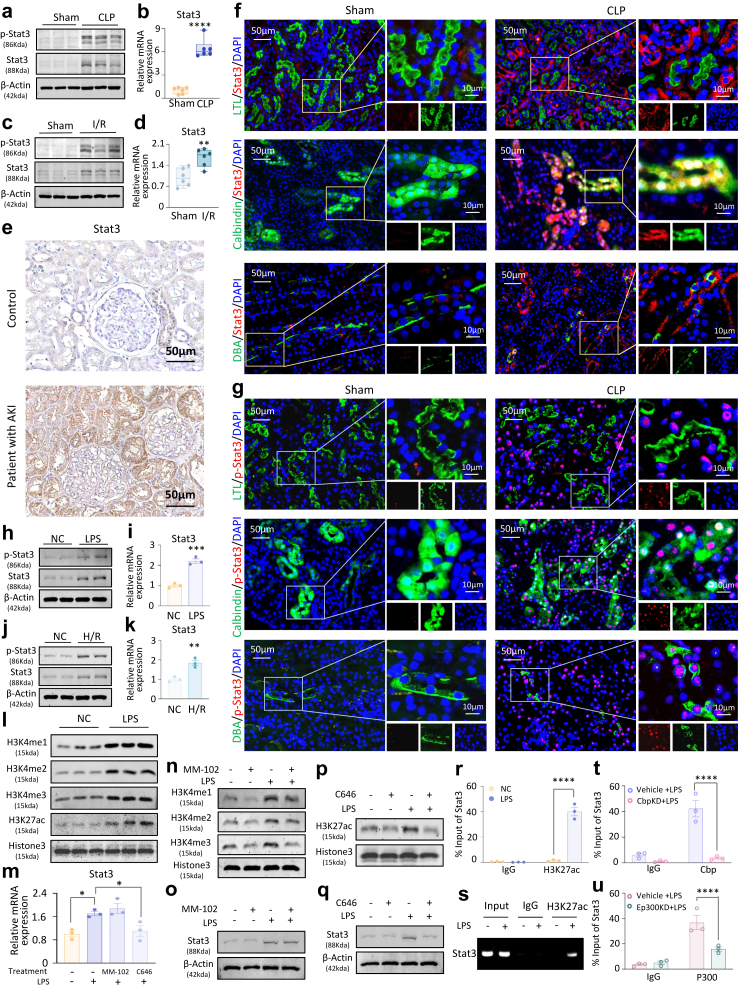
**Stat3 and p-Stat3 are markedly induced by H3K27ac modification in renal tubular cells of patients with AKI and CLP- and I/R-induced mouse models**. (a) Western blot analysis of Stat3 and p-Stat3 proteins in a CLP-induced AKI mouse model (n = 6). (b) Real-time PCR analysis of Stat3 mRNA levels in the CLP-induced AKI mouse model (n = 6, t-test). (c) Western blot analysis of Stat3 and p-Stat3 proteins in an I/R-induced AKI mouse model (n = 6). (d) Real-time PCR analysis of Stat3 mRNA levels in the I/R-induced AKI mouse model (n = 6, t-test). (e) IHC staining for Stat3 in patients with AKI (Scale bar = 50 μm). (f–g) Immunofluorescence staining for Stat3 and p-Stat3 with LTL, Calbindin, and DBA in the CLP-induced AKI mouse model (Scale bar = 50 μm and 10 μm; n = 6). (h) Western blot analysis of Stat3 and p-Stat3 proteins in mTECs treated with LPS (n = 3). (i) Real-time PCR analysis of Stat3 mRNA levels in LPS-treated mTECs (n = 3, t-test). (j) Western blot analysis of Stat3 and p-Stat3 proteins in mTECs subjected to H/R treatment (n = 3). (k) Real-time PCR analysis of Stat3 mRNA levels in H/R-treated mTECs (n = 3, t-test). (l) Western blot analysis of H3K4me1, H3K4me3, H3K27ac, and Histone 3 proteins in LPS-treated mTECs (n = 3). (m) Real-time PCR analysis of Stat3 mRNA levels after treatment with MM-102 and C646 following LPS induction (n = 3, one-way ANOVA). (n–o) Western blot analysis of H3K4me1, H3K4me2, H3K4me3, and Stat3 proteins after treatment with MM-102 following LPS induction (n = 3). (p–q) Western blot analysis of H3K27ac and Stat3 proteins after treatment with C646 following LPS induction (n = 3). (r–s) ChIP assay for the binding of H3K27ac to Stat3 (n = 3, one-way ANOVA). (t) ChIP assay for the binding of Cbp to Stat3 (n = 3, one-way ANOVA). (u) ChIP assay for the binding of P300 to Stat3 (n = 3, one-way ANOVA). (Data are presented as mean ± SEM; ∗P < 0.05, ∗∗P < 0.01, ∗∗∗P < 0.001, ∗∗∗∗P < 0.0001).

The prevailing view is that the phosphorylation-induced nuclear translocation of Stat3 modulates the transcription of downstream effectors, thereby precipitating renal injury. However, our research revealed an augmented expression of Stat3 not only at the phosphorylated protein level but also in terms of mRNA and total protein in mouse models of AKI induced by CLP and I/R injury (Fig. 1a–d and Figure S1a–b). Immunohistochemical analysis of renal biopsies from patients with AKI substantiated these findings (Fig. 1e). Using double immunofluorescence staining with markers specific to distinct segments of renal tubular epithelial cells (lotus tetragonolobus lectin [LTL] for proximal tubules, Calbindin for distal tubules, and dolichos biflorus agglutinin [DBA] for collecting ducts), we localised Stat3 and phosphorylated Stat3 predominantly in these cells (Fig. 1f–g and Figure S1c–d). *In vitro* studies using mTECs further corroborated these observations, demonstrating the upregulation of Stat3 mRNA, total protein, and phosphorylated protein under the influence of lipopolysaccharide (LPS) and hypoxia/reoxygenation (H/R) condition, mirroring the *in vivo* scenario (Fig. 1h–k and Figure S1e–f).

To clarify Stat3 upregulation in AKI, we conducted an in-depth investigation using the UCSC Genome Browser database (https://genome.ucsc.edu/). The promoter region of the Stat3 gene is subjected to epigenetic modifications, including methylation at lysine 4 (H3K4me) and acetylation at lysine 27 (H3K27ac) on histone H3. Consistently, we observed significant increase in the levels of H3K4me1, 2, 3, and H3K27ac modifications in both the CLP-induced AKI mouse models (Figure S2a) and LPS-induced mTECs *in vitro* (Fig. 1l). *In vitro*, we screened the optimal working concentrations of the methylation inhibitor MM102 and the acetylation inhibitor C646 (Figure S2b–g). After treatment, we observed a lack of acetylation modification at H3K27 rather than methylation at H3K4, which significantly diminished the upregulation of Stat3 mRNA and total protein levels (Fig. 1m–q). Furthermore, the ChIP assay confirmed more H3K27 acetylation in the AKI state (Fig. 1r–s). Further investigations revealed that silencing the histone acetyltransferase (HAT) P300/Cbp-encoding genes Ep300 and Crebbp by siRNAs led to a significant downregulation of Stat3 mRNA expression levels in renal tubular cells (Figure S2h–k). Concurrently, ChIP assays demonstrated that the silencing of P300 or Cbp protein expression markedly reduced their binding affinity to the promoter region of the Stat3 gene (Fig. 1t–u).

These findings suggest that in renal tubular cells, P300/Cbp enhances Stat3 gene transcriptional activity by catalysing the acetylation of histone H3 at lysine 27, thereby promoting chromatin remodelling. Specifically, the P300/Cbp-mediated H3K27 acetylation likely operates through the following mechanisms: First, P300/Cbp is recruited to the regulatory regions of the Stat3 gene, where it acetylates the H3K27 residue, alleviating chromatin repression; second, this epigenetic modification event enhances the binding efficiency of transcription factors to the Stat3 gene promoter, ultimately leading to elevated Stat3 transcription and a subsequent increase in its total protein expression.

### Stat3 cKO mitigates renal injury and inflammation

**Fig. 2:**
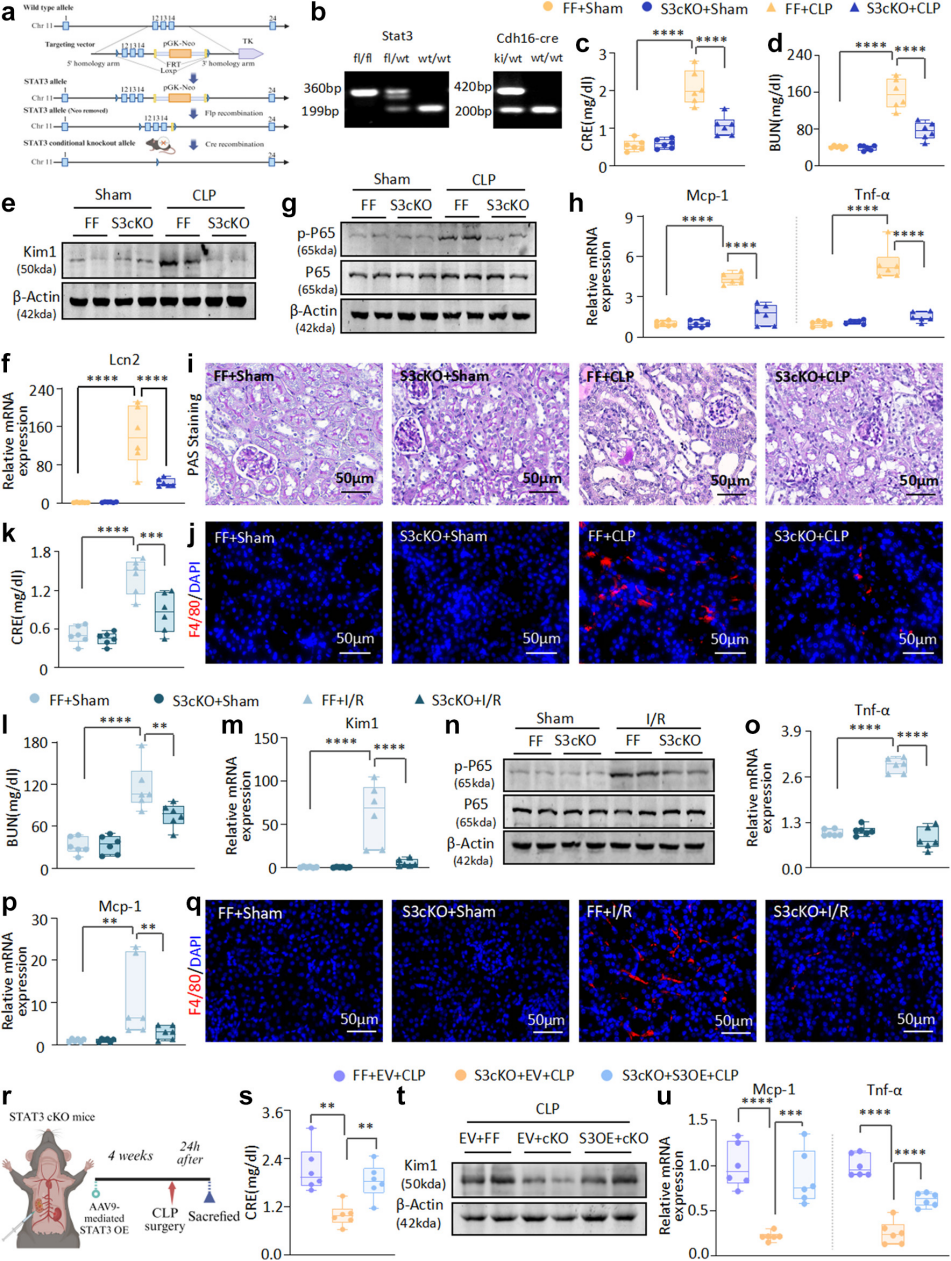
**Conditional knockout of Stat3 in renal tubular epithelial cells alleviates CLP- and I/R-induced kidney injury and inflammation in mice**. (a) The schematic diagram for constructing a mouse model with conditional knockout of Stat3 in renal tubular epithelial cells. (b) Validation of successful construction of Stat3 conditional knockout in renal tubular epithelial cells using agarose gel electrophoresis. (c–d) Serum CRE and BUN in the CLP-induced AKI mouse model with or without conditional knockout of Stat3. (e) Western blot analysis of Kim1 in the CLP-induced AKI mouse model. (f) Real-time PCR analysis of Lcn2 mRNA levels in the CLP-induced AKI mouse model. (g) Western blot analysis of p-P65 and P65 in the CLP-induced AKI mouse model. (h) Real-time PCR analysis of Mcp-1 and Tnf-α mRNA levels in the CLP-induced AKI mouse model. (i) PAS staining of kidney sections from Stat3^flox/flox^ and Stat3 conditional knockout mice with CLP-induced AKI. (j) IF staining of F4/80+ macrophage infiltration in mice with CLP-induced nephropathy with or without conditional knockout of Stat3. (k–l) Serum CRE and BUN in the I/R-induced AKI mouse model with or without conditional knockout of Stat3. (m) Real-time PCR analysis of Kim1 mRNA levels in I/R-induced AKI. (n) Western blot analysis of p-P65 and P65 in the I/R-induced AKI mouse model. (o) Real-time PCR analysis of Tnf-α mRNA levels in I/R-induced AKI. (p) Real-time PCR analysis of Mcp-1 mRNA levels in the I/R-induced AKI mouse model. (q) IF staining of F4/80+ macrophage infiltration in mice with I/R-induced nephropathy with or without conditional knockout of Stat3. (r) Schematic illustrating the rescue experiment in Stat3 conditional knockout mice. (s) Serum CRE in the CLP-induced AKI mouse model after the rescue of Stat3. (t) Western blot analysis of Kim1 in the CLP-induced AKI mouse model after rescue of Stat3. (u) Real-time PCR analysis of Mcp-1 and Tnf-α mRNA levels in the CLP-induced AKI mouse model after rescue of Stat3. (n = 6; Data are presented as mean ± SEM; ∗P < 0.05, ∗∗P < 0.01, ∗∗∗P < 0.001, ∗∗∗∗P < 0.0001, One-way ANOVA; Scale bar = 50 μm).

To elucidate the specific contributions of Stat3 in the context of AKI, we generated renal tubular epithelial cell Stat3 cKO mice (Fig. 2a–b and Figure S3a–d). Upon knockout of Stat3, our analyses revealed a profound reduction in CRE and BUN levels following CLP-induced AKI (Fig. 2c–d). Targeted deletion of Stat3 significantly suppressed the expression of renal injury markers, including kidney injury molecule-1 (Kim1) and lipocalin-2 (Lcn2) (Fig. 2e and f, Figure S3e). Furthermore, activation of the NF-κB signalling pathway, a critical mediator of inflammatory responses, was substantially attenuated in Stat3 cKO mice (Fig. 2g). In addition, the mRNA expression levels of the chemokine monocyte chemoattractant protein-1 (Mcp-1) and the inflammatory cytokine tumour necrosis factor-α (Tnf-α) were significantly reduced in the renal tissues of Stat3 cKO mice (Fig. 2h). Histopathological assessment demonstrated a marked reduction in renal tubular dilation and glycogen deposition, compared with the Stat3^flox/flox^ mice (Fig. 2i and Figure S3f). There was a noticeable decrease in F4/80+ macrophage infiltration in the kidneys of Stat3 cKO mice (Fig. 2j).

In parallel with the CLP model, Stat3 cKO in the I/R model reduced serum CRE and BUN levels vs. controls (Fig. 2k–l), decreased renal injury marker expression (Fig. 2m, Figure S3g and h), and improved renal histopathology (Figure S3i–j). Stat3 deficiency also suppressed the I/R-activated NF-κB pathway (Fig. 2n) and lowered inflammatory mediator mRNA levels (Fig. 2o–p and Figure S3k). Immunofluorescence showed it reduced F4/80+ macrophage infiltration after I/R (Fig. 2q). In contrast, Stat3 re-introduction (Fig. 2r and Figure S3l) caused a resurgence in renal function serologicals, injury pathologies, and inflammatory markers (Fig. 2s–u and Figure S3m–r), highlighting renal injury and inflammation recurrence.

### Stat3 mediates LPS- and H/R-induced cellular injury and inflammation in cultured tubular epithelial cells

**Fig. 3:**
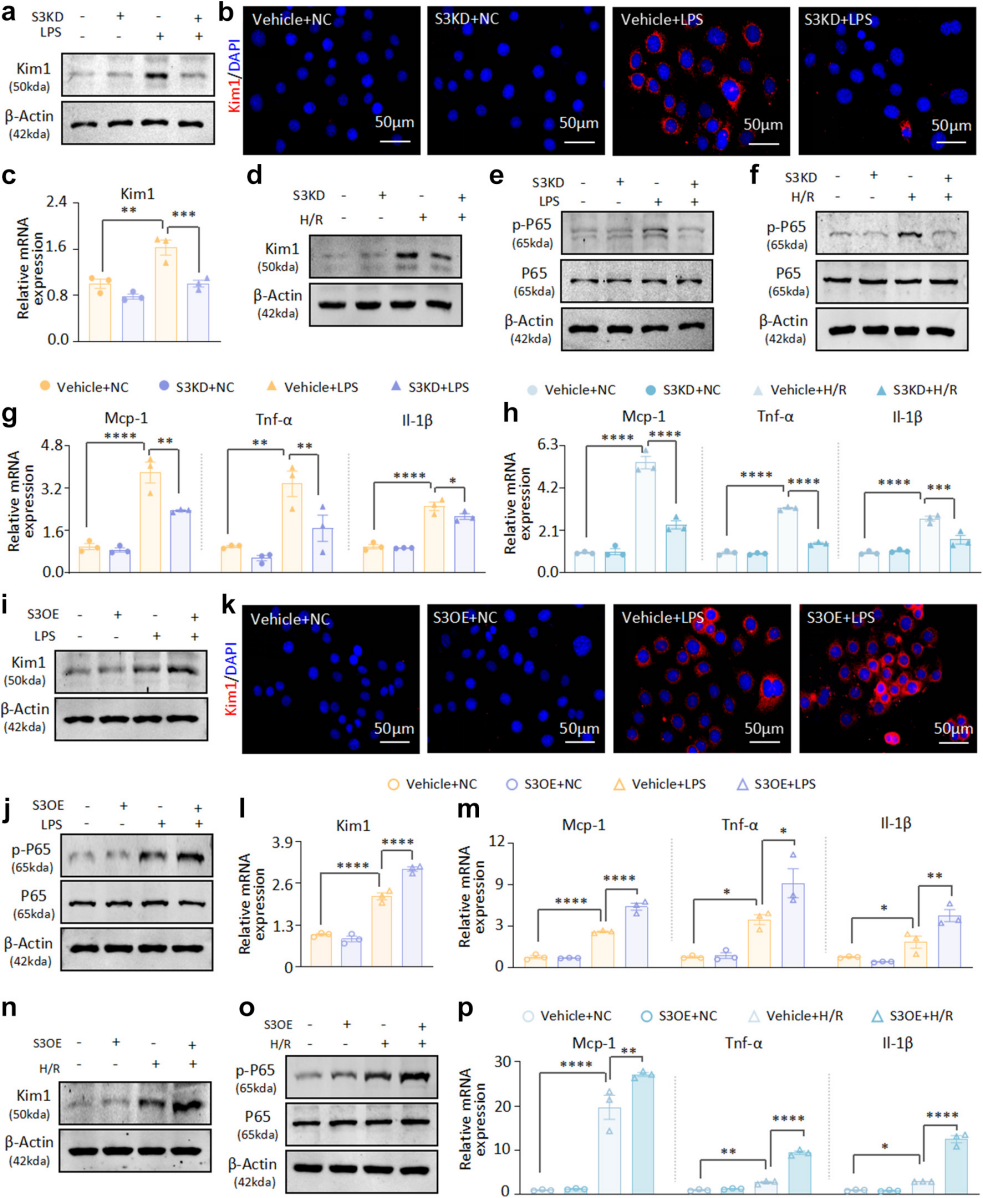
**Silencing Stat3 in mTECs and HK-2 *in vitro* alleviates LPS- and H/R-induced cell injury and inflammation, and Stat3 overexpression reverses these effects**. (a) Western blot analysis of Kim1 in LPS-induced cell models with or without knockdown of Stat3. (b) IF staining of Kim1 in LPS-induced cell models with or without knockdown of Stat3. (c) Real-time PCR analysis of Kim1 mRNA levels in LPS-induced cell models with or without knockdown of Stat3. (d) Western blot analysis of Kim1 in H/R-induced cell models with or without knockdown of Stat3. (e) Western blot analysis of p-P65 and P65 in LPS-induced cell models with or without knockdown of Stat3. (f) Western blot analysis of p-P65 and P65 in H/R-induced cell models with or without knockdown of Stat3. (g) Real-time PCR analysis of Mcp-1, Tnf-α, and Il-1β mRNA levels in LPS-induced cell models with or without knockdown of Stat3. (h) Real-time PCR analysis of Mcp-1, Tnf-α, and Il-1β mRNA levels in H/R-induced cell models with or without knockdown of Stat3. (i–j) Western blot analysis of Kim1, p-P65, and P65 in LPS-induced cell models with or without overexpression of Stat3. (k) IF staining of Kim1 in LPS-induced cell models with or without overexpression of Stat3. (l–m) Real-time PCR analysis of Kim1, Mcp-1, Tnf-α and Il-1β mRNA levels in LPS-induced cell models with or without overexpression of Stat3. (n–o) Western blot analysis of Kim1, p-P65, and P65 in H/R-induced cell models with or without overexpression of Stat3. (p) Real-time PCR analysis of Mcp-1, Tnf-α, and Il-1β mRNA levels in H/R-induced cell models with or without overexpression of Stat3. (n = 3; Data are presented as mean ± SEM; ∗P < 0.05, ∗∗P < 0.01, ∗∗∗P < 0.001, ∗∗∗∗P < 0.0001, one-way ANOVA; Scale bar = 50 μm).

To dissect the intricate regulatory functions of Stat3 in renal tubular epithelial cells under LPS and H/R stressors, we performed *in vitro* assays using mTECs and HK-2. Our findings demonstrate that upon targeted inhibition of Stat3 expression in mTECs (Figure S4a–b), the upregulation of Kim1 mRNA and protein levels induced by LPS and H/R was significantly suppressed (Fig. 3a–d and Figure S4c). In synchrony, we observed a marked reduction in the phosphorylation and activation of the P65 subunit of the NF-κB signalling cascade in response to Stat3 knockdown (Fig. 3e–f). This molecular modulation was parallelled by a significant decrease in the mRNA levels of inflammatory mediators, including Mcp-1, Tnf-α, and Il-1β, leading to a pronounced amelioration of cellular inflammatory profiles (Fig. 3g–h). In contrast, overexpression of Stat3 amplified LPS-induced cellular injury and escalated the inflammatory cascade orchestrated by the NF-κB pathway (Fig. 3i–l, Figure S4d–e). This overexpression led to upregulation of Mcp-1, Tnf-α and Il-1β mRNA levels, thereby accentuating the inflammatory sequelae (Fig. 3m). Similar results were reproduced in H/R-induced *in vitro* models, confirming the essential role of Stat3 in regulating cellular responses to harmful stimuli (Fig. 3n–p and Figure S4f). The functional role of STAT3 was further validated *in vitro* using HK-2 cells. It is revealed that the genetic knockdown of STAT3 significantly attenuated the expression of kidney injury markers, whereas its overexpression exacerbated these phenotypes (Figure S4g–j).

### Trim21 is a direct target of Stat3

**Fig. 4:**
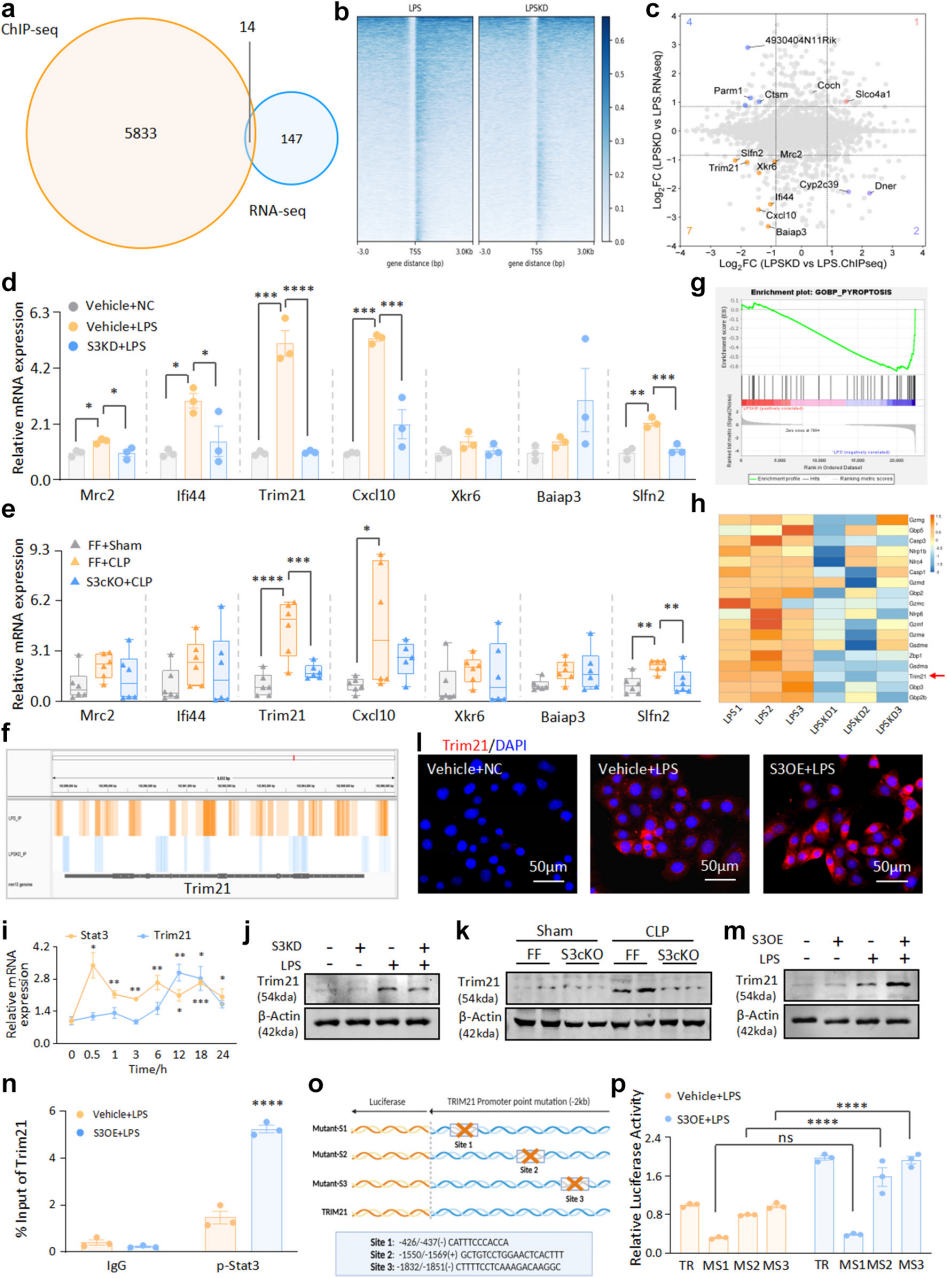
**Combined ChIP-seq and RNA-seq analysis screens Trim21, a target gene that directly binds to its promoter region and regulates transcription**. (a) Venn diagram of intersecting genes between ChIP-seq and RNA-seq analysis. (b) Comparison of TSS peak plots for samples undergoing ChIP-seq. (c) Quadrant plot of intersecting genes from ChIP-seq and RNA-seq. (d) Real-time PCR analysis of Mrc2, Ifi44, Trim21, Cxcl10, Xkr6, Baiap3, and Slfn2 mRNA levels in LPS-induced cell models with or without knockdown of Stat3 (n = 3). (e) Real-time PCR analysis of Mrc2, Ifi44, Trim21, Ccxl10, Xkr6, Baiap3, and Slfn2 mRNA levels in CLP-induced AKI models with or without conditional knockout of Stat3 (n = 6). (f) Visualisation of Trim21 ChIP-seq data. (g) GSEA of RNA-seq results for the pyroptosis pathway. (h) Heatmap of genes related to the pyroptosis pathway. (i) Real-time PCR analysis of the dynamic mRNA levels of Stat3 and Trim21 for 0–24 h in LPS-induced cell models with or without knockdown of Stat3 (n = 3). (j) Western blot analysis of Trim21 with or without knockdown of Stat3 in LPS-induced cell models (n = 3). (k) Western blot analysis of Trim21 in Stat3^flox/flox^ and Stat3 cKO mice with CLP-induced AKI (n = 6). (l) IF staining of Trim21 in LPS-induced cell models with or without knockdown of Stat3 (n = 3). (m) Western blot analysis of Trim21 with or without overexpression of Stat3 in LPS-induced cell models (n = 3). (n) Binding of Stat3 to Trim21 assessed by ChIP assay (n = 3). (o) Schematic illustration of mutations in the Trim21 promoter region. (p) Dual-luciferase reporter assay analysing the effect of mutations at different sites in the Trim21 promoter region on binding affinity (n = 3). (Data are presented as mean ± SEM; ∗P < 0.05, ∗∗P < 0.01, ∗∗∗P < 0.001, ∗∗∗∗P < 0.0001, one-way ANOVA; Scale bar = 50 μm).

mTECs were LPS-treated with Stat3-silenced and control groups, while ChIP-seq and RNA-seq were used to identify the downstream targets. Convergent analysis revealed a cohort of 14 intersecting genes (Fig. 4a), which narrowed our focus to seven protein-coding genes that exhibited concurrent downregulation in binding strength and transcriptional output upon Stat3 suppression (Fig. 4b–c). Through a combination of *in vitro* and *in vivo* low-flux screening coupled with peak profiling, we identified significant upregulation of tripartite motif-containing protein 21 (Trim21) mRNA levels in our experimental cohort, which was found to be regulated by Stat3 (Fig. 4d–f). Gene set enrichment analysis (GSEA) of RNA-seq data demonstrated a robust correlation with the pyroptosis pathway, consistent with our validation findings (Fig. 4g–h). Temporal upregulation of Trim21 mRNA was after that of Stat3 (Fig. 4i), and both *in vitro* knockdown of mTECs and HK-2 (Fig. 4j and Figure S5a) and *in vivo* Stat3 cKO (Fig. 4k) markedly attenuated Trim21 protein levels. In contrast, its overexpression increased Trim21 (Fig. 4l–m and Figure S5b). ChIP assays further confirmed the direct binding of p-Stat3 to Trim21 (Fig. 4n), suggesting that Trim21 is a potential direct target.

Leveraging the comprehensive resource of Animal TFDB v4.0 (https://guolab.wchscu.cn/AnimalTFDB4/#/), we constructed the mutant variants MS1 (site 1 absent), MS2 (site 2 absent) and MS3 (site 3 absent) (Fig. 4o). After LPS stimulation, Stat3 overexpression enhanced wild-type Trim21 promoter and MS2/MS3 mutant luciferase activities, but MS1 mutant showed no significant change. It suggested that LPS-induced phosphorylated Stat3 entered the nucleus and interacted with the −426 to −437 bp segment of the Trim21 promoter, promoting transcriptional activation (Fig. 4p).

### Trim21 promotes LPS-induced cell injury, inflammation, and pyroptosis

**Fig. 5:**
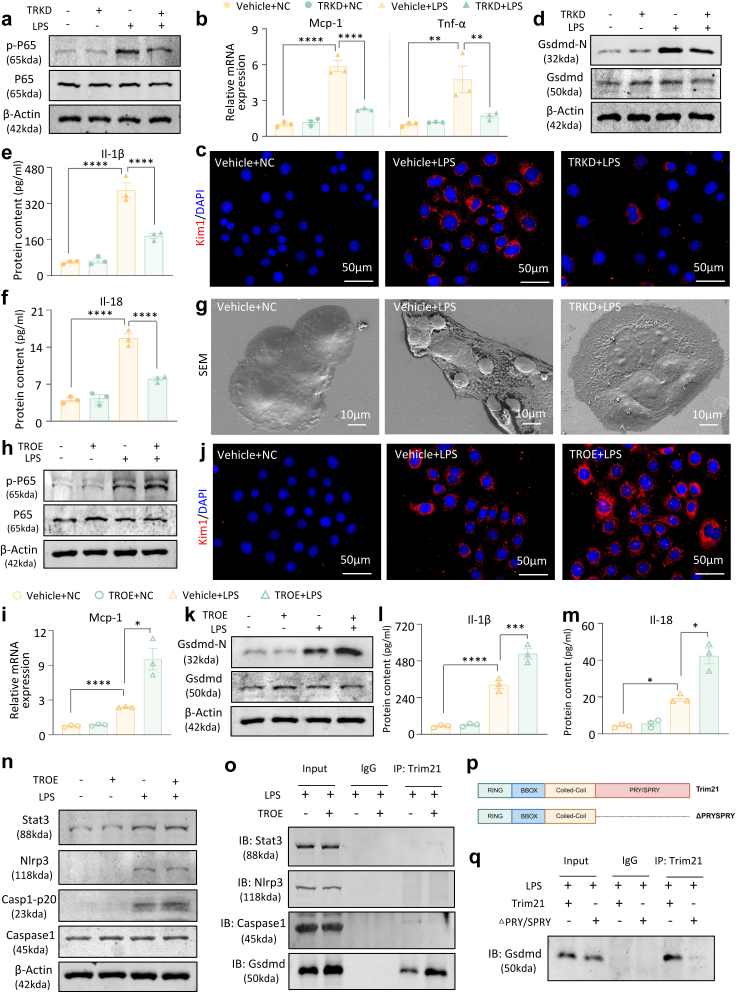
**Trim21 bypasses the inflammasome pathway and binds to Gsdmd through the PRY/SPRY domain, promoting LPS-induced cell injury, inflammation, and pyroptosis**. (a) Western blot analysis of p-P65 and P65 in LPS-induced cells with or without knockdown of Trim21. (b) Real-time PCR analysis of Mcp-1 and Tnf-α mRNA levels in LPS-induced cells with or without knockdown of Trim21. (c) IF staining of Kim1 in LPS-induced cells with or without knockdown of Stat3 (Scale bar = 50 μm). (d) Western blot analysis of Gsdmd-N and Gsdmd in LPS-induced cells with or without knockdown of Trim21. (e–f) ELISA for determining Il-1β and Il-18 levels in the supernatant of LPS-induced cells with or without knockdown of Trim21. (g) SEM morphology observation of LPS-induced cells with or without knockdown of Trim21 (Scale bar = 10 μm). (h) Western blot analysis of p-P65 and P65 in LPS-induced cells with or without overexpression of Trim21. (i) Real-time PCR analysis of Mcp-1 mRNA levels in LPS-induced cells with or without overexpression of Trim21. (j) IF staining of Kim1 in LPS-induced cells with or without overexpression of Trim21 (Scale bar = 50 μm). (k) Western blot analysis of Gsdmd-N and Gsdmd in LPS-induced cells with or without overexpression of Trim21. (l–m) ELISA for determination of Il-1β and Il-18 levels in the supernatant of LPS-induced cell models with or without overexpression of Trim21. (n) Western blot analysis of Stat3, Nlrp3, caspase1-p20 and caspase1 in LPS-induced cells with or without overexpression of Trim21. (o) Co-IP assay for the binding of Trim21 to Stat3, Nlrp3, Caspase1, and Gsdmd in LPS-induced cells with or without overexpression of Trim21. (p) Schematic illustration of Trim21 and the ΔPRY/SPRY domain. (q) Co-IP assay for Trim21 or ΔPRY/SPRY binding to Gsdmd in LPS-induced cell models. (n = 3; Data are presented as mean ± SEM; ∗P < 0.05, ∗∗P < 0.01, ∗∗∗P < 0.001, ∗∗∗∗P < 0.0001, one-way ANOVA).

Ulteriorly, we found that the knockdown of Trim21 substantially abrogated the activation of the NF-κB signalling pathway (Figure S6a–b and Fig. 5a), leading to a significant reduction in the mRNA expression of Mcp-1 and Tnf-α (Fig. 5b) as well as a decrease in Kim1 synthesis in mTECs (Fig. 5c and Figure S6c). It suggested an ameliorative effect on LPS-induced cellular injury and inflammatory response *in vitro*.

Additionally, shearing activation of gasdermin D (Gsdmd) was markedly attenuated (Fig. 5d), concurrent with the reduced secretion of the pro-inflammatory cytokines Il-1β and Il-18 (Fig. 5e–f). By SEM, LPS-treated mTECs showed pyroptotic changes like nuclear protrusion and membrane porosity. However, after the Trim21 knockdown, cells maintained their morphology, and membranes had no porosity, suggesting that the pyroptotic features were much relieved (Fig. 5g). In contrast, Trim21 overexpression led to increased phosphorylation of P65 (Figure S6d–e and Fig. 5h) and increased mRNA levels of Mcp-1 (Fig. 5i), which exacerbated cellular injury (Fig. 5j and Figure S6f). Moreover, Trim21 overexpression intensified Gsdmd cleavage (Fig. 5k) and induced the activation of Il-1β and Il-18 (Fig. 5l–m), indicating a pro-inflammatory and pro-pyroptotic role of Trim21.

As an E3 ubiquitin ligase, Trim21 did not facilitate ubiquitination and degradation of Stat3 in mTECs (Fig. 5n–o), and Trim21 also did not interact with or modulate the expression levels of Nlrp3 and Caspase-1 (Fig. 5n–o), signifying that the role of Trim21 in pyroptosis is independent of the inflammasome signalling pathway. To evaluate the role of Caspase1 in Gsdmd activation, we investigated the specific regulatory effect of Trim21 on Gsdmd cleavage using the Caspase1-specific inhibitor VX-765.^30^^,^^31^ The optimal working concentration was determined using a combination of MTT and Western blot assays *in vitro* renal tubular epithelial cells (Figure S6g–h). Functional assays revealed that pharmacological inhibition of Caspase1 activity did not significantly alter the N-terminal cleavage of Gsdmd (Figure S6i), indicating that under conditions of high Trim21 induction, the proteolytic activation of Gsdmd may be mediated by Trim21 rather than dependent on Caspase1. The PRY/SPRY domain is a salient feature of Trim21, which is instrumental in mediating protein–protein interactions and enables Trim21 to engage with specific target proteins^32^^,^^33^ selectively. Our findings revealed that the absence of the PRY/SPRY domain in Trim21 resulted in a loss of binding to Gsdmd (Fig. 5p–q), corroborating existing studies.^34^ These observations suggest that Trim21 directly interacts with Gsdmd through its PRY/SPRY domain, bypassing the inflammasome pathway and promoting N-terminal cleavage and activation, thereby regulating the pyroptotic process in renal tubular cells.

### Stat3/Trim21/Gsdmd axis promotes cell damage and pyroptosis

**Fig. 6:**
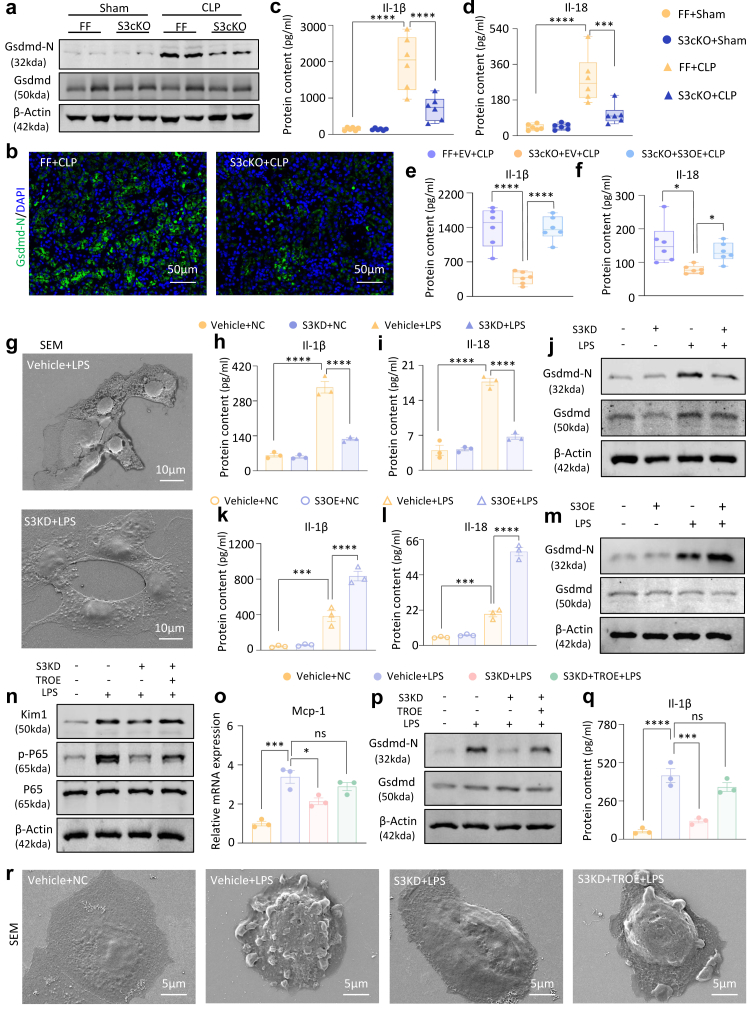
**Stat3 stimulates LPS-induced pyroptosis by promoting Trim21 transcription**. (a) Western blot analysis of Gsdmd-N and Gsdmd in Stat3^flox/flox^ and Stat3 cKO mice with CLP-induced AKI. (b) IF staining of Gsdmd-N in Stat3^flox/flox^ and Stat3 cKO mice with CLP-induced AKI(Scale bar = 50 μm). (c–d) ELISA for determining Il-1β and Il-18 levels in the serum from Stat3^flox/flox^ and Stat3 cKO mice with CLP-induced AKI (n = 6). (e–f) ELISA for the determination of Il-1β and Il-18 levels in the serum from Stat3 cKO mice with CLP-induced AKI with or without Stat3 rescue (n = 6). (g) SEM morphology observation of LPS-induced cells with or without knockdown of Stat3 (Scale bar = 10 μm). (h–i) ELISA for determining Il-1β and Il-18 levels in the supernatant of LPS-induced cells with or without knockdown of Stat3 (n = 3). (j) Western blot analysis of Gsdmd-N and Gsdmd in LPS-induced cells with or without knockdown of Stat3 (n = 3). (k–l) ELISA for determining Il-1β and Il-18 levels in the supernatant of LPS-induced cells with or without overexpression of Stat3 (n = 3). (m) Western blot analysis of Gsdmd-N and Gsdmd in LPS-induced cells with or without overexpression of Stat3 (n = 3). (n) Western blot analysis of Kim1, p-65 and P65 with or without Trim21 rescue after Stat3 knockdown in the LPS-induced cells (n = 3). (o)Real-time PCR analysis of Mcp-1 mRNA levels in the supernatant of LPS-induced cells with or without Trim21 rescue after Stat3 knockdown (n = 3). (p) Western blot analysis of Gsdmd-N and Gsdmd with or without Trim21 rescue after Stat3 knockdown in the LPS-induced cells (n = 3). (q) ELISA for the determination of Il-1β level in the supernatant of LPS-induced cells with or without Trim21 rescue after Stat3 knockdown (n = 3). (r) SEM morphology observation of LPS-induced cells with or without Trim21 rescue after Stat3 knockdown (Scale bar = 5 μm). (Data are presented as mean ± SEM; ∗P < 0.05, ∗∗∗P < 0.001, ∗∗∗∗P < 0.0001, one-way ANOVA).

In further studies employing Stat3 cKO mice, we observed a significant downregulation of Gsdmd-N protein levels following Stat3 ablation (Fig. 6a–b), concomitant with markedly suppressed activation of Il-1β and Il-18 (Fig. 6c–d). This protective effect was abolished following endogenous reconstitution of Stat3 (Fig. 6e–f). Notably, the cellular pyroptosis phenotype was ameliorated upon Stat3 deficiency (Fig. 6g), along with attenuated activation of Il-1β, Il-18 and Gsdmd (Fig. 6h–j), while Stat3 overexpression *in vitro* resulted in increased their activation (Fig. 6k–m).

Then, we performed rescue experiments by re-introducing Trim21 into Stat3-silenced mTECs. Trim21 overexpression restored the mRNA expression level of Kim1, activation of phosphorylated P65, and expression of Mcp-1 in the absence of Stat3 (Fig. 6n–o). Additionally, the activation and secretion of Gsdmd, Il-1β and Il-18 were restored (Fig. 6p–q and Figure S7a). SEM provided visual evidence (Fig. 6r). Consistent with these findings, analogous results were observed in HK-2 cells (Figure S7b–e). Conversely, following Stat3 overexpression, subsequent silencing of Trim21 led to significant attenuation in activating the NF-κB signalling pathway and the transcriptional levels of renal injury markers and pro-inflammatory cytokines (Figure S7f–h). Collectively, these findings reinforce the regulatory role of Stat3 in the modulation of Trim21-mediated inflammatory responses and pyroptosis pathways.

### Renal protection via AAV9-Delivered Stat3 silencing in AKI mice

**Fig. 7:**
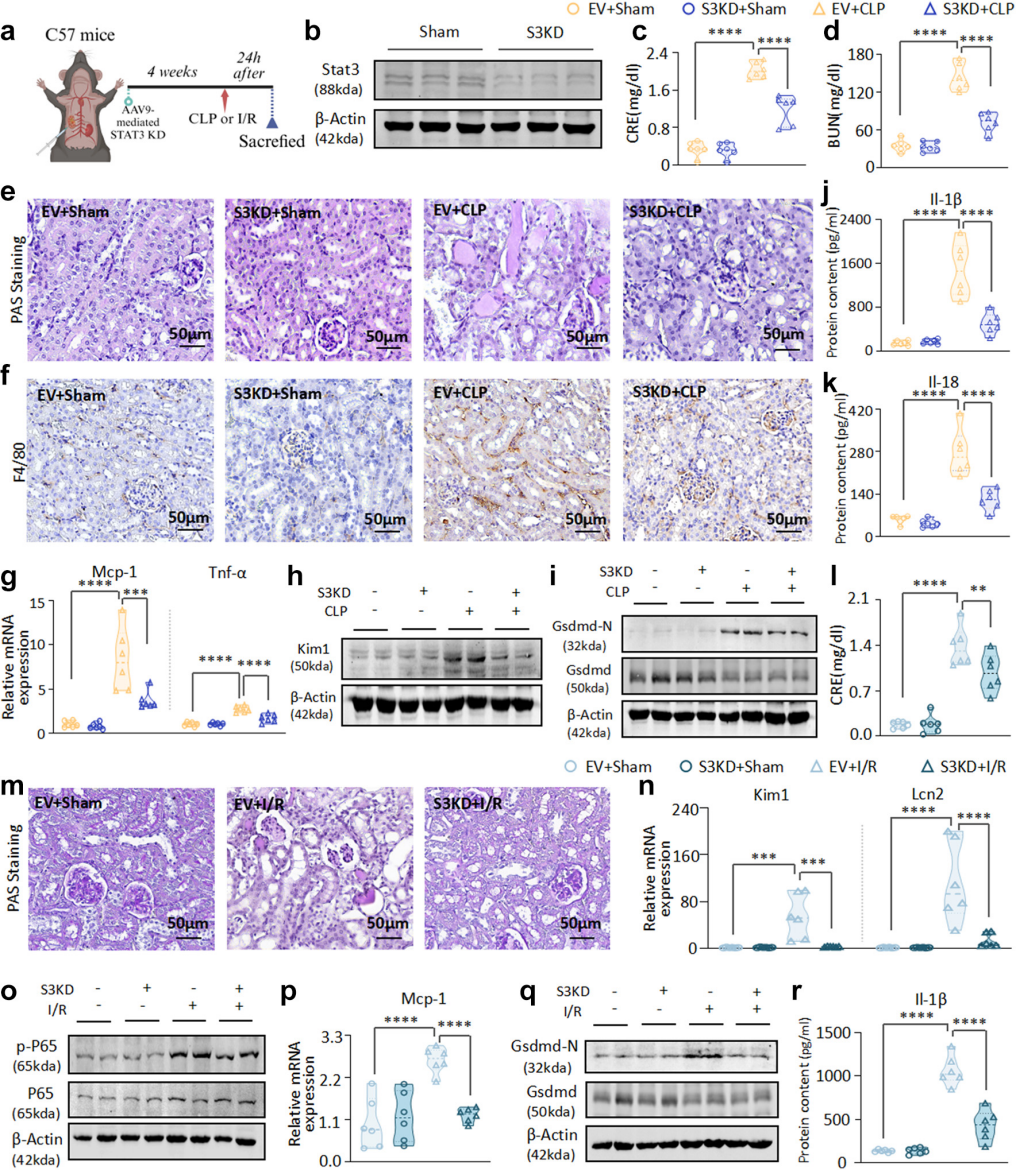
**AAV9-mediated *in vivo* silencing of Stat3 attenuates CLP- and I/R-induced renal injury, inflammatory response, and pyroptosis**. (a) Schematic diagram of *in vivo* AAV9-mediated silencing of Stat3 in mice. (b) Western blot analysis of Stat3 in mice with or without knockdown of Stat3. (c–d) Serum CRE and BUN in CLP-induced AKI mice with or without knockdown of Stat3. (e) PAS staining of kidney sections in CLP-induced AKI mice with or without knockdown of Stat3. (f) IHC staining of F4/80+ macrophage infiltration in mice with CLP-induced nephropathy with or without knockdown of Stat3. (g) Real-time PCR analysis of Mcp-1 and Tnf-α mRNA levels in CLP-induced AKI mice with or without knockdown of Stat3. (h–i) Western blot analysis of Kim1, Gsdmd-N and Gsdmd in CLP-induced AKI mice with or without knockdown of Stat3. (j–k) ELISA for determining Il-1β and Il-18 levels in the serum from CLP-induced AKI mice with or without knockdown of Stat3. (l) Serum CRE in I/R-induced AKI mice with or without knockdown of Stat3. (m) PAS staining of kidney sections in I/R-induced AKI mice with or without knockdown of Stat3. (n) Real-time PCR analysis of Kim1 and Lcn2 mRNA levels in I/R-induced AKI mice with or without knockdown of Stat3. (o) Western blot analysis of p-P65 and P65 in I/R-induced AKI mice with or without knockdown of Stat3. (p) Real-time PCR analysis of Mcp-1 mRNA level in I/R-induced AKI mice with or without knockdown of Stat3. (q) Western blot analysis of Gsdmd-N and Gsdmd in I/R-induced AKI mice with or without knockdown of Stat3. (r) ELISA for determining Il-1β level in the serum from I/R-induced AKI mice with or without knockdown of Stat3. (n = 3; Data are presented as mean ± SEM; ∗∗P < 0.01, ∗∗∗P < 0.001, ∗∗∗∗P < 0.0001, one-way ANOVA; Scale bar = 50 μm).

To assess the therapeutic efficacy of Stat3 inhibition in AKI mouse models, AAV9 with a Stat3 silencing plasmid controlled by a renal tubular cell-specific promoter was used for targeted Stat3 suppression in murine kidney tubular cells (Fig. 7a–b and Figure S8a–b). The depletion of Stat3 led to a significant reduction in the serum CRE and BUN levels (Fig. 7c–d). PAS staining confirmed the attenuation of renal injury (Fig. 7e). Furthermore, renal infiltration of F4/80+ macrophages was inhibited (Fig. 7f), along with diminished expression of the chemokine and the pro-inflammatory cytokines (Fig. 7g and Figure S8c). The expression of Kim1 at both the mRNA and protein levels was notably reduced (Fig. 7h and Figure S8d), and the activation of the NF-κB inflammatory signalling pathway was suppressed (Figure S8e). Concurrently, Stat3 knockdown mitigated the cleavage of Gsdmd (Fig. 7i) and hindered the activation and release of Il-1β and Il-18 (Fig. 7j–k). Similarly, renal Stat3 deficiency significantly lowered the expression of serological and pathological indicators of renal injury, inflammation, and necrosis triggered by I/R, thereby retarding AKI progression (Fig. 7l–r and Figure S8f–i). The results showed that Stat3 inhibition protects against AKI progression.

### Stat3 PROTAC E034 confers renal protection both *in vitro* and *in vivo*

**Fig. 8:**
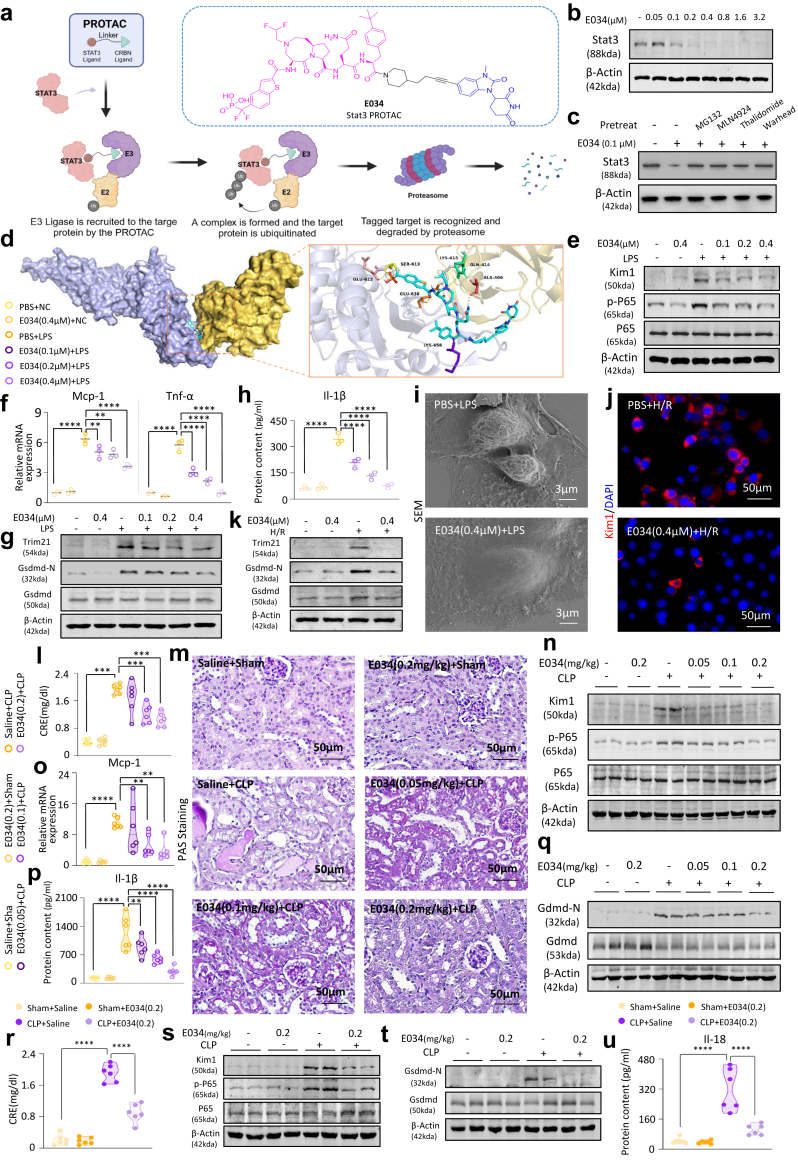
**PROTAC E034 mitigates CLP-induced kidney injury, inflammation, and pyroptosis by degrading Stat3**. (a) Structural formula and mechanism of action of E034. (b) Western blot analysis of Stat3 under the influence of different doses of E034. (c) Western blot analysis of Stat3 with pre-treatment of MG132, MLN4924, thalidomide, and warhead. (d) Computational simulation of molecular docking between Stat3 and E034. (e) Western blot analysis of Kim1, p-P65 and P65 under 0.1, 0.2 and 0.4 μM doses of E034 in LPS-induced cells. (f) Real-time PCR analysis of Mcp-1 and Tnf-α mRNA levels in LPS-induced cells (n = 3). (g) Western blot analysis of Trim21, Gsdmd-N and Gsdmd in LPS-induced cells after treatment with 0.1, 0.2 and 0.4 μM E034. (h) ELISA for the determination of Il-1β levels in the supernatant of LPS-induced cells (n = 3). (i) SEM morphology observation of LPS-induced cells with or without 0.4 μM E034 treatment (Scale bar = 3 μm). (j) IF staining of Kim1 in H/R-induced cells after treatment with 0.4 μM E034 (Scale bar = 50 μm). (k) Western blot analysis of Trim21, Gsdmd-N and Gsdmd in H/R-induced cells. (l) Serum CRE in CLP-induced AKI mice after pre-treatment (n = 6). (m) PAS staining of kidney sections in CLP-induced AKI mice after pre-treatment with 0.05, 0.1 and 0.2 mg/kg E034. (n) Western blot analysis of Kim1, p-P65 and P65 in CLP-induced AKI mice after pre-treatment. (o) Real-time PCR analysis of Mcp-1 mRNA levels in CLP-induced AKI mice (n = 6). (p) ELISA for determining Il-1β levels in serum from CLP-induced AKI mice (n = 3). (q) Western blot analysis of Gsdmd-N and Gsdmd in CLP-induced AKI mice. (r) Serum CRE in CLP-induced AKI mice (n = 6). (s–t) Western blot analysis of Kim1, p-P65, P65, Gsdmd-N and Gsdmd in CLP-induced AKI mice after treatment with 0.2 mg/kg E034. (u) ELISA for determining Il-18 levels in the serum from CLP-induced AKI mice (n = 6). (Data are presented as mean ± SEM; ∗∗P < 0.01, ∗∗∗P < 0.001, ∗∗∗∗P < 0.0001, one-way ANOVA).

Given the limitations of current Stat3 small-molecule inhibitors and our prior finding of substantial total Stat3 upregulation, there is an urgent demand for more comprehensive Stat3 inhibitory approaches. We identified a potent Stat3-targeting PROTAC, E034 (Fig. 8a, FIgures S9 and S10a–e) to address this. E034 displayed negligible toxicity at concentrations below 12.8 μM, with a DC50 value of 109.50 nM and a Dmax of 94.69% (Figure S11a and Fig. 8b). Treatment with a proteasome inhibitor (MG132) and a NEDD8-activating E1 enzyme inhibitor (MLN4924) abolished the degradation of Stat3 induced by E034, indicating the involvement of the UPS in E034-mediated degradation (Fig. 8c). Competitive inhibition of Stat3 degradation by either the warhead (Stat3 ligand) or thalidomide (Cereblon [CRBN] ligand) suggests that E034 functions as a bivalent molecule (Fig. 8c). Furthermore, E034 effectively induced Stat3 downregulation following pre-treatment with the protein synthesis inhibitor cycloheximide (CHX) for 16 h, underscoring its efficacy as a chemical degrader (Figure S11b). Notably, the effective degradation of Stat3 by PROTAC E034 relies on forming favourable ternary complexes as a key requirement. The simulated structure of the Stat3-E034-CRBN ternary complex strongly supports the beneficial role of E034 in Stat3 degradation (Fig. 8d). Through comparative analysis with other members of the Stat family, including Stat1, Stat5, and Stat6, E034 demonstrated high specificity for Stat3 (Figure S11c).

We found that pre-treatment with E034 before LPS-induced cell damage caused a dose-dependent decrease in Kim1 (Fig. 8e and Figure S11d), phosphorylation of P65 (Fig. 8e), and mRNA levels of Mcp-1 and Tnf-α (Fig. 8f) in mTECs. Concurrently, the expression of the downstream effector molecule Trim21 was significantly diminished upon Stat3 degradation (Fig. 8g). Moreover, the activation of the pyroptotic effector molecules (Fig. 8g–h and Figure S11e) also exhibited a dose-dependent decrease under the influence of E034. These findings, in conjunction with the morphological assessments conducted using SEM (Fig. 8i), suggest that E034 can effectively mitigate LPS-induced cellular injury, inflammation, and pyroptosis. Parallelling these observations, we also established that E034 ameliorated cellular inflammation and pyroptosis in an *in vitro* H/R-induced cell injury model (Fig. 8j–k and Figure S11f–j).

In subsequent studies, we established drug concentration gradients in murine models and revealed a non-toxic and therapeutically active dosage gradient ranging from 0.05 to 0.8 mg/kg (Figure S12a–b). We then delineated three gradient dose groups, 0.05, 0.1, and 0.2 mg/kg, which were administered intraperitoneally to mice 12 h before CLP surgery (Figure S12c). Intervention with E034 resulted in a dose-dependent decrease in the serum CRE and BUN levels compared with the CLP model group (Fig. 8l and Figure S12d), along with significant improvements in renal glycogen deposition and tubular dilation (Fig. 8m and Figure S12e). Kim1, p-P65, and mRNA levels of Mcp-1 exhibited a dose-dependent reduction (Fig. 8n–o, Figure S12f), along with attenuation of renal infiltration of F4/80+ macrophages (Figure S12g). Following Stat3 degradation, the activation of Il-1β, Gsdmd and Il-18 was markedly suppressed (Fig. 8p–q and Figure S12h). We then administered E034 intraperitoneally to CLP-induced mice 2h postoperatively (Figure S13a). The treatment group exhibited lower serum Cre and BUN levels than the CLP model group (Fig. 8r and Figure S13b), with notable amelioration of renal glycogen deposition and tubular dilation (Figure S13c–d). Furthermore, E034 mitigated kidney injury, inflammatory responses, and renal cell pyroptosis (Fig. 8s–u and Figure S13e), reinforcing its potential as a renoprotective agent against AKI.

Concurrently, we evaluated aspartate aminotransferase (AST) and alanine aminotransferase (ALT) in mice given 0.2 mg/kg (Figure S14a). Results showed no significant difference from controls, indicating no hepatotoxicity. Furthermore, the H&E staining of vital organs, including the heart, liver, spleen, and lungs, revealed no discernible detrimental effects, confirming no significant toxicity of E034 within the tested dosage range (Figure S14b).

## Discussion

Although most previous studies have considered Stat3 phosphorylation and activation as amplifying factors in the development of acute and chronic kidney injury,^13^, ^14^, ^15^ many studies hold opposing views. These discrepancies may be closely associated with modification patterns and the severity of AKI. In existing research, the functional role of Stat3 has primarily been explored by inhibiting its phosphorylation activation in the whole kidney. However, a critical, overlooked issue is that suppressing Stat3 phosphorylation may lead to unpredictable changes in other protein forms. Evidence indicates that non-phosphorylated post-translational modifications are crucial for Stat3 transcriptional activation. For instance, palmitoylation of Stat3 promotes its dimerisation and enhances transcriptional activity, driving TH17 cell differentiation in inflammatory bowel disease^35^; acetylation of Stat3 is known to inhibit its ubiquitination, thereby avoiding proteasomal degradation, prolonging its intracellular half-life, and regulating its residence time and activity.^36^ We employed a renal tubular cell-specific Stat3 knockout mouse model to address the need for more comprehensive research approaches. We observed that targeted deletion of Stat3 significantly delayed the progression of renal injury induced by severe sepsis and 40-min bilateral renal ischaemia-reperfusion. Following AKI, the kidney initiates an autonomous repair process. In early, mild renal injury models, such as unilateral renal artery-vein clamping by Xu et al.^18^ and 30-min bilateral renal clamping by Fang et al.^37^ Stat3 protects renal function by regulating mitochondrial apoptosis in tubular cells or up-regulating Survivin to promote cell survival. However, as demonstrated in our study, when injury exceeds a certain threshold, a surge in pro-inflammatory cytokines may feedback to activate P300/Cbp analogues, mediating acetylation of histone H3K27 at the Stat3 locus, triggering its excessive transcription, and activating downstream damage pathways, thereby exacerbating AKI.

The therapeutic landscape for modulating Stat3 activity in renal injury has been dominated by small-molecule inhibitors, which primarily target phosphorylation and dimerisation–key processes for Stat3 transcriptional activation and DNA-binding capacity.^38^^,^^39^ However, this strategy faces critical limitations. Dynamic conformational changes in Stat3 domains during activation alter ligand-binding affinities, complicating inhibitor design and clinical predictability. Furthermore, as mentioned earlier, emerging evidence highlights the role of non-phosphorylated post-translational modifications in Stat3 regulation, which traditional inhibitors overlook by focussing solely on phosphorylation. Additionally, small-molecule inhibitors often require high, repeated dosing, increasing risks of drug resistance^40^ and systemic toxicity. Against this backdrop, PROTAC offers a transformative approach. Unlike small-molecule inhibitors that target phosphorylated Stat3 and neglect complex post-translational regulation, E034 directly induces the degradation of all Stat3 protein forms. This strategy circumvents limitations by leveraging catalytic recycling: after degrading Stat3, E034 is released and reused, enabling sustained target depletion at low doses. By addressing conformational challenges, non-phosphorylated modifications, and dosing drawbacks, E034 provide a more comprehensive means of regulating Stat3 activity in renal injury (Figure S15).

Existing studies have shown that Trim21, an E3 ubiquitin ligase, ubiquitinates GPX4 to promote ferroptosis, exacerbate I/R-induced AKI,^41^ and facilitate ubiquitin-mediated degradation of Stat1.^42^ However, we did not observe that Trim21 promoted ubiquitin-mediated degradation of Stat3 in renal tubular cells during sepsis-induced AKI. Instead, Trim21 directly binds to Gsdmd via its PRY/SPRY domain, promoting N-terminal cleavage and activation of Gsdmd to induce pyroptosis, a process independent of the inflammasome pathway. Although Trim21 did not exhibit ubiquitination of Stat3 in this study, its activation of the NF-κB pathway still relies on the ubiquitination activity of its RING domain.^43^ These findings suggest that the ubiquitination function of Trim21 is substrate- or pathway-specific: in AKI, it may selectively ubiquitinate specific substrates (e.g., NF-κB pathway-related proteins and ferroptosis-related proteins) while exerting no significant effect on others (e.g., Stat3). The dual mechanisms by which Trim21 exacerbates injury in AKI indicate that targeting its specific domains might serve as a potential intervention strategy for AKI, thereby uncovering novel therapeutic approaches for this clinically significant disorder.

While our study identifies H3K27 acetylation as a key epigenetic driver of Stat3 upregulation in AKI, several limitations constrain its translational potential. First, the spatiotemporal dynamics of histone modifications at the Stat3 locus remain undefined, particularly across nephron segments and injury-repair phases; second, other histone marks (e.g., H3K4me3/H3K9me2) and non-coding RNAs that may modulate Stat3 transcription are overlooked. Third, the severe murine AKI models (40-min I/R, CLP) may not reflect epigenetic landscapes in milder clinical cases, and the specificity of pharmacological inhibitors (e.g., C646) for P300/Cbp is uncertain due to potential off-target effects on other histone acetyltransferases. Additionally, the role of non-coding RNAs (e.g., eRNAs/lncRNAs) in recruiting histone modifiers to the Stat3 locus requires further exploration. These gaps highlight the need for integrative epigenomic approaches to characterise Stat3 regulation and fully inform precision therapies. Furthermore, while E034 demonstrates efficacy in pre-clinical models, its safety profile and therapeutic window necessitate rigorous clinical trial evaluation, given the potential variability in pharmacokinetics and pharmacodynamics between murine models and human patients. The complexity of the ubiquitome also implies possible off-target effects of E034, which demand more in-depth investigations in subsequent studies. In addition, exploring effective targeted therapies for Trim21 remains a critical unaddressed area in our research. Additional investigations are essential to assess the efficacy of Trim21-targeted intervention strategies and identify novel treatment modalities for AKI, as this represents a restrictive yet pivotal aspect requiring further clarification.

## Contributors

ML.J designed the study, performed the animal experiments, analysed the data and wrote the manuscript. JN.W designed the study and analysed the data. MF.W performed drug design and synthesis. CH.X performed the cell experiments and verified the underlying data. MM.Z conducted part of the animal experiments. WB.C verified the underlying data and wrote some parts of the manuscript. XF.M performed part of drug design and synthesis. C.L, JT.Y and XG.S performed part of the cellular experiments and histological analysis. DF.Z and NN.M collected human samples. SX.D, Y.C, R.H, H.L, SS.X, YH.D and Q.Z performed part of the cellular experiments and histological analysis. X.C, T.X, W.S, J.J, JG.W and XW.D provided experimental instructions and help. XM.M offered principal guidance throughout this study. All authors have revised and approved the final manuscript.

## Data sharing statement

The data of ChIP-seq and RNA-seq were submitted to GEO database [GEO accessions: GSE283158 (https://www.ncbi.nlm.nih.gov/geo/query/acc.cgi?acc=GSE283158) and GSE283160 (https://www.ncbi.nlm.nih.gov/geo/query/acc.cgi?acc=GSE283160)]. All extended data associated with this study are presented in the Supplementary Materials.

## Declaration of interests

The authors declare that they have no competing interests.

## Appendix A Supplementary data

mTECs STR Identification

Mycoplasma Detection in mTECs

mTECs STR atlas

Source data

HK-2 STR Identification

Supplementary Figures

Supplementary Western blots

Reagent Validation file
