# Supplementary material for: Targeting Stat3 with conditional knockout or PROTAC technology alleviates renal injury by Limiting pyroptosis
Source: eBioMedicine. 2025 May 8;116:105739. doi: 10.1016/j.ebiom.2025.105739 (PMC12136849; doi:10.1016/j.ebiom.2025.105739)
Supplement: mTECs STR Identification [file mmc1.pdf]

Cell Line Authentication Service

STR Profiling Report

Sample From: Anhui Medical University

Sample Type: Cell Line

Testing Method: STR Genotyping

Report Time: March 11, 2025

Add: Room 502, No.2, Zhongxing Creative Park, Lane 1015, Longteng Road, Songjiang District, Shanghai / Tel:+86-18521538068

COMPANY STATEMENT

1. THIS REPORT IS ONLY RESPONSIBLE FOR THE SAMPLES ANALYZED.
2. THE TESTING RESULTS AND THE ORGANIZATION NAME WILL NOT BE USED FOR ADVERTISEMENT, COMMERCIAL EXHIBITIONS, COMMERCIAL PERFORMANCE AND OTHER COMMERCIAL ACTIVITIES.
3. OBJECTIONS SHOULD BE RAISED WITHIN FIFTEEN DAYS AFTER THE RECEIPT OF THIS REPORT.
4. THE PAPER REPORT WITH CONTENT ALTERING, ADDING OR WITHOUT THE STAMPED SEAL OF THE COMPANY ARE INVALID.

Testing Company: Shanghai Biowing Applied Biotechnology Co. Ltd

Address: Room 502, NO.1015 Longteng Rd , Songjiang District, Shanghai

Tel: +86-18521538068

Contact: Shuangning Zhu

E-mail: zhusn@biowing.com.cn

Cell Line Authentication – STR Profiling Report

Sample code

Table 1. Sample Code

Customer's code	Company Code
mTEC	20250306-01

Sample Number:1

Sample Type: Cell line

Testing Type: STR

Testing Method:

DNA was extracted by a commercial kit from CORNING (AP-EMN-BL-GDNA-250G). The ten STRs including one human locus were amplified by multiplex PCR and separated on ABI 3730XL Genetic Analyzer. The signals were then analyzed by the software GeneMapper..

Data Interpretation:

Cell lines were authenticated using Short Tandem Repeat (STR) analysis asdescribed in 2021 in ANSI Standard (ASN-0002) by the ATCC Standards Development Organization (SDO) and in Capes-Davis et al., Match criteria for human cell line authentication: Where do we draw the line? Int J Cancer.2013;132(11):2510-9.

Add: Room 502, No.2, Zhongxing Creative Park, Lane 1015, Longteng Road, Songjiang District, Shanghai / Tel:+86-18521538068

Test Results

1. STR profile

Table 2. STR and Amelogenin Genotyping Results of Cell line.

Loci	Sample information				Cell Bank information		
	Sample name：mTEC				Cell line name：		
	**Allele1**	**Allele2**	**Allele3**	**Allele4**	**Allele1**	**Allele2**	**Allele3**
4-2	229.61 【18.3】						
5-5	335.79 【14】	348.03 【17】					
6-4	300.01 【18】						
6-7	334.59 【12】	346.63 【15】					
9-2	225.24 【16】						
12-1	229.86 【17】						
15-3	192.78 【20.3】						
18-3	152.13 【16】	160.28 【18】					
X-1	404.31 【26】						
D4S2408							

2. database annotation

Figure 1. STR matching analysis

Accession	Name	N° Markers	Score	STR 1-1	STR 1-2	STR 2-1	STR 3-2	STR 4-2	STR 5-5	STR 6-4	STR 6-7	STR 7-1	STR 8-1	STR 11-2	STR 12-1	STR 13-1	STR 15-3	STR 17-2	STR 18-3	STR 19-2	STR X-1
NA	Query	NA	NA					18.3	14,17	18	12,15				17		20.3		16,18		26
CVCL_ZL61	IngWAT	8	66.67%	10,16,17	13,19	9	14	21.3	14,18	17,18	12,15	27.2	15.3	14,16	17	17	20.3,22.3	12,16	16,18	13	27
CVCL_VU16	WT-1	8	66.67%	10	13	9	14	19.3,20.3	17	17	12	29	15.3	14	17	15.2,16.2	20.3	12,15	16,18	12,13	26

Note: The STR online match analysis of the test cell against EXPASY database, showing cell number (Cell No.) and cell name.

Add: Room 502, No.2, Zhongxing Creative Park, Lane 1015, Longteng Road, Songjiang District, Shanghai / Tel:+86-18521538068

3. Authentication

☑ The submitted sample profile is mouse, but not a match for any profile in the EXPASY. As the STR database of **mTEC** was not logged in, the matching result cannot be displayed. The cell line is unique and not cross-contaminated or misidentified, if the paper was to publish, these data could be submitted to the magazine.

☐ The submitted profile is an exact match for the following human cell line(s) in the DSMZ STR database (8 core loci plus Amelogenin): .

☐ The submitted profile is similar to the following DSMZ human cell line: /.

- **Note:** Cell lines are considered to related, derived from a common ancestry, when ≥80% of the alleles in its STR profile match profiles from tissue or other cell line samples from that donor or from database. Cell lines with between a 55% to 80% (similar) match require further profiling for investigation of relatedness.

Figure 2. STR profiles of sample cell line

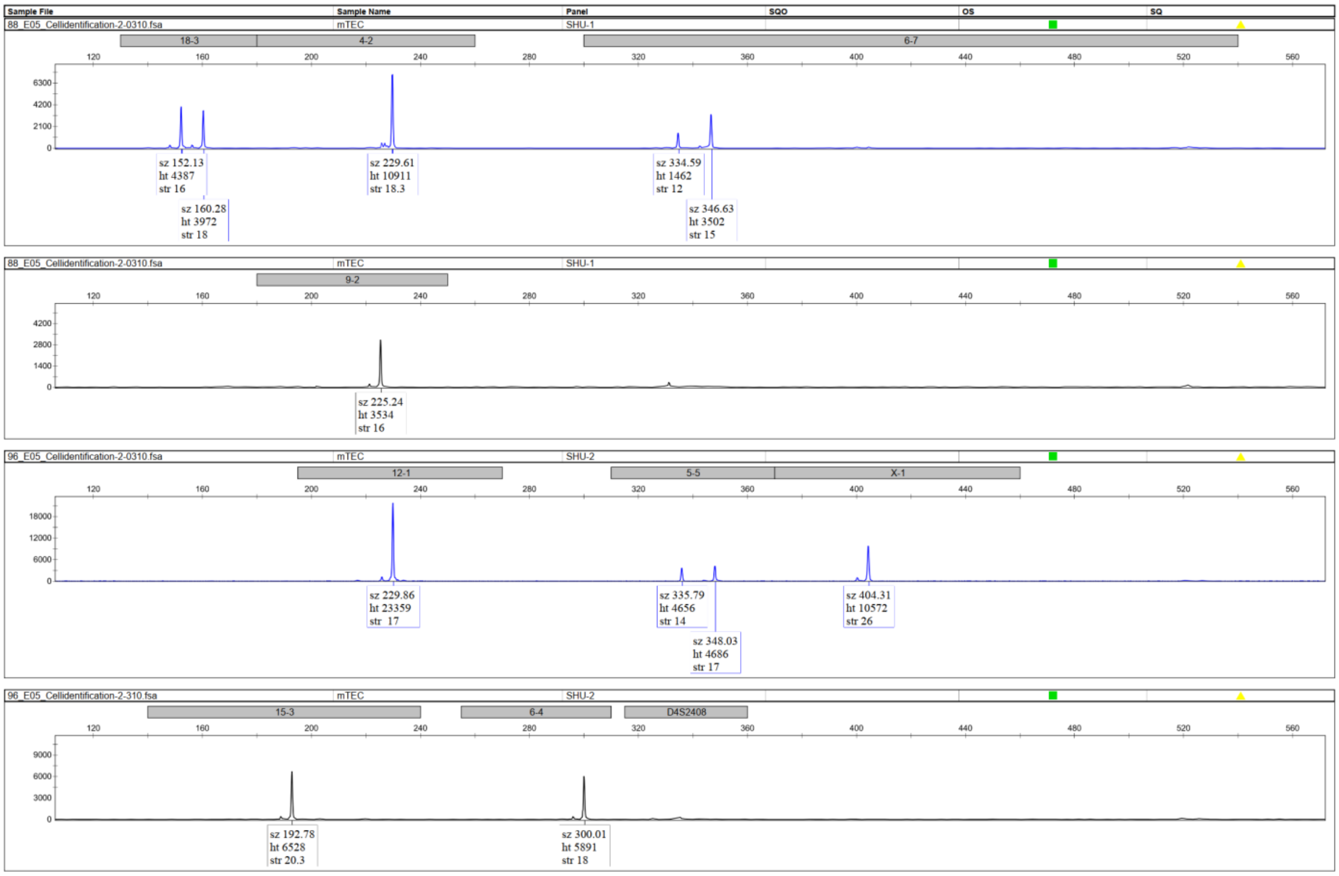

Add: Room 502, No.2, Zhongxing Creative Park, Lane 1015, Longteng Road, Songjiang District, Shanghai / Tel:+86-18521538068

Appendix:

1. Genotyping Strategy and Site Distribution

Table S1. Experimental Strategy and Sites

	Strategy 1	Strategy 2
1	18-3(FAM)	12-1(FAM)
2	4-2 (FAM)	5-5(FAM)
3	6-7(FAM)	X-1(FAM)
4	9-2(NED)	15-3(NED)
5		6-4(NED)
6		D4S2408(NED)

The allele match algorithm compares the 9 core loci only, D4S2408 is a human site, which is used to detect whether the cell is contaminated by human sources.

2. STR tools was used to carry on the cell line comparison, which contains 8000 cell lines STR data from ATCC, DSMZ, JCRB ,ECACC， GNE and RIKEN databases. If the cell is not included in the above cell library, users need to compared with other databases. D4S2408 is a human locus, which is used to detect whether the cells are contaminated by human.

Shanghai Biowing Applied Biotechnology Co. Ltd * STR Profiling Cell Line Authentication

Technician: Xiuchuan He

Checked by: Chenqian Zhang

Issued by: Min Wang

Issue date: March 11, 2025

Add: Room 502, No.2, Zhongxing Creative Park, Lane 1015, Longteng Road, Songjiang District, Shanghai / Tel:+86-18521538068
