# Supplementary material for: Targeting Stat3 with conditional knockout or PROTAC technology alleviates renal injury by Limiting pyroptosis
Source: eBioMedicine. 2025 May 8;116:105739. doi: 10.1016/j.ebiom.2025.105739 (PMC12136849; doi:10.1016/j.ebiom.2025.105739)
Supplement: Mycoplasma Detection in mTECs [file mmc2.pdf]

Mycoplasma Test Report

Information introduction

Company Code:	20250306_5
Sample Code:	mTEC
Sample From: Sample Type:	Anhui Medical University
Testing Type:	Detection of Mycoplasma
Testing Method:	TaqMan-qPCR

Testing Method

1. Based on the fluorescent quantitative PCR platform, the mycoplasma DNA can be quantitatively detected by the FAM probe in a single reaction。
2. The internal standard is introduced in the multiplex PCR process, the VIC probe is used to detect the internal standard gene to monitor the experimental process to avoid false negatives。
3. The QPCR amplification reaction is in a closed environment throughout the entire process, which effectively avoids false positive results caused by PCR product contamination, and at the same time uses negative reference products to control false positive。
4. Quantify the mycoplasma DNA in the sample by the positive reference concentration standard curve to obtain the concentration of mycoplasma DNA in the sample and give the reference degree of mycoplasma contamination。
5. This method is highly sensitive and can detect as few as 10 copies of mycoplasma DNA。

Quality Control

1. The positive detection channel（FAM）and internal standard channel (HEX) shows an "S" type amplification curve, and the Ct value of each reference product has a good linearity，R^2=0.9998。

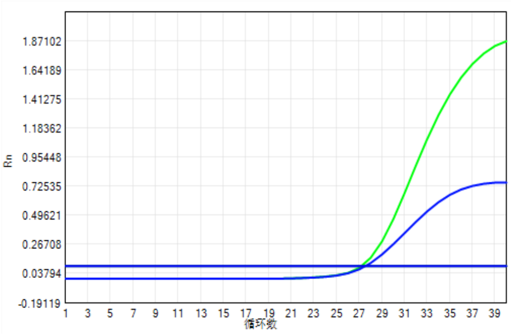

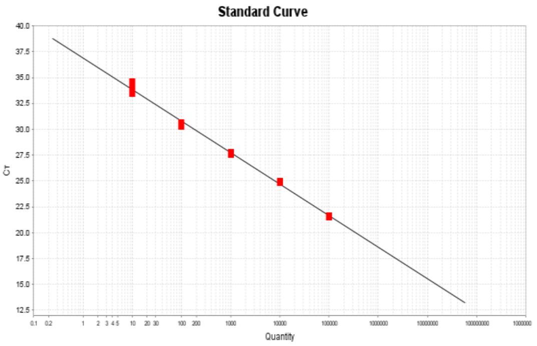

2. The negative reference detection channel (blue FAM) has no amplification performance, the internal standard channel (green VIC) shows a standard "S" type amplification curve, and the negative control is established。

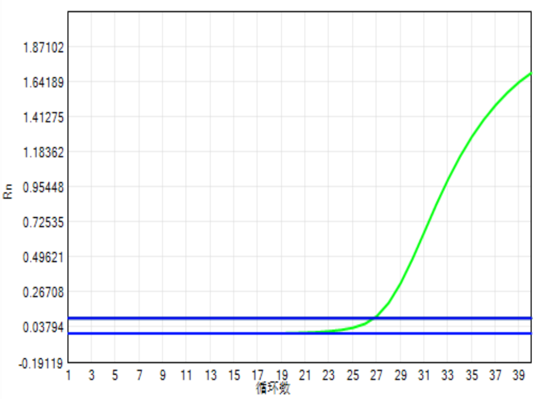

Test Results

1. Sample detection channel amplification curve (left FAM), internal standard channel amplification curve (right VIC). The internal standard channel shows an "S" type amplification curve, indicating that the PCR process of the sample is normal。

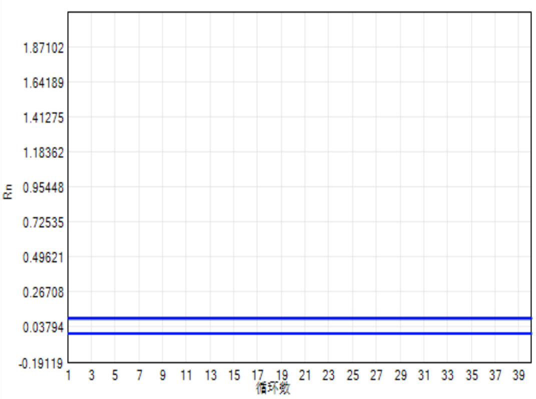

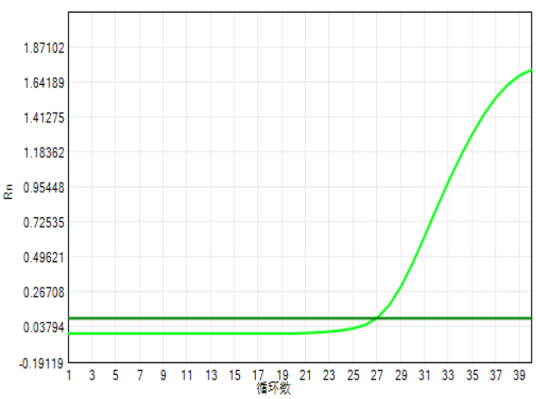

2、Qualitative standards

Detection channel Ct range(FAM)	Internal reference channel Ct range(HEX/VIC)	Qualitative results
Ct<19	25<Ct<29	Strong positive(+++)
19≤Ct<25	25<Ct<29	Positive (++)
25≤Ct<30	25<Ct<29	Weak positive(+)
Ct>33or NoCt	25<Ct<29	Negative (-)
30<CT≤33	25<Ct<29	Need to revalidate

3、Summary of test results

Inspection Channel	Detection channel Ct range(FAM)	Internal reference channel Ct range(VIC)	Qualitative results	Reference concentration
Positive	Ct∈(23-29)	27.18	-	-
Negative	NoCt	26.75	-	-
sample	NoCt	26.92	Negative	-

Conclusion

1、The amplification result of the positive reference product was normal, and the Ct value between each reference product was linear. No amplification in the detection channel of the negative reference product, and the internal standard gene amplification was nomal. No abnormality in the experimental process。

2、The internal reference gene amplification of the tested sample is normal, the Ct value is 26.92, and the PCR process of the tested product is normal。

3、The Ct value of the sample is NoCt, so the mycoplasma test result of the sample is Negative.

Biowing Applied Biotechnology Co. Ltd Shanghai
Cell Line Test of Mycoplasma

Technician: Chenqian Zhang

Checked by: Kaiyue Chao

Issued by: Shuangning Zhu

Issue date: March 6, 2025
