# Supplementary figures and images for: Targeting Stat3 with conditional knockout or PROTAC technology alleviates renal injury by Limiting pyroptosis
Source: eBioMedicine. 2025 May 8;116:105739. doi: 10.1016/j.ebiom.2025.105739 (PMC12136849; doi:10.1016/j.ebiom.2025.105739)

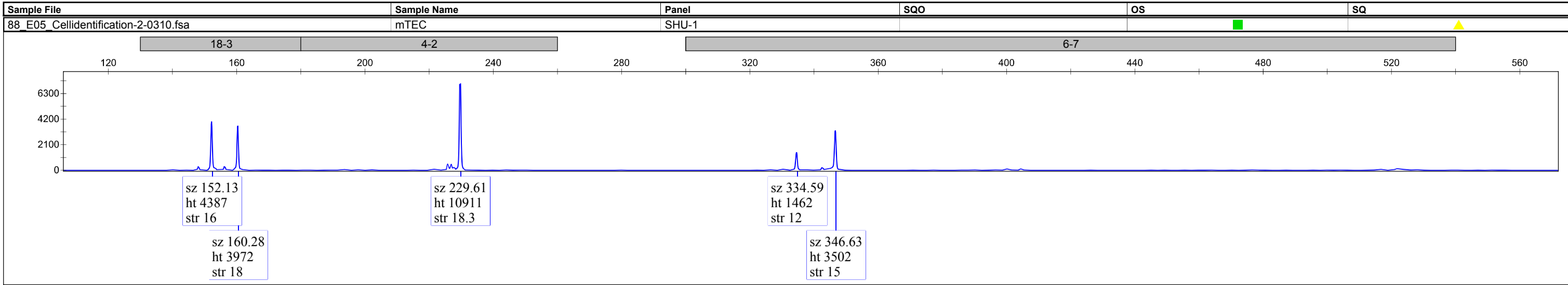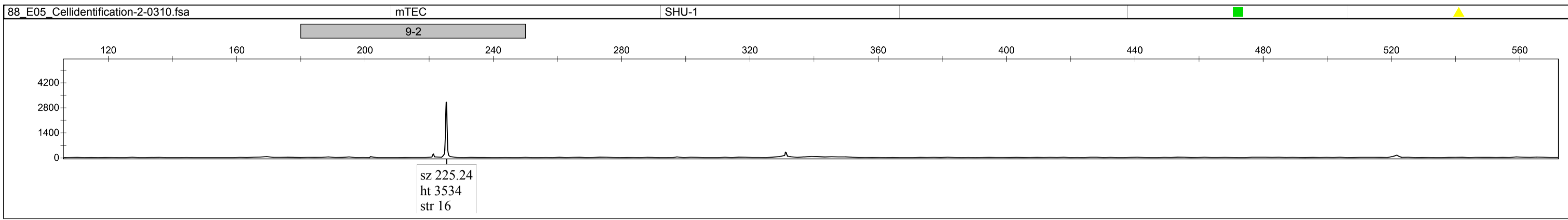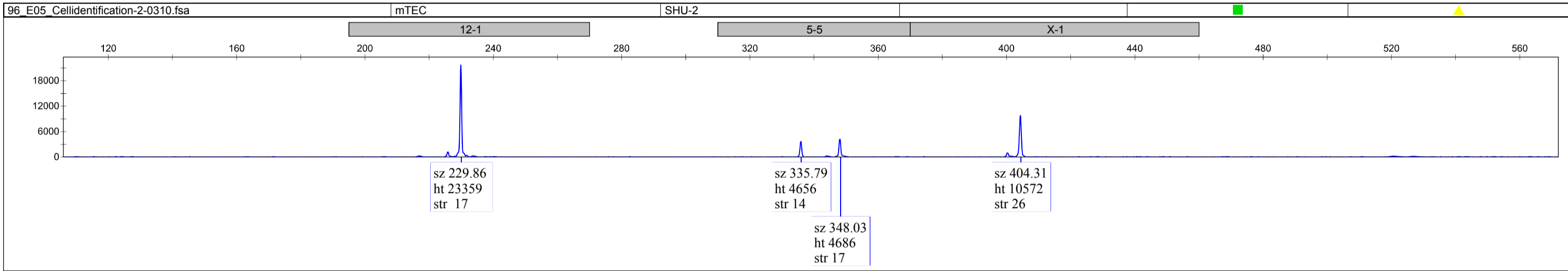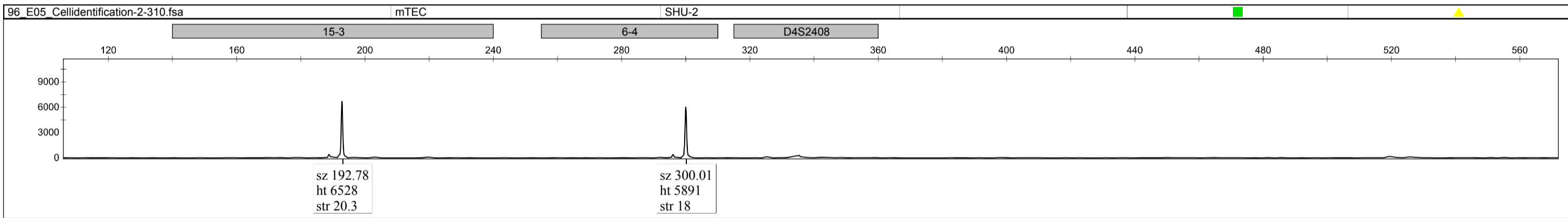

Supplement: mTECs STR atlas [file mmc3.pdf]
