# Supplementary material for: Targeting Stat3 with conditional knockout or PROTAC technology alleviates renal injury by Limiting pyroptosis
Source: eBioMedicine. 2025 May 8;116:105739. doi: 10.1016/j.ebiom.2025.105739 (PMC12136849; doi:10.1016/j.ebiom.2025.105739)
Supplement: HK-2 STR Identification [file mmc5.pdf]

HK-2细胞STR鉴定报告

一、 材料处理和检验方法

取适量HK-2细胞(编号PC-H2025022708,1×10^6)使用TIANamp Genomic DNA Kit提取DNA，采用Microreader™21 ID System扩增20个STR位点和性别鉴定位点，使用智阅基因分析仪GenReader 7010对PCR产物进行检测，使用GeneMapper Software6软件（Applied Biosystems）对检测结果进行分析，并与ExPASy数据库进行比对。

二、 检测结果

实验中阴性及阳性对照结果均正确。

HK-2 细胞株的 STR 位点和 Amelogenin 位点的基因分型结果见附表，分型图谱见附图。

三、 分析说明

HK-2 细胞株基因组 DNA 扩增后图谱清晰，分型结果良好。

四、 检验结论

1. HK-2 细胞株 DNA 进行细胞 STR 分型结果显示，细胞株中未发现人类细胞交叉污染。
2. 该细胞株 DNA 分型在细胞库中找到与其细胞分型 94.55%相匹配的细胞株，细胞株名称为 HK-2 [Human kidney]。

普诺赛® Procell | Pricella

网站：www.procell.com.cn
电话：400-999-2100
邮箱：techsupport@procell.com.cn
地址：湖北省武汉市高新大道858号生物医药产业园三期C4栋

附表1：HK-2细胞株的STR位点和Amelogenin位点的基因分型结果。

STR Loci	样品名称：PC-H2025022708	数据库名称：HK-2 [Human kidney]
Amelogenin	X	X
CSF1PO	13	13
D2S1338	17,25	17,25
D3S1358	16	16,17
D5S818	12	12
D7S820	10,11	10,11
D8S1179	10,14	10,14
D13S317	9	9
D16S539	12	11,12
D18S51	12	12
D19S433	15,15.2	15,15.2
D21S11	28,30	28,30
FGA	20	20,22
Penta D	9,12	9,12
Penta E	10,11	10,11
TH01	9	9
TPOX	8,9	8,9
vWA	17,18	17,18
D6S1043	12,13	
D12S391	17.3,22	
D2S441	11,12	
ExPASy数据库匹配度94.55%，匹配位点数17（https://www.cellosaurus.org/index.html）		

备注：

1. 根据国际细胞鉴定委员会(ICLAC)制定的细胞 STR 鉴定标准，细胞系的匹配度≥80%时，认为它们具有相关性，即衍生于共同的祖先细胞；匹配度在 55% 至 80% 之间，需要进一步验证相关性；小于 55%，表明两者不具有相关性。
2. 图谱有效峰为真实的 PCR 条带;小峰和非特异性条带在计算中忽略不计。
3. STR 数据比对结果默认 ExPASy，数据来源包括 ATCC，DSMZ，JCRB 等细胞库以及文献和资料记载，数据库入口 https://www.cellosaurus.org/index.html。

网站：www.procell.com.cn
电话：400-999-2100
邮箱：techsupport@procell.com.cn
地址：湖北省武汉市高新大道858号生物医药产业园三期C4栋

普诺赛® Procell | Pricella 武汉普诺赛生命科技有限公司 Wuhan Pricella Biotechnology Co., Ltd.

附图1：HK-2（编号PC-H2025022708）细胞STR位点和Amelogenin位点的基因分型结果。

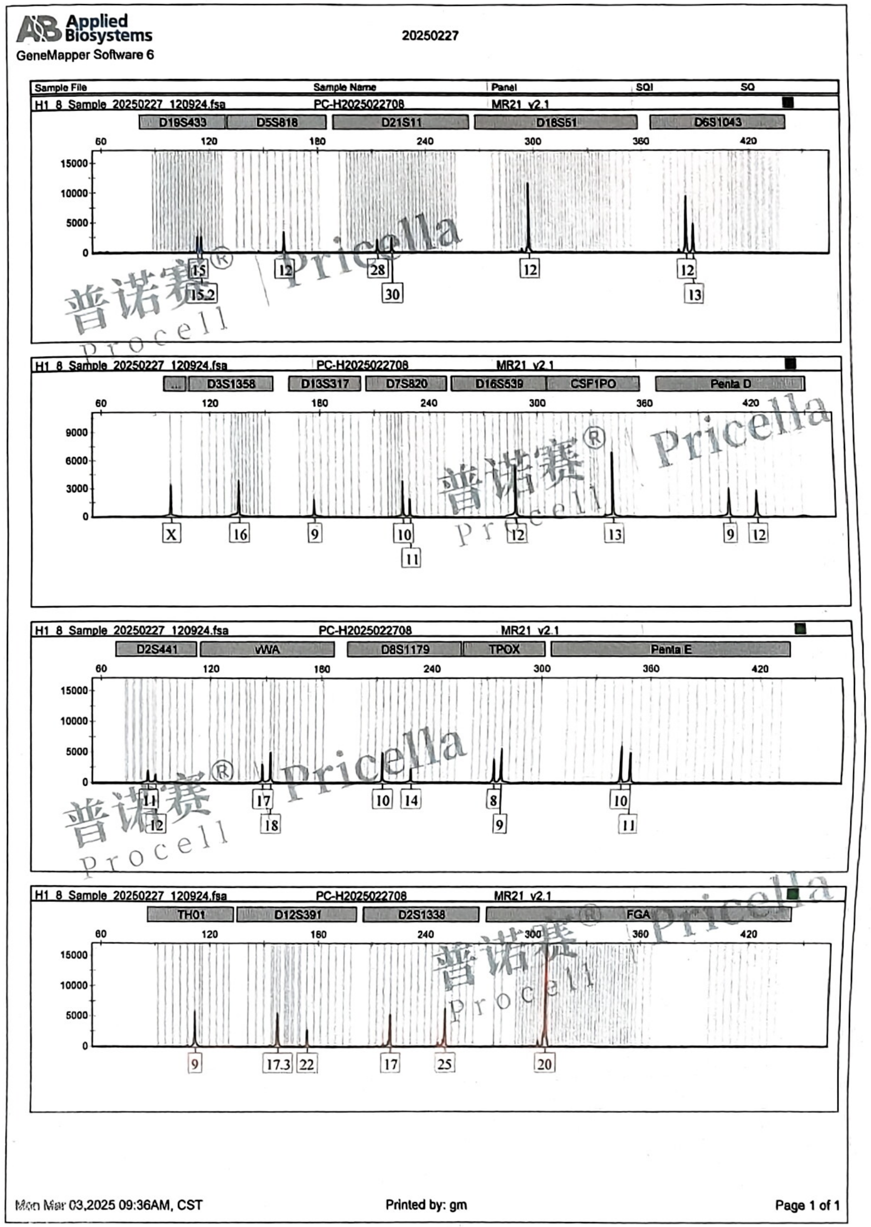

网站：www.procell.com.cn
电话：400-999-2100
邮箱：techsupport@procell.com.cn
地址：湖北省武汉市高新大道858号生物医药产业园三期C4栋

普诺赛® Procell | Pricella

武汉普诺赛生命科技有限公司
Wuhan Pricella Biotechnology Co., Ltd.

Certificate of Analysis

HK-2

人肾皮质近曲小管上皮细胞

货号：CL-0109　　报告批准日期：2025-04-07

规格：1×10^6Cells/T25　　保存温度：常温

Quality Profile

检测项目	单位	标准	结果
外观	/	标签清晰正确，瓶身外观完好，瓶盖盖紧	合格
数量	cell	≥1×10^6	合格
汇合度	/	≥70%	合格
细胞形态	/	上皮细胞样	合格
细菌	/	镜检无污染	合格
真菌	/	镜检无污染	合格
支原体	/	PCR法/显色法阴性	合格

质检结论：

参考《HK-2细胞产品技术要求》，判定该产品**合格!**

武汉普诺赛生命科技有限公司 质检专用章

报告人：李秀

批准人：熊薇

2025-04-09

网站: www.procell.com.cn
电话: 400-999-2100
邮箱: techsupport@procell.com.cn
地址: 湖北省武汉市高新大道858号生物医药产业园三期C4栋
