# Supplementary material for: Targeting Stat3 with conditional knockout or PROTAC technology alleviates renal injury by Limiting pyroptosis
Source: eBioMedicine. 2025 May 8;116:105739. doi: 10.1016/j.ebiom.2025.105739 (PMC12136849; doi:10.1016/j.ebiom.2025.105739)
Supplement: Supplementary Figures [file mmc6.pdf]

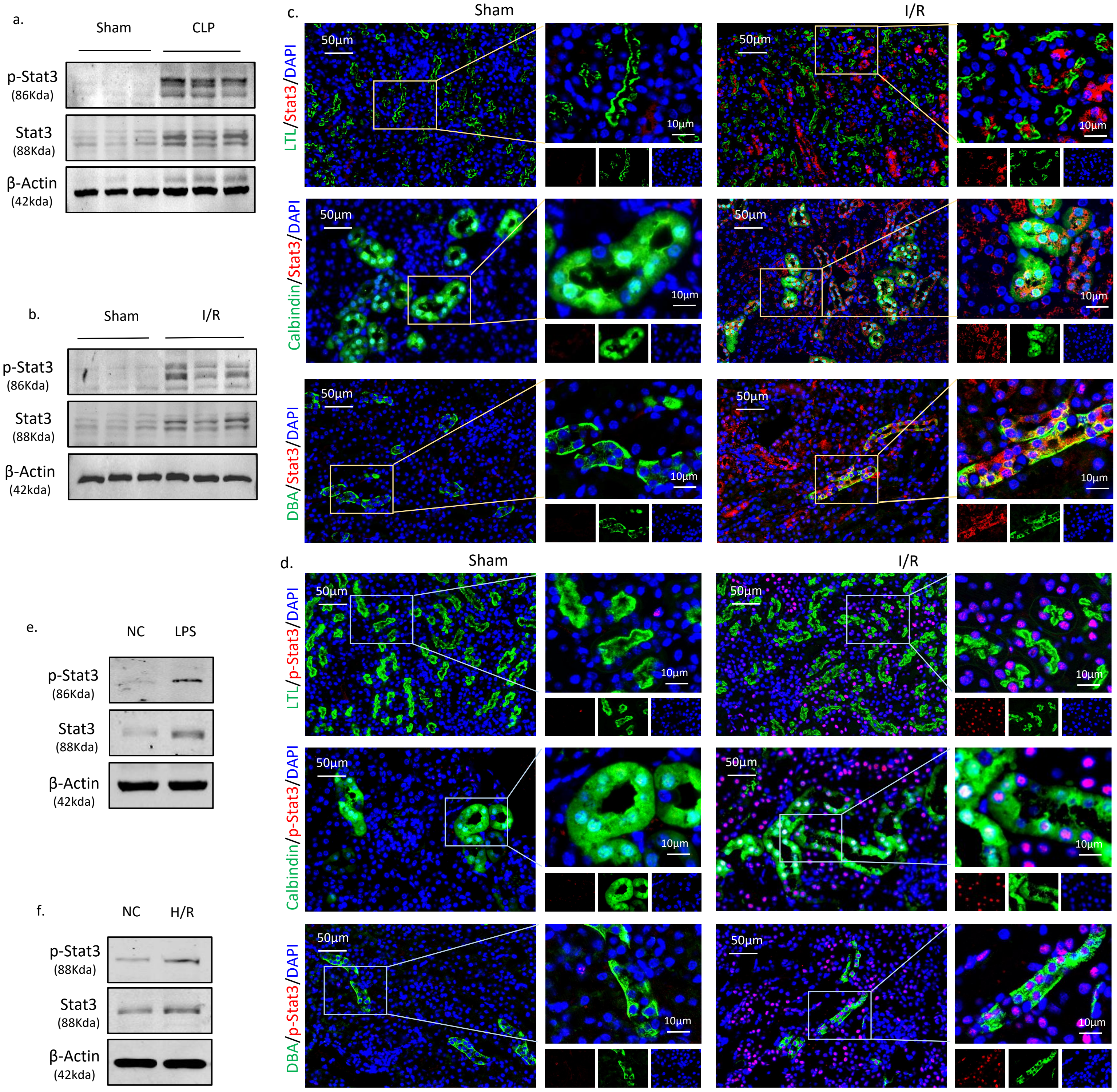

a.
Sham
CLP
p-Stat3 (86Kda)
Stat3 (88Kda)
β-Actin (42kda)
b.
Sham
I/R
p-Stat3 (86Kda)
Stat3 (88Kda)
β-Actin (42kda)
e.
NC
LPS
p-Stat3 (86Kda)
Stat3 (88Kda)
β-Actin (42kda)
f.
NC
H/R
p-Stat3 (88Kda)
Stat3 (88Kda)
β-Actin (42kda)
c.
Sham
I/R
LTL/Stat3/DAPI
Calbindin/Stat3/DAPI
DBA/Stat3/DAPI
50μm
10μm
d.
Sham
I/R
LTL/p-Stat3/DAPI
Calbindin/p-Stat3/DAPI
DBA/p-Stat3/DAPI
50μm
10μm

Figure S1

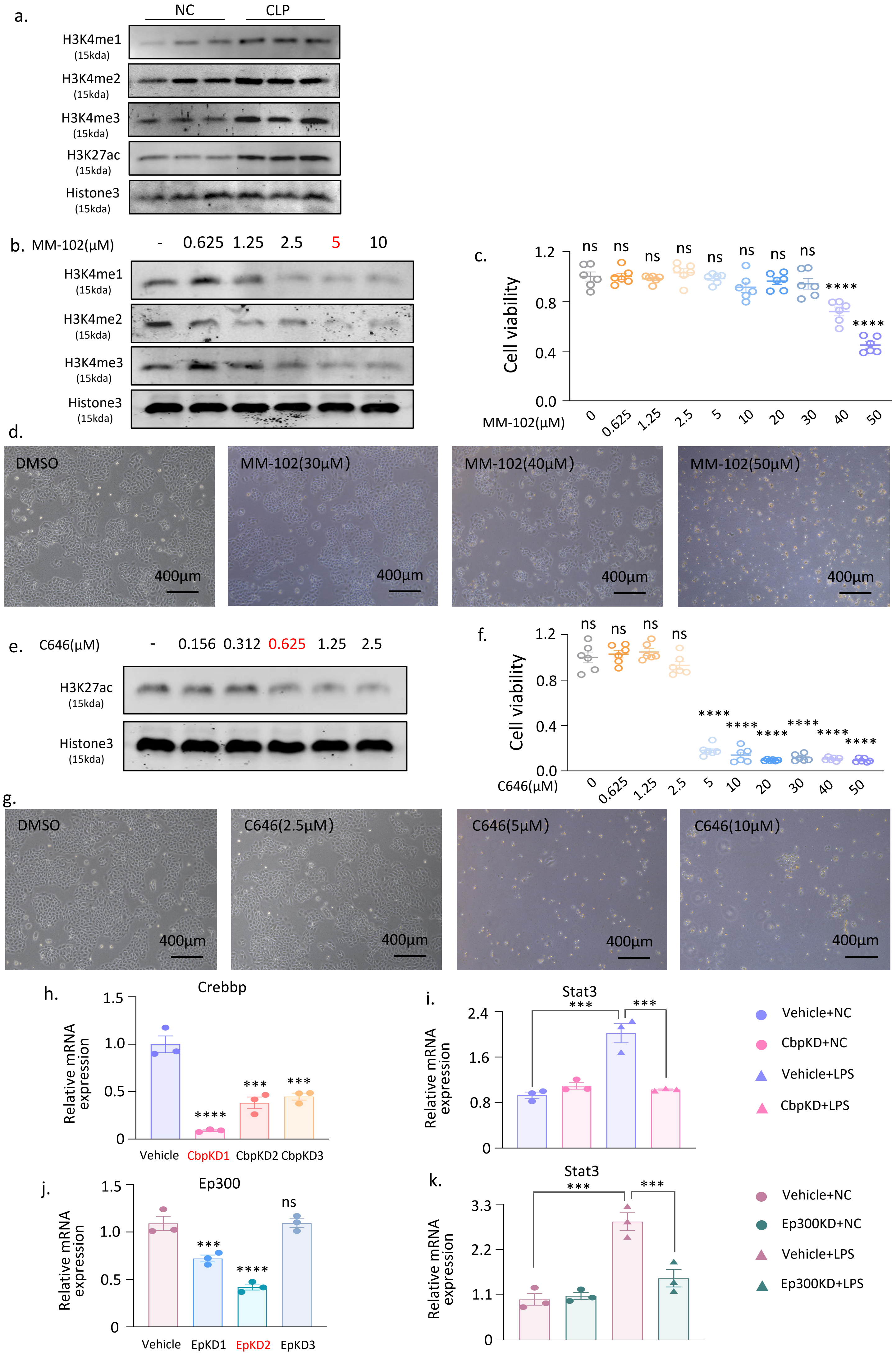
a.
NC
CLP
H3K4me1 (15kda)
H3K4me2 (15kda)
H3K4me3 (15kda)
H3K27ac (15kda)
Histone3 (15kda)
b. MM-102(μM) - 0.625 1.25 2.5 5 10
H3K4me1 (15kda)
H3K4me2 (15kda)
H3K4me3 (15kda)
Histone3 (15kda)
c.
Cell viability
1.2 0.8 0.4 0.0
ns ns ns ns ns ns ns ns **** ****
MM-102(μM) 0 0.625 1.25 2.5 5 10 20 30 40 50
d.
DMSO
MM-102(30μM)
MM-102(40μM)
MM-102(50μM)
400μm
e. C646(μM) - 0.156 0.312 0.625 1.25 2.5
H3K27ac (15kda)
Histone3 (15kda)
f.
Cell viability
1.2 0.8 0.4 0.0
ns ns ns ns **** **** **** **** **** ****
C646(μM) 0 0.625 1.25 2.5 5 10 20 30 40 50
g.
DMSO
C646(2.5μM)
C646(5μM)
C646(10μM)
400μm
h.
Crebbp
Relative mRNA expression
1.5 1.0 0.5 0
**** *** ***
Vehicle CbpKD1 CbpKD2 CbpKD3
i.
Stat3
Relative mRNA expression
2.4 1.6 0.8 0
*** ***
Vehicle+NC
CbpKD+NC
Vehicle+LPS
CbpKD+LPS
j.
Ep300
Relative mRNA expression
1.5 1.0 0.5 0
*** **** ns
Vehicle EpKD1 EpKD2 EpKD3
k.
Stat3
Relative mRNA expression
3.3 2.2 1.1 0
*** ***
Vehicle+NC
Ep300KD+NC
Vehicle+LPS
Ep300KD+LPS

Figure S2

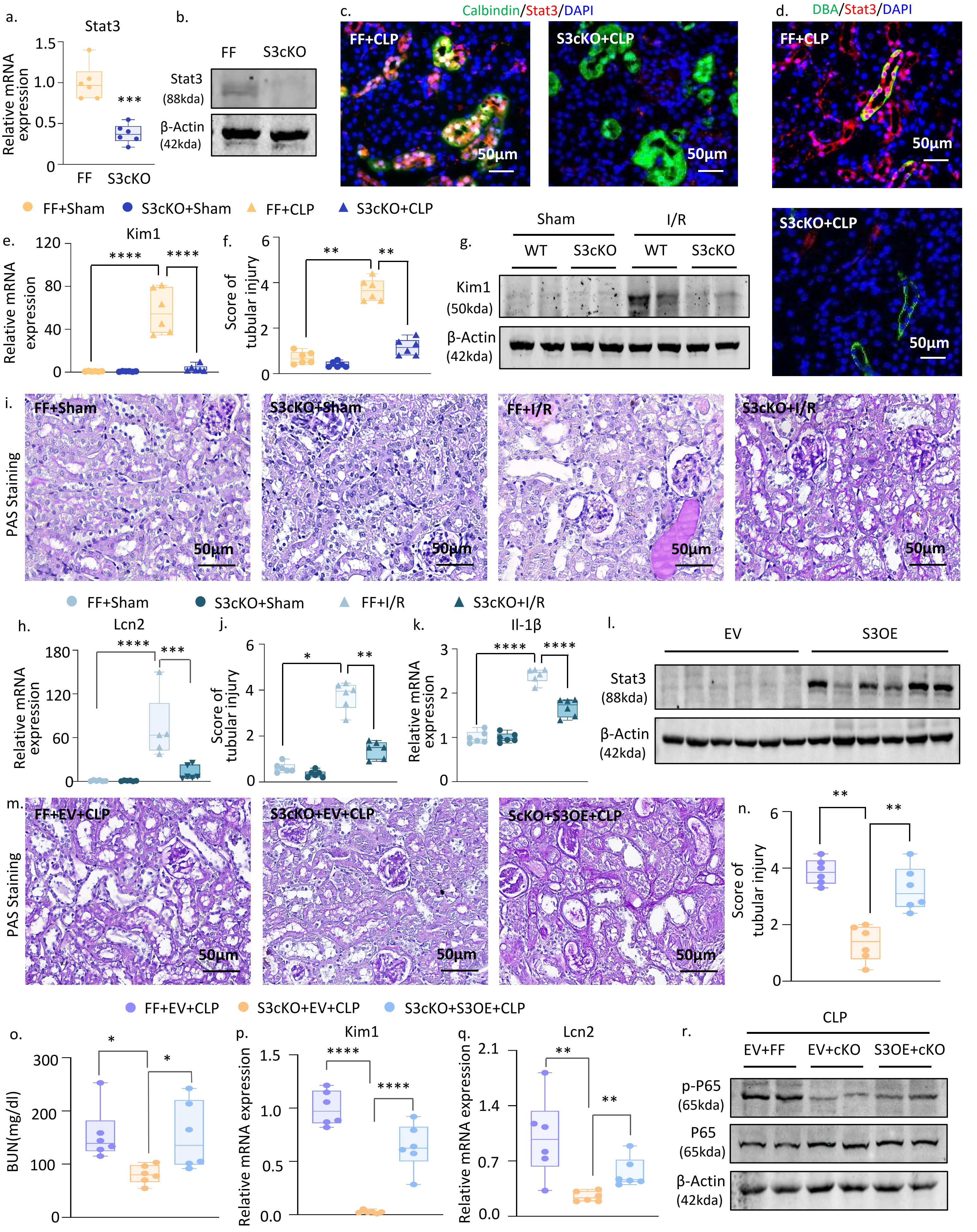
a.
Stat3
Relative mRNA expression
FF
S3cKO
***
b.
FF
S3cKO
Stat3 (88kda)
β-Actin (42kda)
c.
Calbindin/Stat3/DAPI
FF+CLP
S3cKO+CLP
50μm
d.
DBA/Stat3/DAPI
FF+CLP
S3cKO+CLP
FF+Sham
S3cKO+Sham
FF+CLP
S3cKO+CLP
e.
Kim1
Relative mRNA expression
f.
Score of tubular injury
g.
Sham
I/R
WT
S3cKO
Kim1 (50kda)
β-Actin (42kda)
i.
PAS Staining
FF+Sham
S3cKO+Sham
FF+I/R
S3cKO+I/R
FF+Sham
S3cKO+Sham
FF+I/R
S3cKO+I/R
h.
Lcn2
j.
k.
Il-1β
l.
EV
S3OE
Stat3 (88kda)
β-Actin (42kda)
m.
FF+EV+CLP
S3cKO+EV+CLP
ScKO+S3OE+CLP
n.
FF+EV+CLP
S3cKO+EV+CLP
S3cKO+S3OE+CLP
o.
BUN(mg/dl)
p.
Kim1
q.
Lcn2
r.
CLP
EV+FF
EV+cKO
S3OE+cKO
p-P65 (65kda)
P65 (65kda)
β-Actin (42kda)

Figure S3

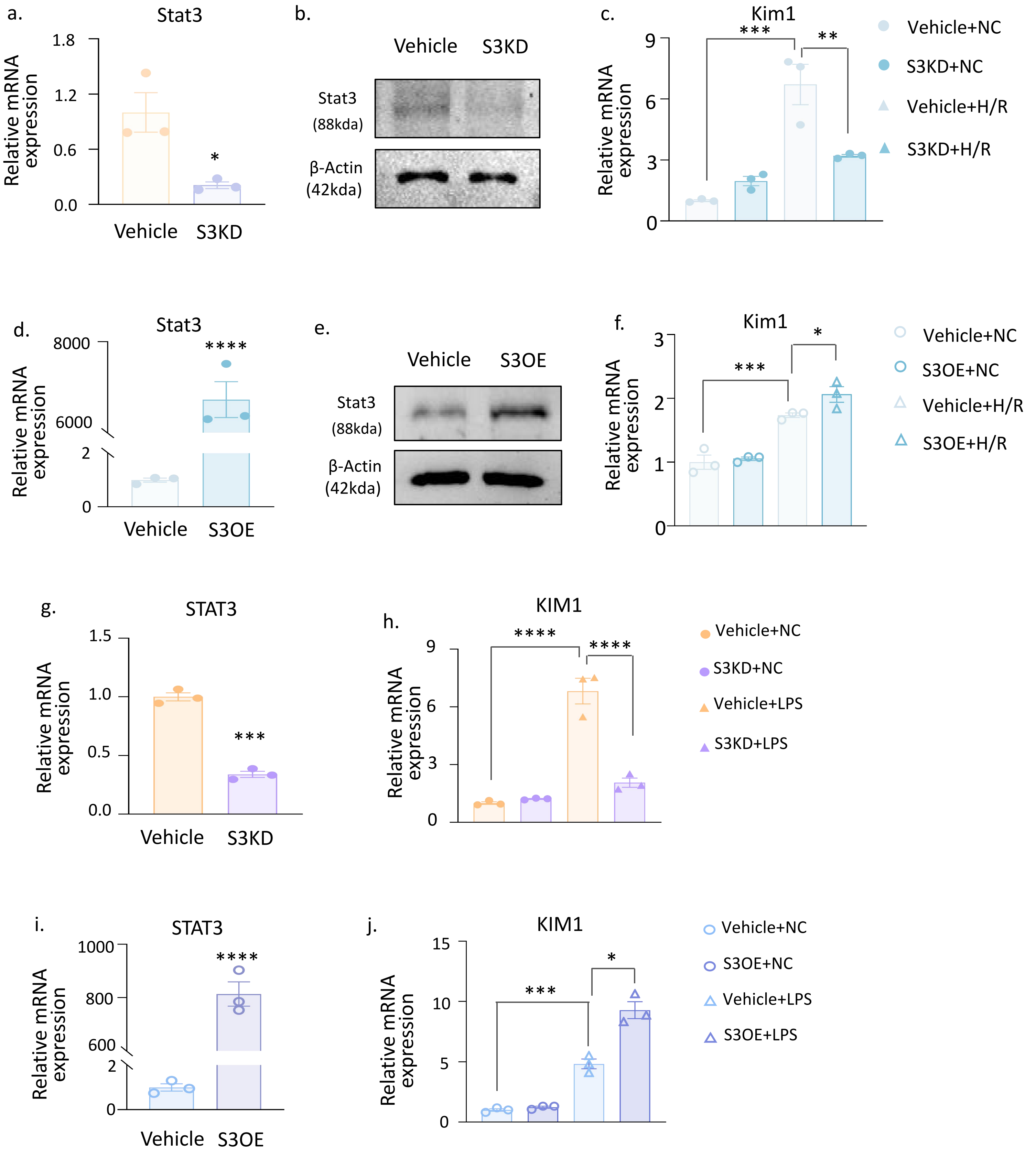

a.
Stat3
Relative mRNA expression
1.8
1.2
0.6
0.0
*
Vehicle
S3KD
b.
Vehicle
S3KD
Stat3
(88kda)
β-Actin
(42kda)
c.
Kim1
***
**
Relative mRNA expression
9
6
3
0
Vehicle+NC
S3KD+NC
Vehicle+H/R
S3KD+H/R
d.
Stat3
8000
6000
2
0
****
Relative mRNA expression
Vehicle
S3OE
e.
Vehicle
S3OE
Stat3
(88kda)
β-Actin
(42kda)
f.
Kim1
*
***
3
2
1
0
Relative mRNA expression
Vehicle+NC
S3OE+NC
Vehicle+H/R
S3OE+H/R
g.
STAT3
1.5
1.0
0.5
0.0
***
Relative mRNA expression
Vehicle
S3KD
h.
KIM1
****
****
9
6
3
0
Relative mRNA expression
Vehicle+NC
S3KD+NC
Vehicle+LPS
S3KD+LPS
i.
STAT3
1000
800
600
2
0
****
Relative mRNA expression
Vehicle
S3OE
j.
KIM1
15
10
5
0
*
***
Relative mRNA expression
Vehicle+NC
S3OE+NC
Vehicle+LPS
S3OE+LPS

Figure S4

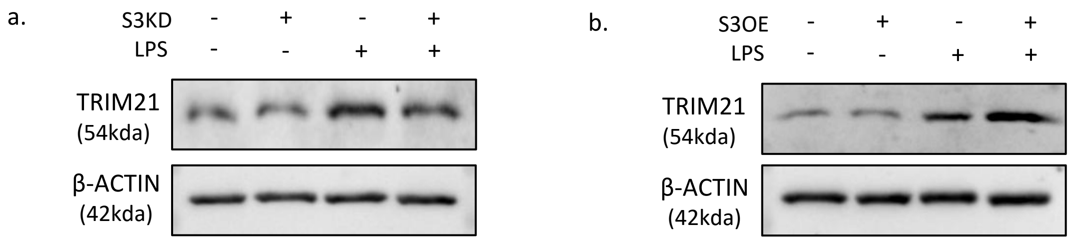
a.
S3KD - + - +
LPS - - + +
TRIM21
(54kda)
β-ACTIN
(42kda)
b.
S3OE - + - +
LPS - - + +
TRIM21
(54kda)
β-ACTIN
(42kda)

Figure S5

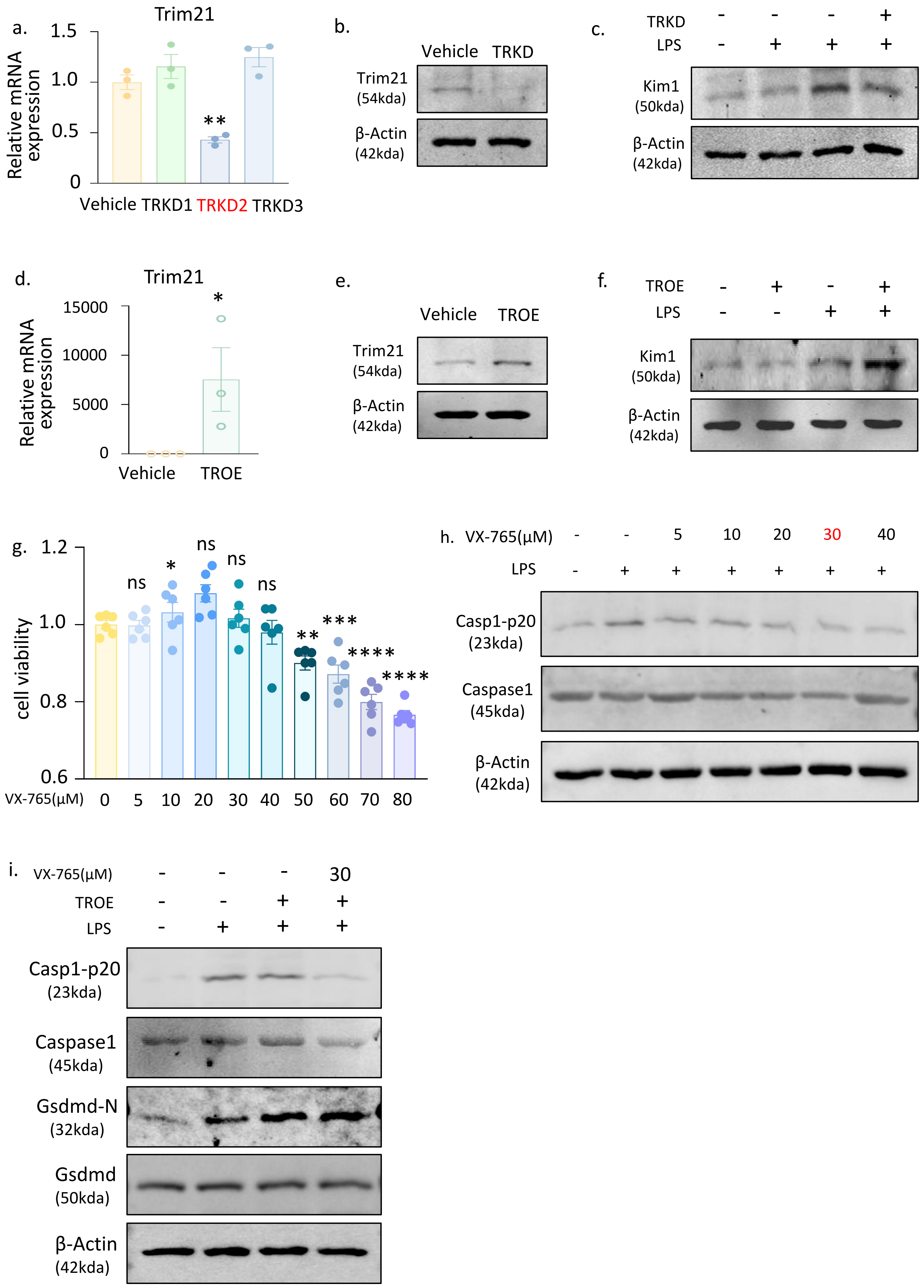
a.
Trim21
Relative mRNA expression
1.5
1.0
0.5
0
**
Vehicle TRKD1 TRKD2 TRKD3
b.
Vehicle TRKD
Trim21 (54kda)
β-Actin (42kda)
c.
TRKD - - - +
LPS - + + +
Kim1 (50kda)
β-Actin (42kda)
d.
Trim21
Relative mRNA expression
15000
10000
5000
0
*
Vehicle TROE
e.
Vehicle TROE
Trim21 (54kda)
β-Actin (42kda)
f.
TROE - + - +
LPS - - + +
Kim1 (50kda)
β-Actin (42kda)
g.
cell viability
1.2
1.0
0.8
0.6
ns * ns ns ns ** *** **** ****
VX-765(μM) 0 5 10 20 30 40 50 60 70 80
h.
VX-765(μM) - - 5 10 20 30 40
LPS - + + + + + +
Casp1-p20 (23kda)
Caspase1 (45kda)
β-Actin (42kda)
i.
VX-765(μM) - - - 30
TROE - - + +
LPS - + + +
Casp1-p20 (23kda)
Caspase1 (45kda)
Gsdmd-N (32kda)
Gsdmd (50kda)
β-Actin (42kda)

Figure S6

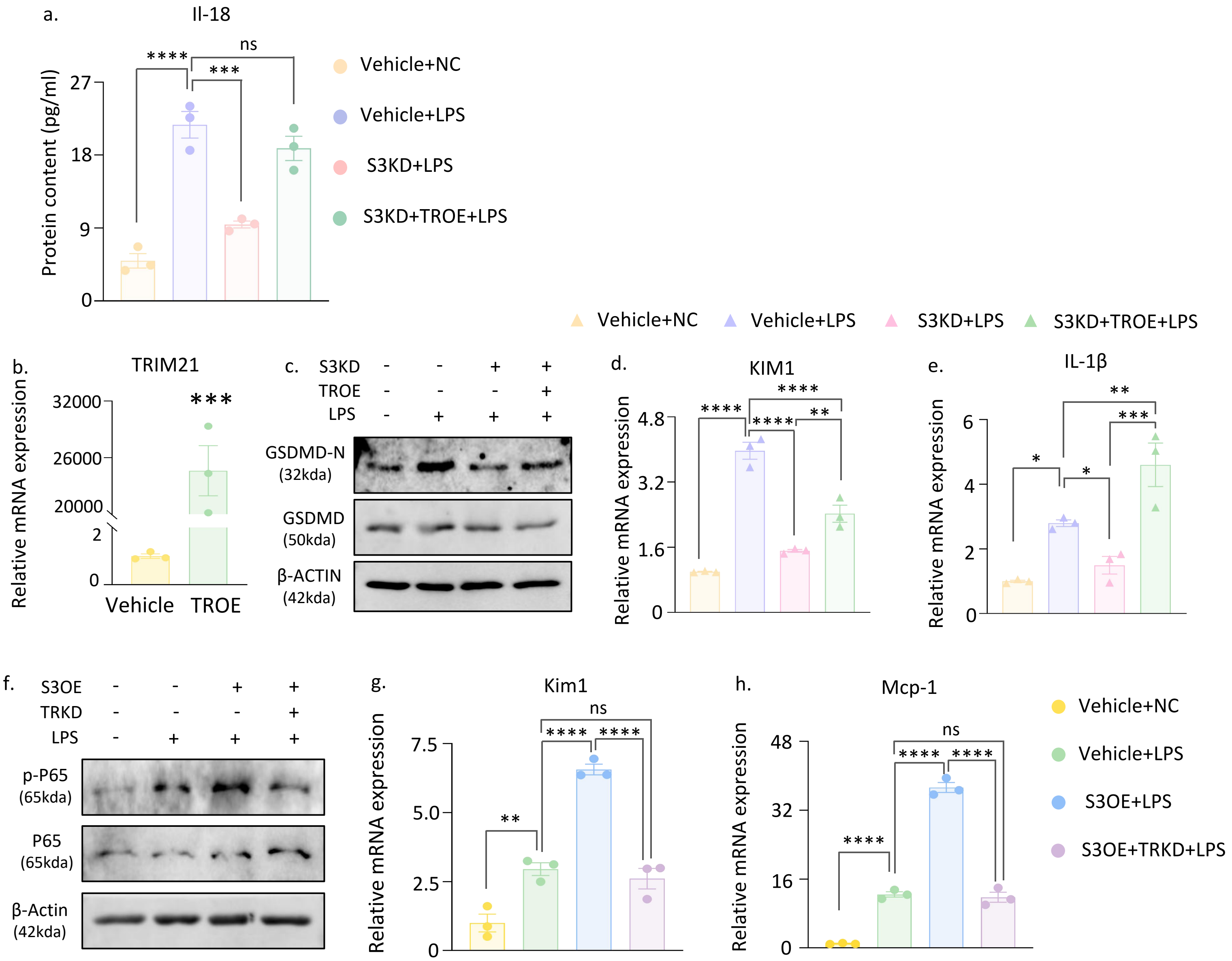
a.
Il-18
Protein content (pg/ml)
Vehicle+NC
Vehicle+LPS
S3KD+LPS
S3KD+TROE+LPS
b.
TRIM21
Relative mRNA expression
Vehicle
TROE
c.
S3KD
TROE
LPS
GSDMD-N (32kda)
GSDMD (50kda)
β-ACTIN (42kda)
d.
KIM1
e.
IL-1β
f.
S3OE
TRKD
LPS
p-P65 (65kda)
P65 (65kda)
β-Actin (42kda)
g.
Kim1
h.
Mcp-1
S3OE+LPS
S3OE+TRKD+LPS

Figure S7

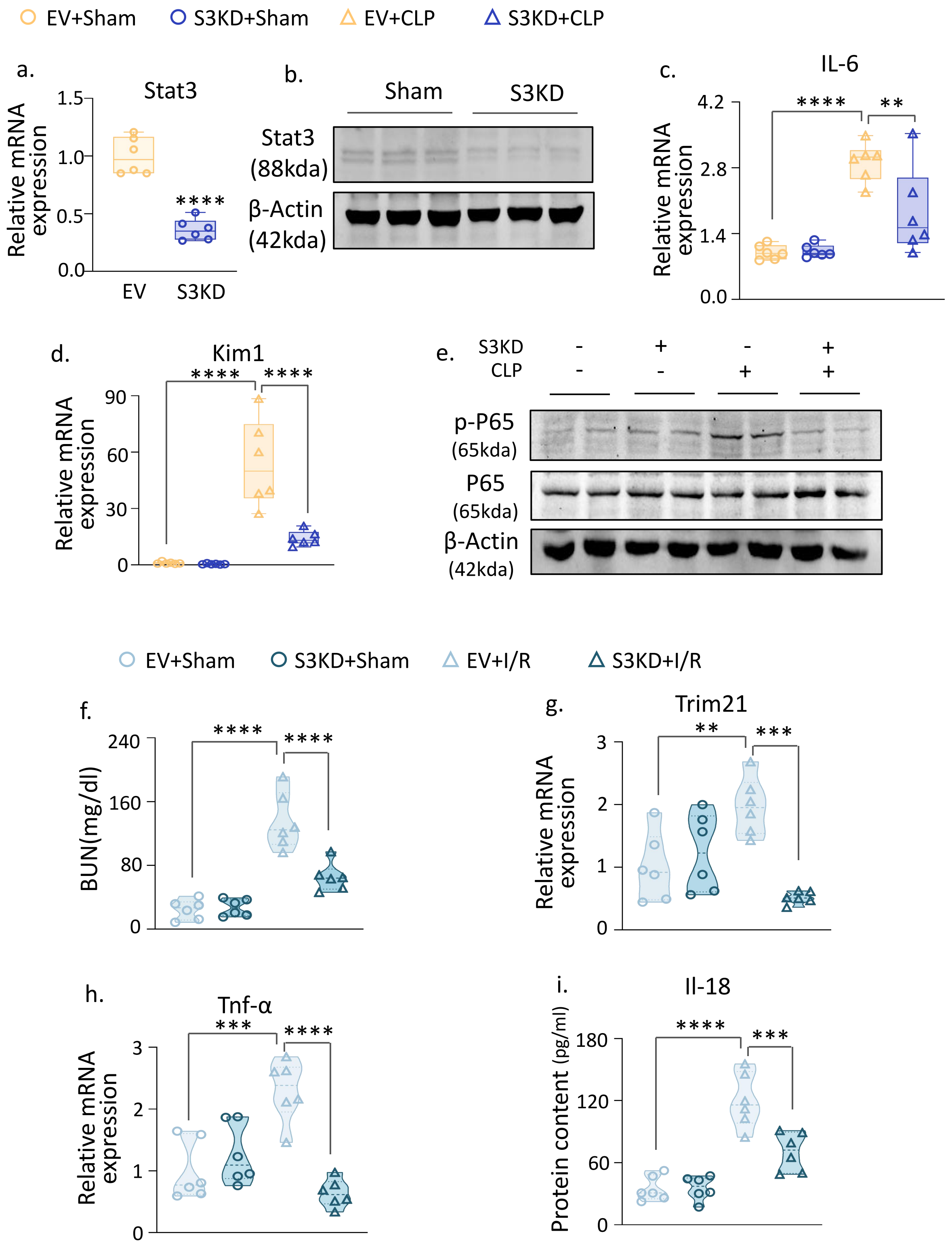

EV+Sham
S3KD+Sham
EV+CLP
S3KD+CLP
a.
Stat3
Relative mRNA expression
1.5
1.0
0.5
0.0
****
EV
S3KD
b.
Sham
S3KD
Stat3 (88kda)
β-Actin (42kda)
c.
IL-6
Relative mRNA expression
4.2
2.8
1.4
0.0
****
**
d.
Kim1
Relative mRNA expression
90
60
30
0
****
****
e.
S3KD - + - +
CLP - - + +
p-P65 (65kda)
P65 (65kda)
β-Actin (42kda)
EV+Sham
S3KD+Sham
EV+I/R
S3KD+I/R
f.
BUN(mg/dl)
240
160
80
0
****
****
g.
Trim21
Relative mRNA expression
3
2
1
0
**
***
h.
Tnf-α
Relative mRNA expression
3
2
1
0
***
****
i.
Il-18
Protein content (pg/ml)
180
120
60
0
****
***

Figure S8

a.

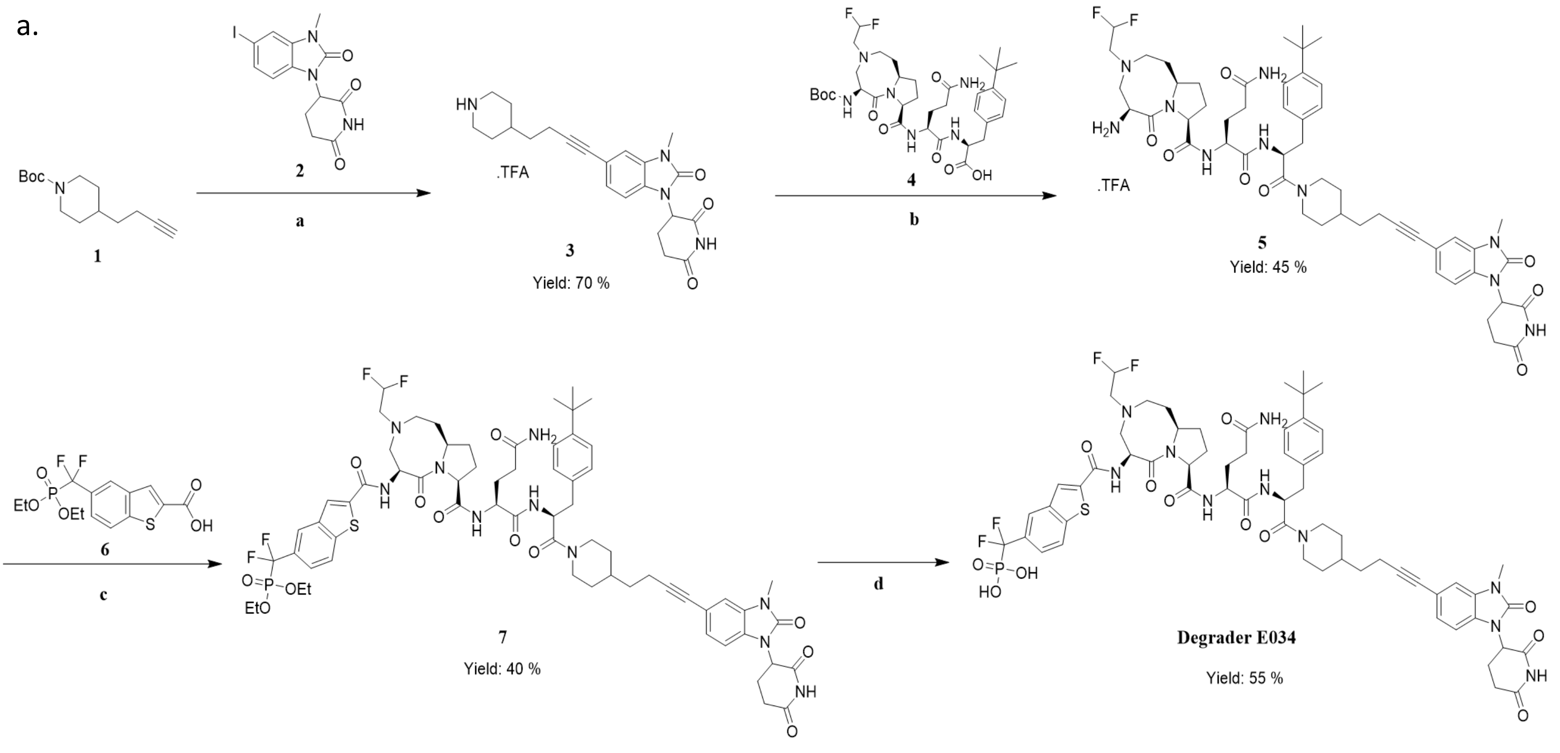
Boc
1
2
a
HN
.TFA
3
Yield: 70 %
4
b
H2N
.TFA
5
Yield: 45 %
6
c
7
Yield: 40 %
d
Degrader E034
Yield: 55 %

Figure S9

a.

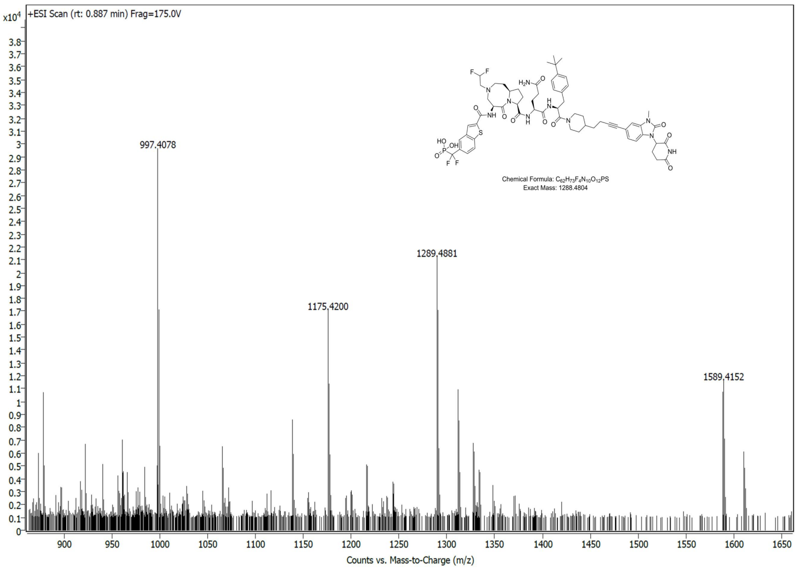

HRMS (ESI) (m/z): calcd for $C_{62}H_{73}F_4N_{10}O_{12}PS$ [M + H]+, 1289.4882; found, 1289.4881.

b.

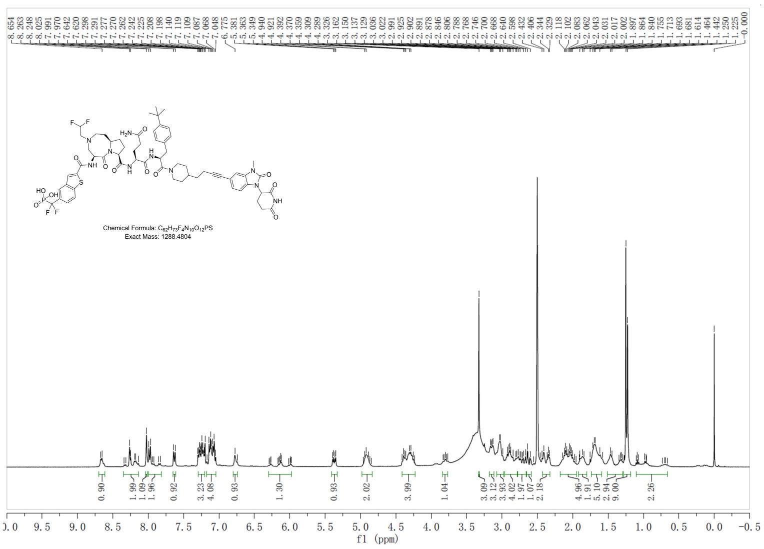

^{1}H NMR (400 MHz, DMSO) δ 8.66 (d, J = 6.8 Hz, 1H), 8.35 – 8.13 (m, 2H), 8.02 (s, 1H), 8.00 – 7.81 (m, 2H), 7.63 (d, J = 8.8 Hz, 1H), 7.30 – 7.20 (m, 3H), 7.17 – 7.05 (m, 4H), 6.76 (d, J = 13.2 Hz, 1H), 6.30 – 5.97 (m, 1H), 5.41 – 5.33 (m, 1H), 4.98 – 4.84 (m, 2H), 4.42 – 4.23 (m, 4H), 3.84 – 3.77 (m, 1H), 3.33 (s, 3H), 3.18 – 3.11 (m, 3H), 3.08 – 2.97 (m, 4H), 2.96 – 2.79 (m, 4H), 2.78 – 2.66 (m, 2H), 2.65 – 2.58 (m, 1H), 2.44 – 2.32 (m, 2H), 2.18 – 1.96 (m, 5H), 1.92 – 1.80 (m, 2H), 1.74 – 1.59 (m, 5H), 1.51 – 1.29 (m, 3H), 1.24 (d, J = 10.0 Hz, 9H), 1.11 – 0.66(m, 2H).

c.

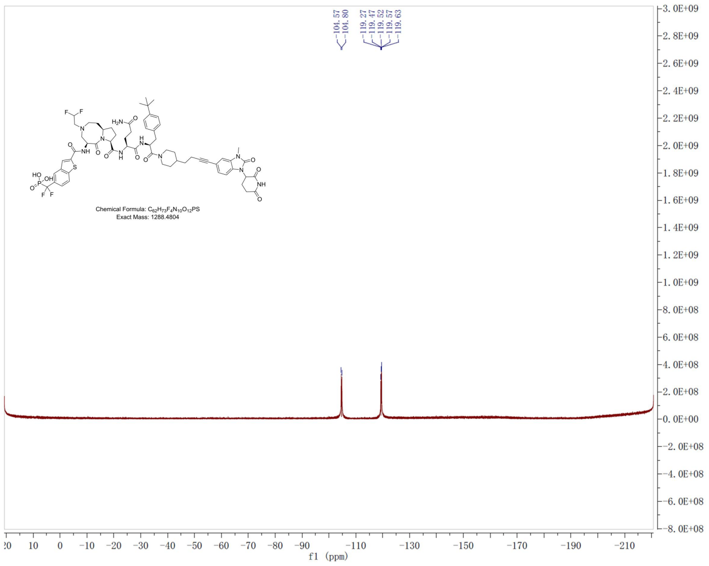

d.

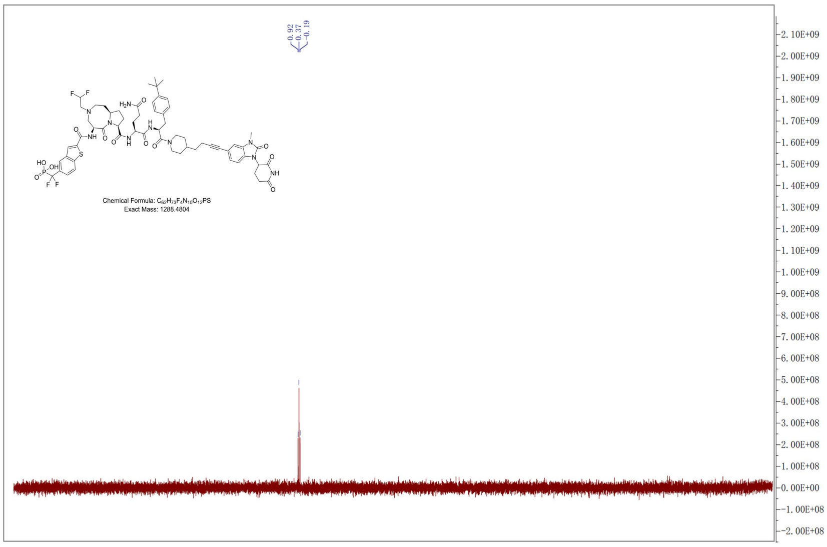

e.

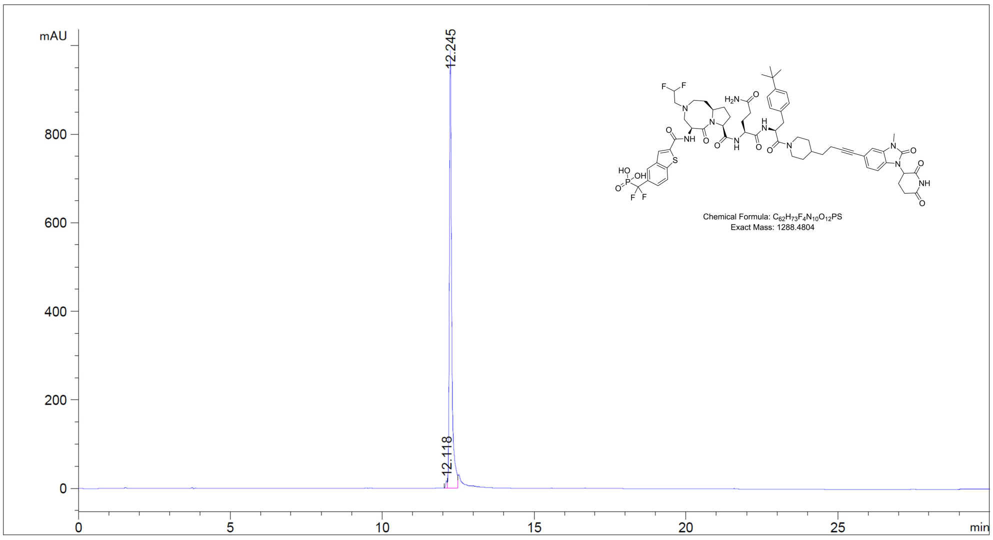

#	[min]		[min]	[mAU*s]	[mAU]	%
1	12.118	BV	0.0496	53.18842	16.48876	0.9840
2	12.245	VV	0.0812	5351.96289	987.94324	99.0160

Figure S10

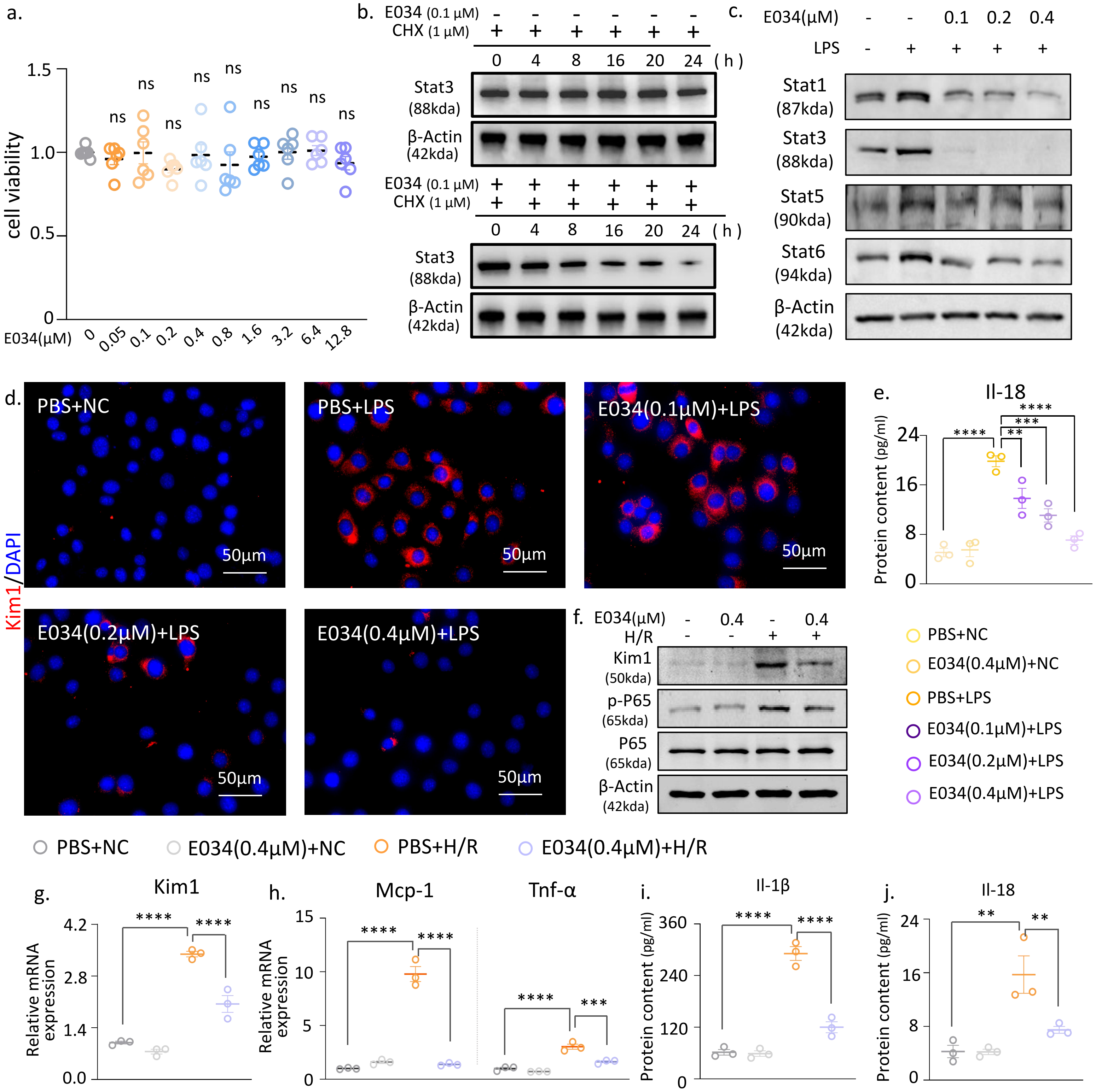

a.
cell viability
E034(μM)
0 0.05 0.1 0.2 0.4 0.8 1.6 3.2 6.4 12.8
ns
b.
E034 (0.1 μM)
CHX (1 μM)
0 4 8 16 20 24 (h)
Stat3 (88kda)
β-Actin (42kda)
c.
E034(μM) - - 0.1 0.2 0.4
LPS - + + + +
Stat1 (87kda)
Stat3 (88kda)
Stat5 (90kda)
Stat6 (94kda)
β-Actin (42kda)
d.
Kim1/DAPI
PBS+NC
PBS+LPS
E034(0.1μM)+LPS
E034(0.2μM)+LPS
E034(0.4μM)+LPS
50μm
e.
Il-18
Protein content (pg/ml)
PBS+NC
E034(0.4μM)+NC
PBS+LPS
E034(0.1μM)+LPS
E034(0.2μM)+LPS
E034(0.4μM)+LPS
f.
E034(μM) - 0.4 - 0.4
H/R - - + +
Kim1 (50kda)
p-P65 (65kda)
P65 (65kda)
β-Actin (42kda)
PBS+NC
E034(0.4μM)+NC
PBS+H/R
E034(0.4μM)+H/R
g.
Kim1
Relative mRNA expression
h.
Mcp-1
Tnf-α
i.
Il-1β
j.
Il-18

Figure S11

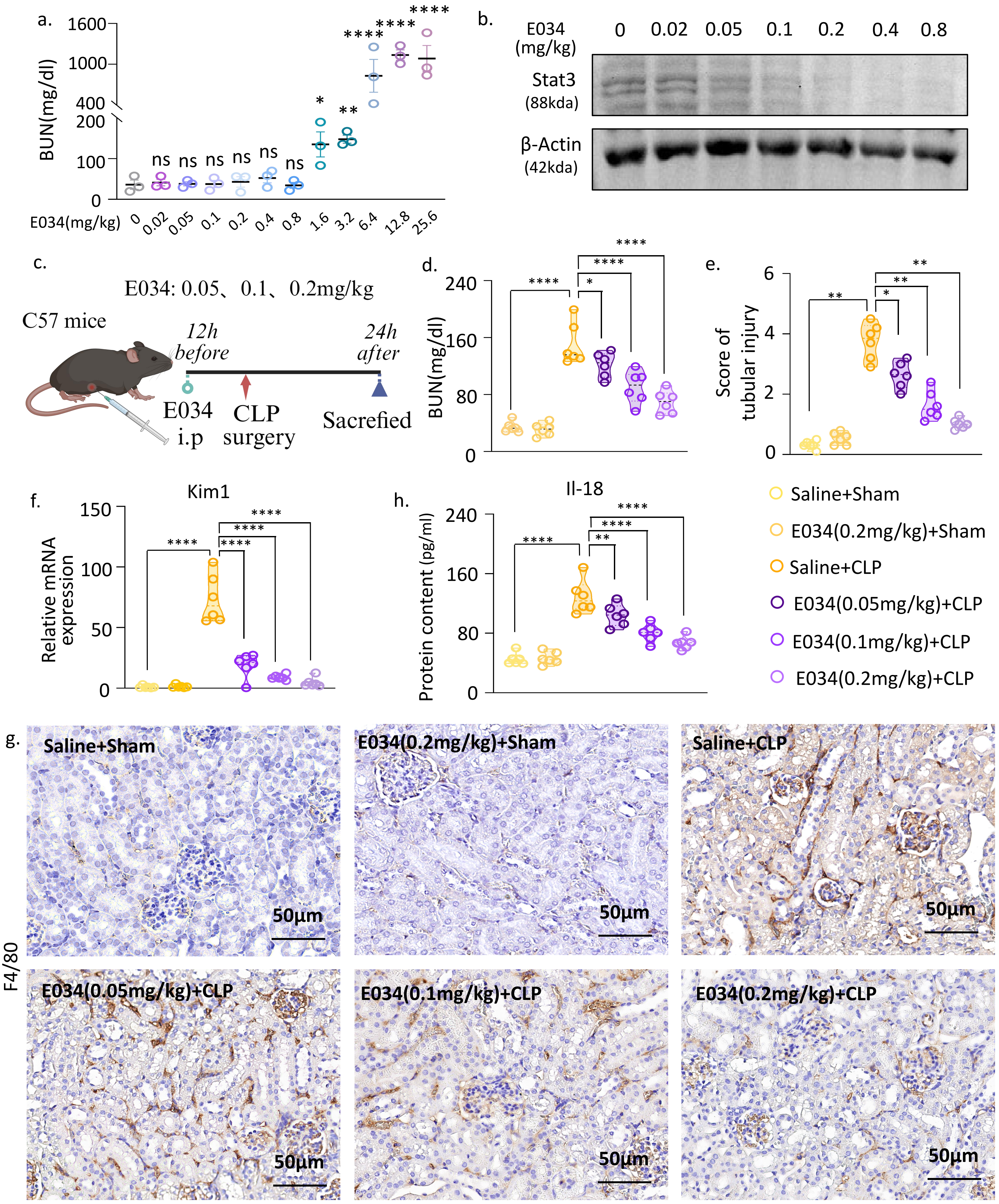
a.
BUN(mg/dl)
E034(mg/kg)
0 0.02 0.05 0.1 0.2 0.4 0.8 1.6 3.2 6.4 12.8 25.6
ns ns ns ns ns ns * ** **** **** ****
b.
E034 (mg/kg)
0 0.02 0.05 0.1 0.2 0.4 0.8
Stat3 (88kda)
β-Actin (42kda)
c.
E034: 0.05、0.1、0.2mg/kg
C57 mice
12h before
24h after
E034 i.p
CLP surgery
Sacrefied
d.
BUN(mg/dl)
e.
Score of tubular injury
f.
Kim1
Relative mRNA expression
h.
Il-18
Protein content (pg/ml)
Saline+Sham
E034(0.2mg/kg)+Sham
Saline+CLP
E034(0.05mg/kg)+CLP
E034(0.1mg/kg)+CLP
E034(0.2mg/kg)+CLP
g.
F4/80
Saline+Sham
E034(0.2mg/kg)+Sham
Saline+CLP
E034(0.05mg/kg)+CLP
E034(0.1mg/kg)+CLP
E034(0.2mg/kg)+CLP
50μm

Figure S12

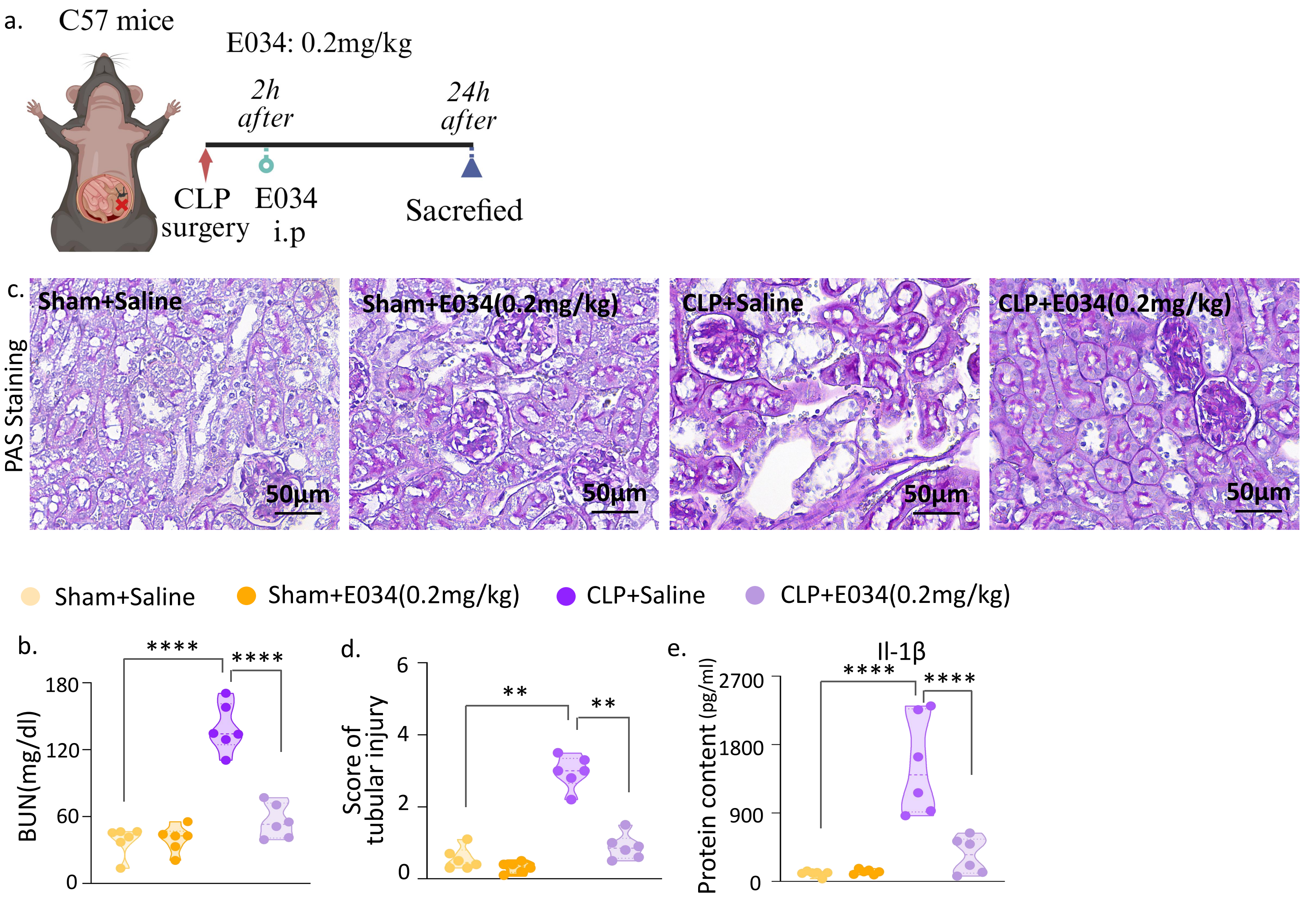

a.
C57 mice
E034: 0.2mg/kg
2h after
24h after
CLP surgery
E034 i.p
Sacrefied
c.
PAS Staining
Sham+Saline
Sham+E034(0.2mg/kg)
CLP+Saline
CLP+E034(0.2mg/kg)
50μm
Sham+Saline
Sham+E034(0.2mg/kg)
CLP+Saline
CLP+E034(0.2mg/kg)
b.
BUN(mg/dl)
****
0
60
120
180
d.
Score of tubular injury
**
0
2
4
6
e.
Il-1β
Protein content (pg/ml)
****
0
900
1800
2700

Figure S13

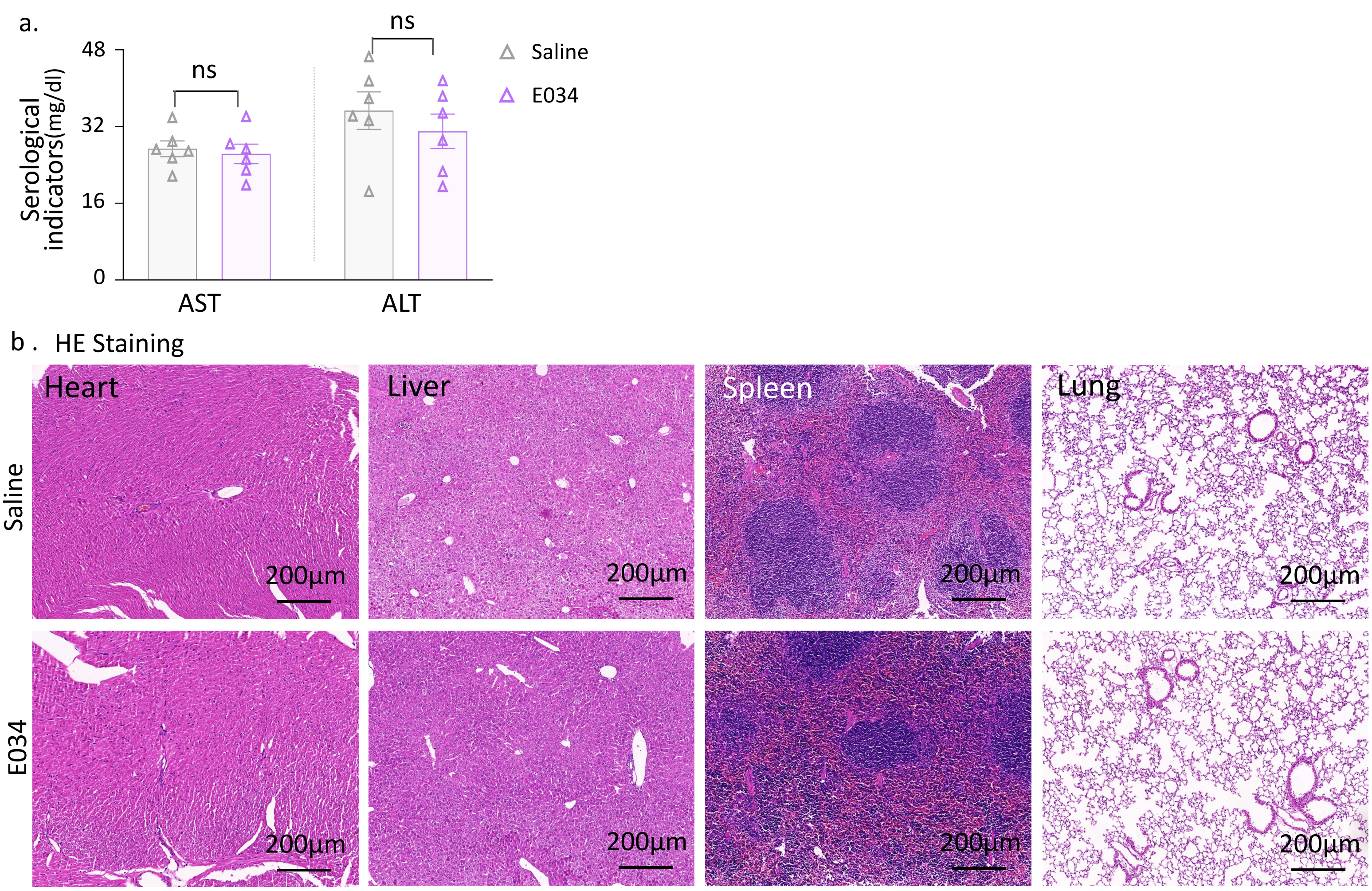
a.
Serological indicators(mg/dl)
48
32
16
0
ns
ns
AST
ALT
Saline
E034
b． HE Staining
Heart
Liver
Spleen
Lung
Saline
E034
200μm

Figure S14

a.

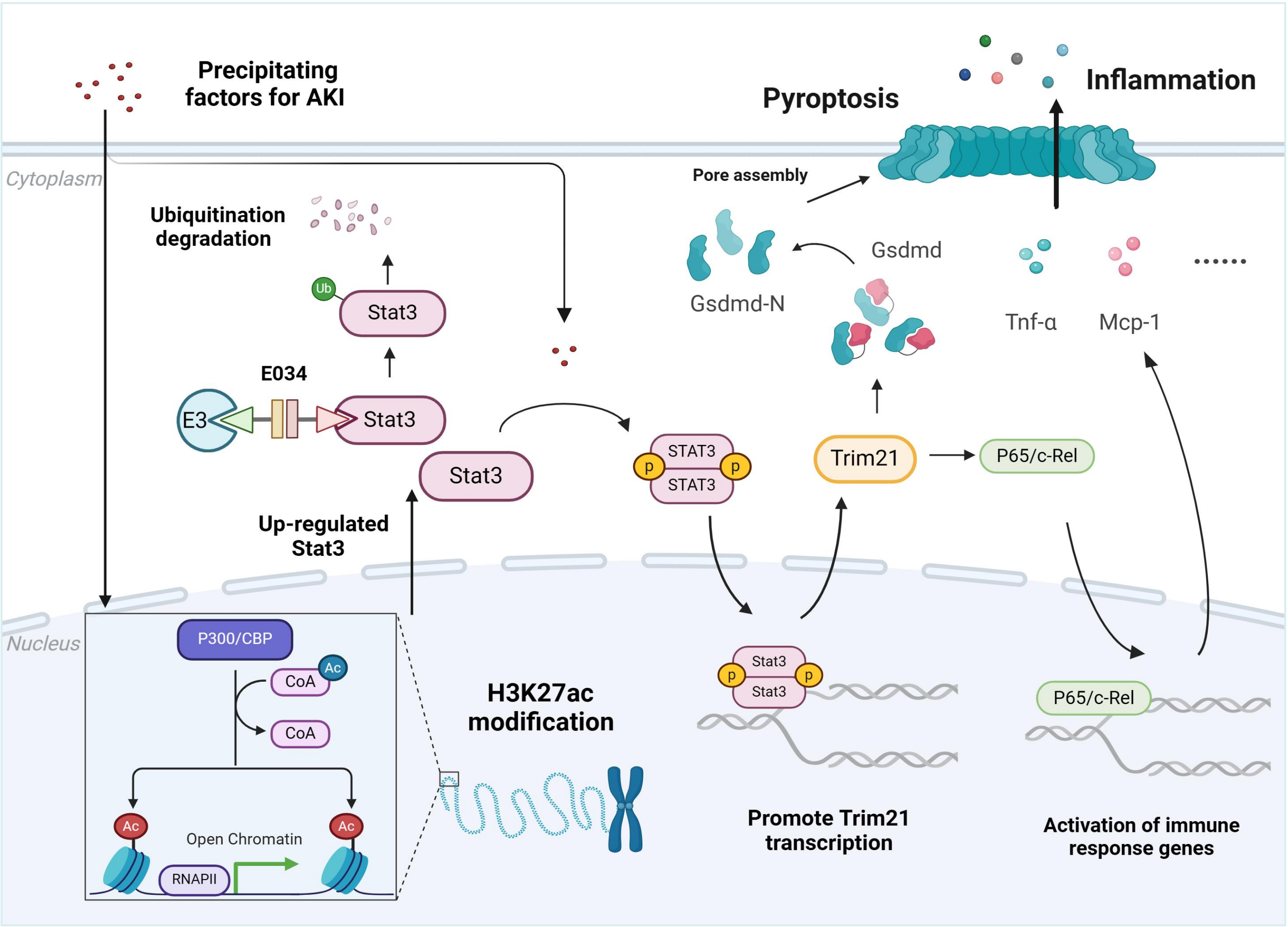

Precipitating factors for AKI
Pyroptosis
Inflammation
Cytoplasm
Pore assembly
Ubiquitination degradation
Gsdmd
Gsdmd-N
Ub
Stat3
Tnf-α
Mcp-1
E034
E3
Stat3
Stat3
STAT3
STAT3
p
p
Trim21
P65/c-Rel
Up-regulated Stat3
Nucleus
P300/CBP
CoA
Ac
CoA
H3K27ac modification
Stat3
Stat3
p
p
P65/c-Rel
Ac
Ac
Open Chromatin
RNAPII
Promote Trim21 transcription
Activation of immune response genes

Figure S15
