# Supplementary material for: Targeting Stat3 with conditional knockout or PROTAC technology alleviates renal injury by Limiting pyroptosis
Source: eBioMedicine. 2025 May 8;116:105739. doi: 10.1016/j.ebiom.2025.105739 (PMC12136849; doi:10.1016/j.ebiom.2025.105739)
Supplement: Supplementary Western blots [file mmc7.pdf]

Fig.1a

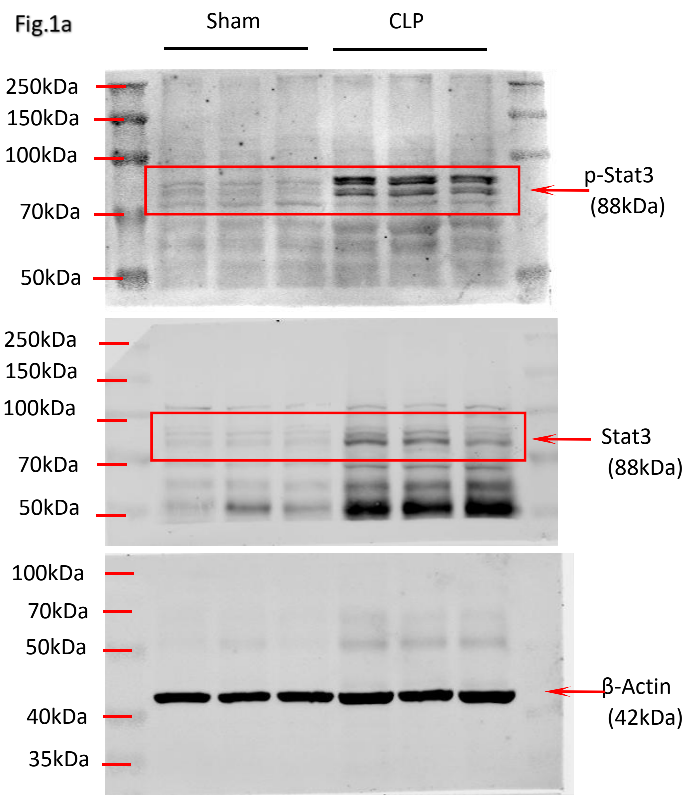

Fig.1c

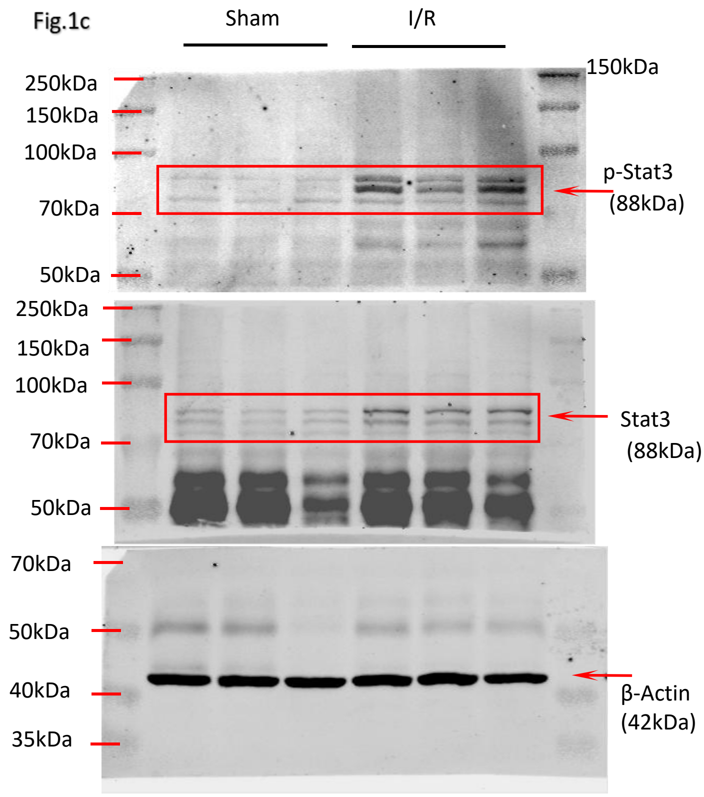

Fig.1h

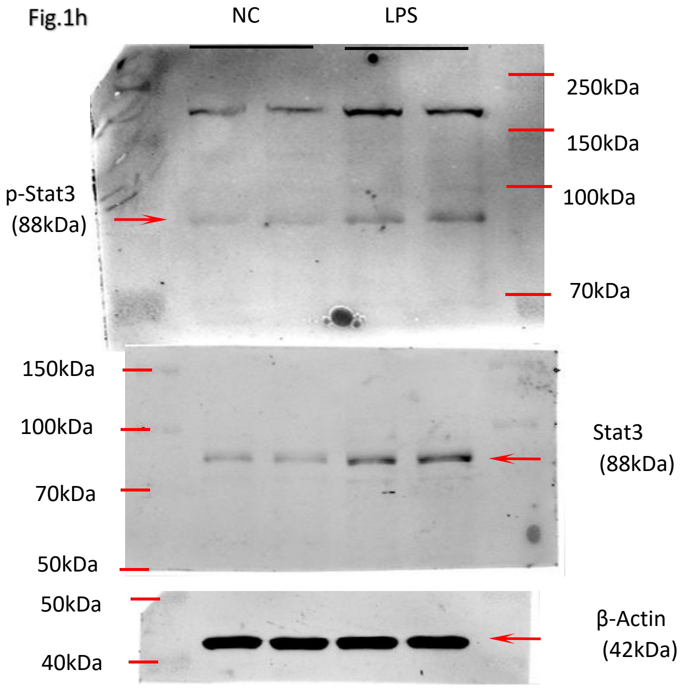

Fig.1j

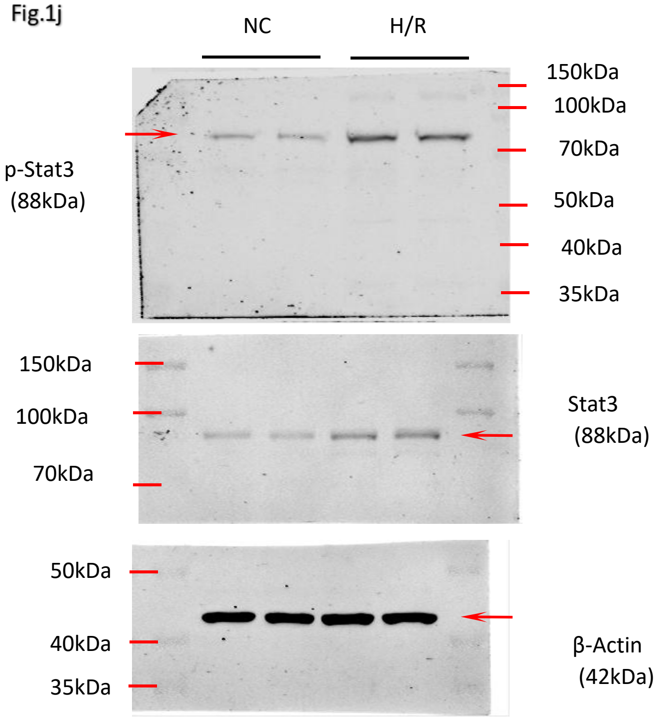

Fig.1l

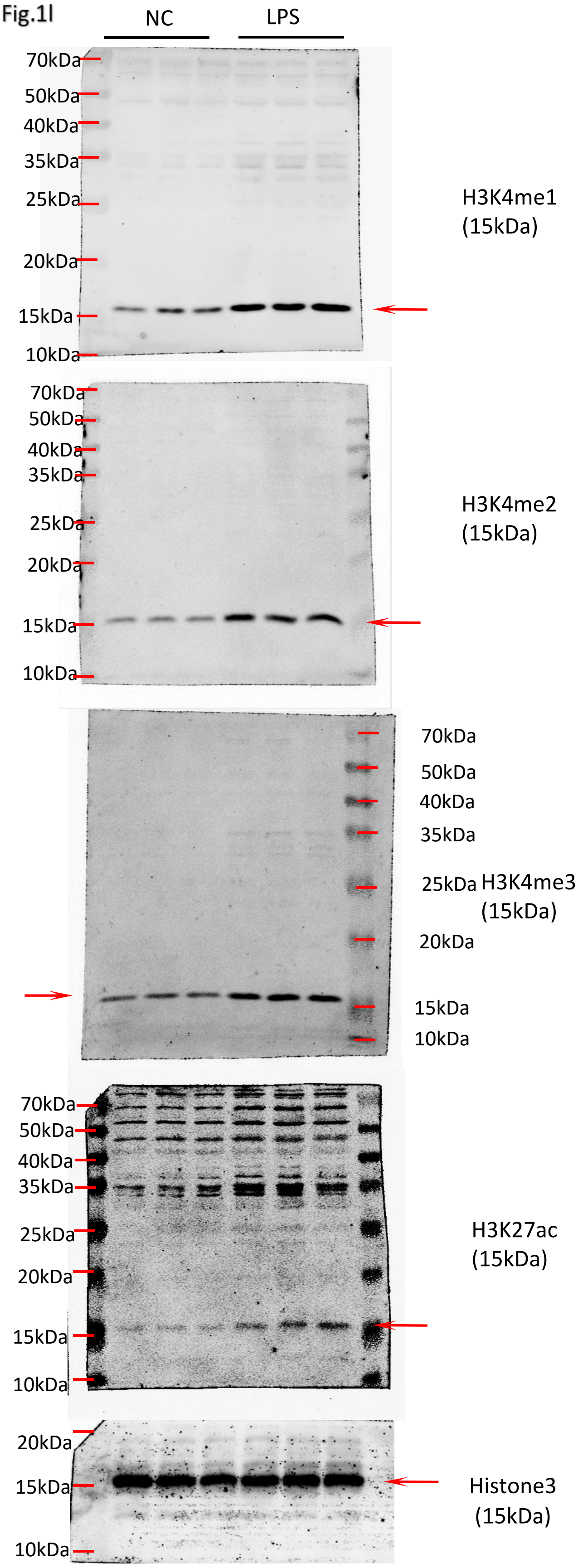

Fig.1n

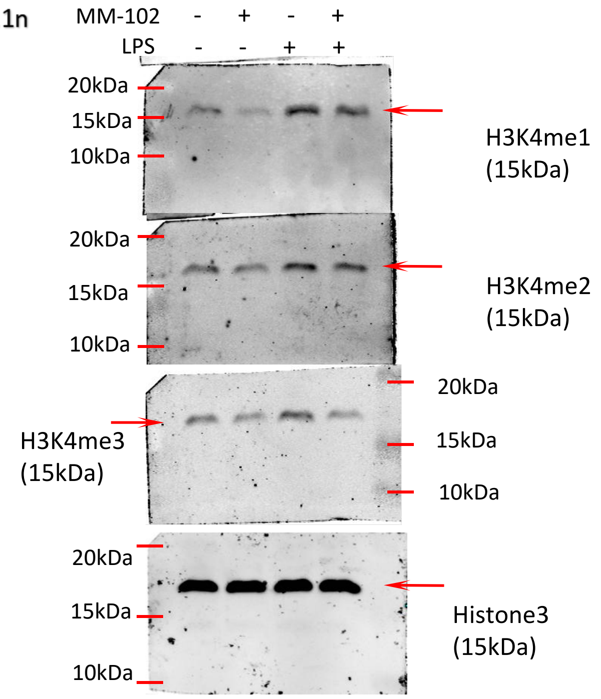

Fig.1o

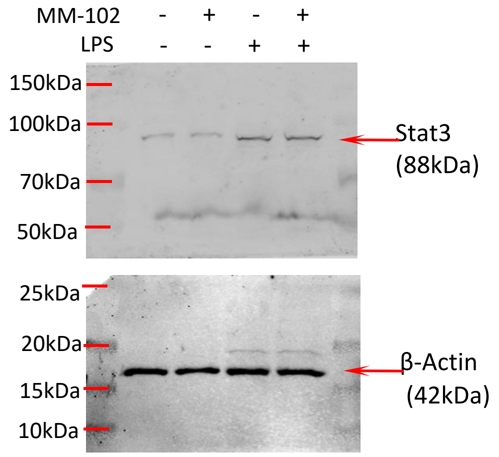

Fig.1p

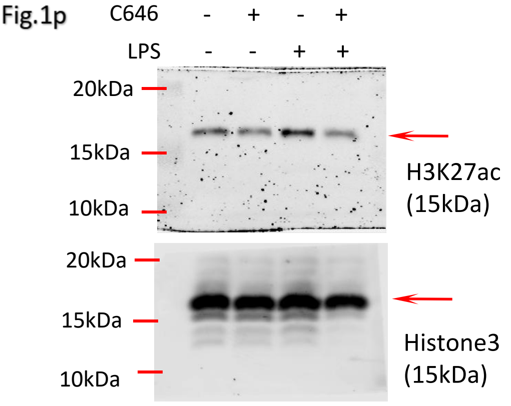

Fig.1q

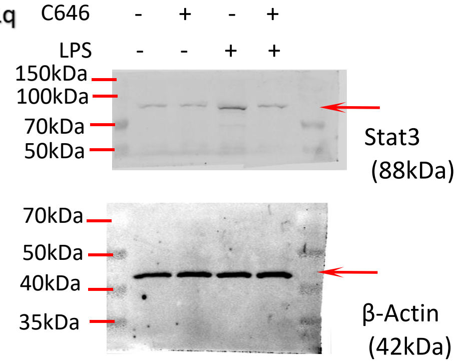

Fig.1s

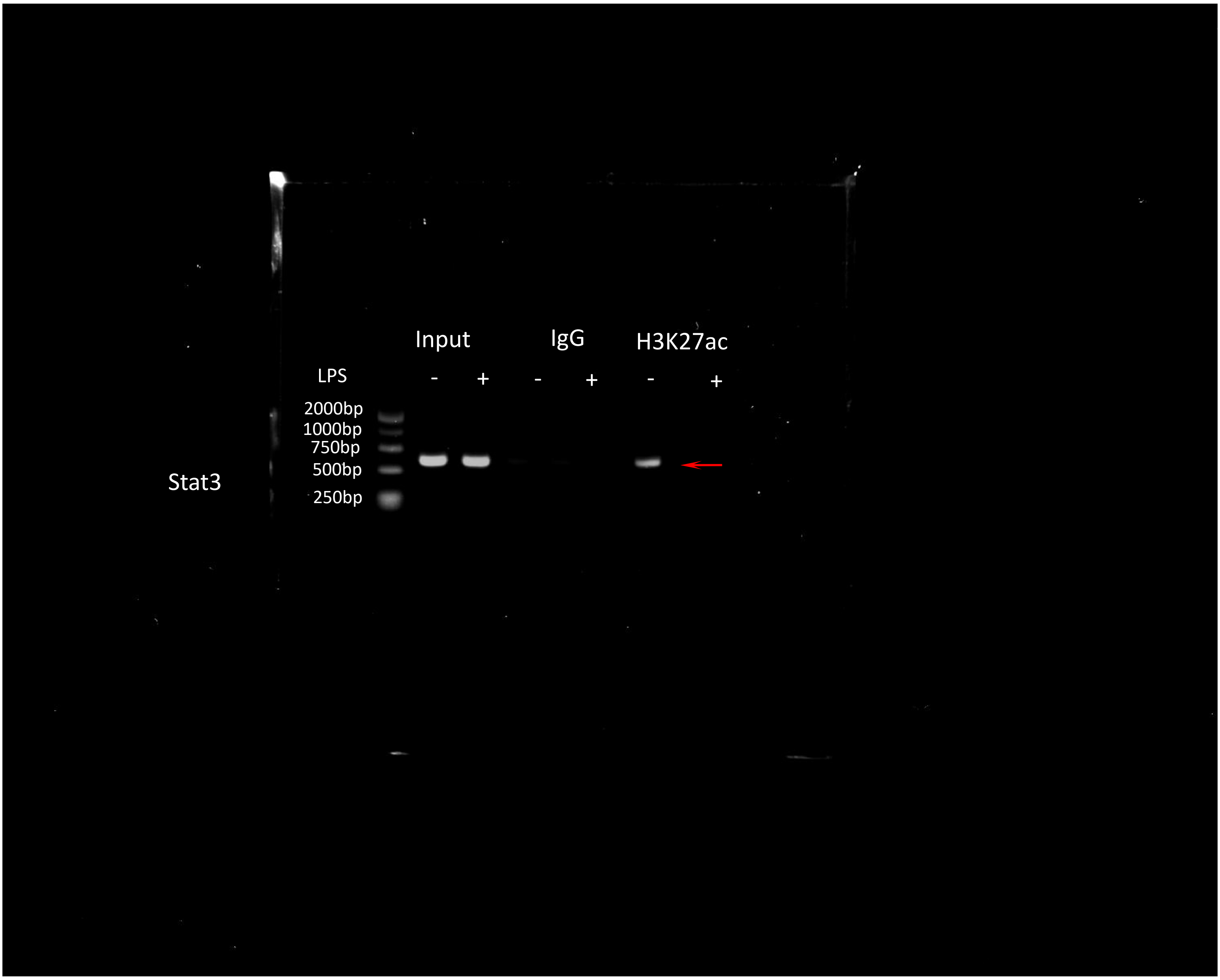
Input
IgG
H3K27ac
LPS
-
+
-
+
-
+
2000bp
1000bp
750bp
500bp
250bp
Stat3

Fig.2b

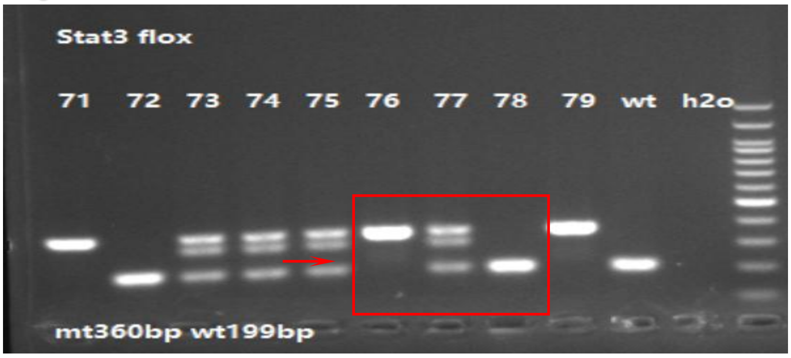
Stat3 flox
71 72 73 74 75 76 77 78 79 wt h2o
mt360bp wt199bp

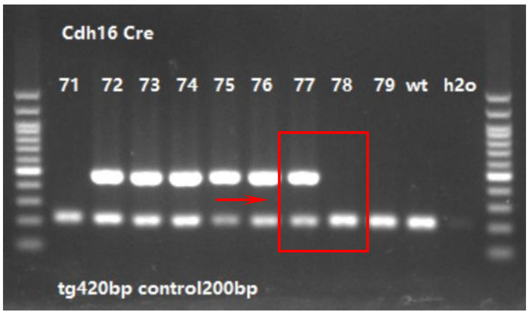
Cdh16 Cre
71 72 73 74 75 76 77 78 79 wt h2o
tg420bp control200bp

Fig.2e

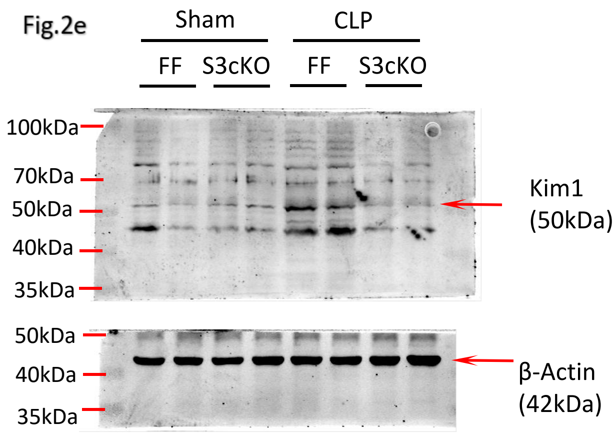

Fig.2g

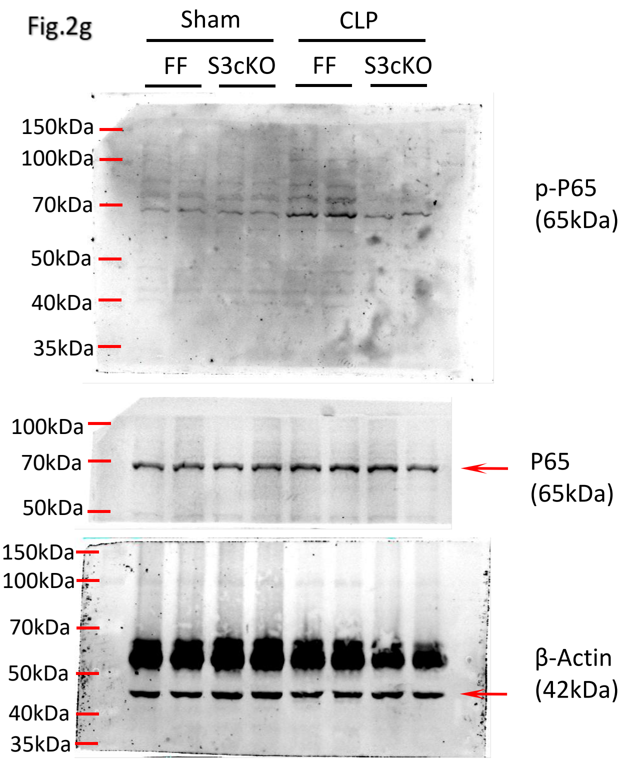

Fig.2n

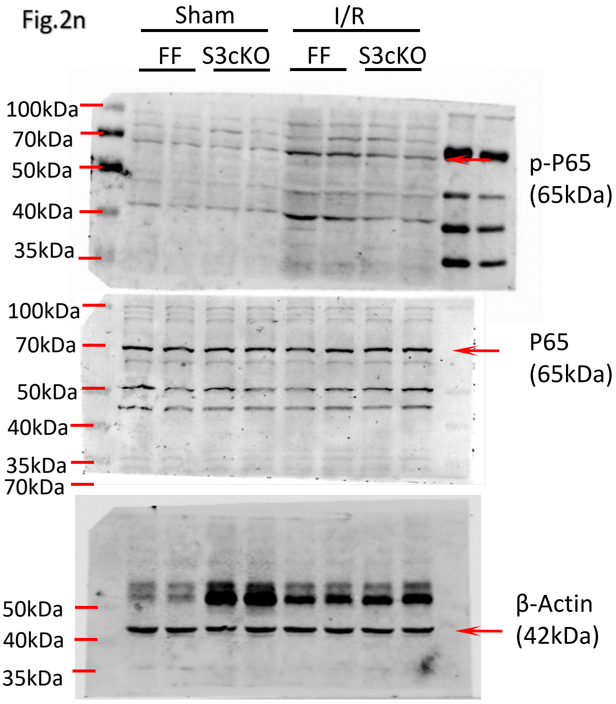

Fig.2t

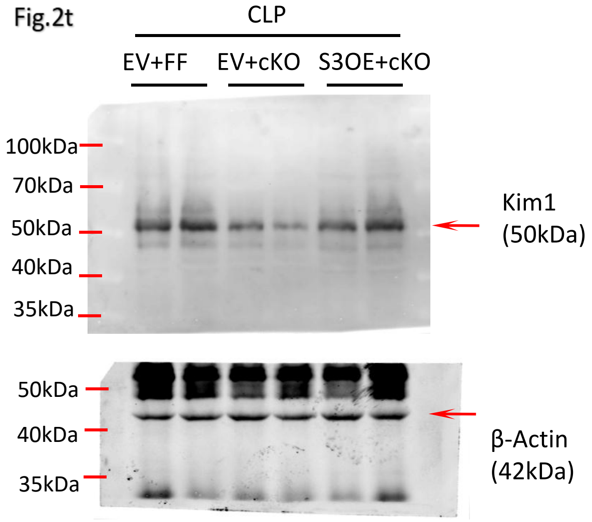

Fig.3a

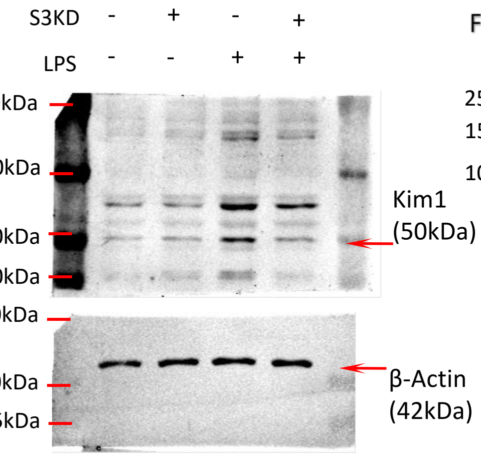

Fig.3f

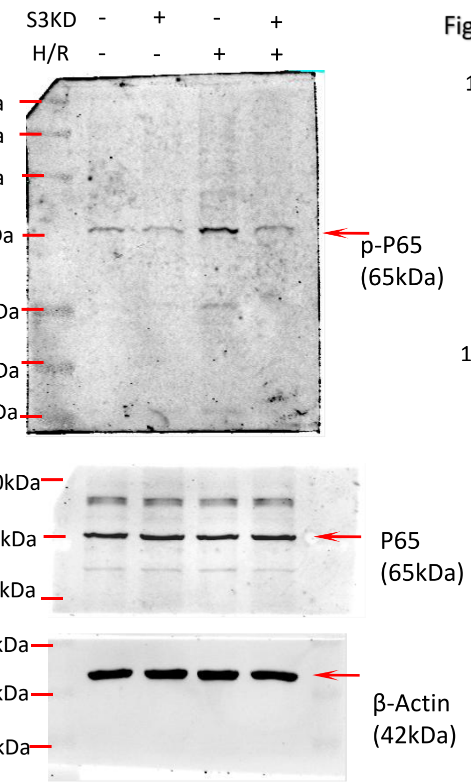

Fig.3n

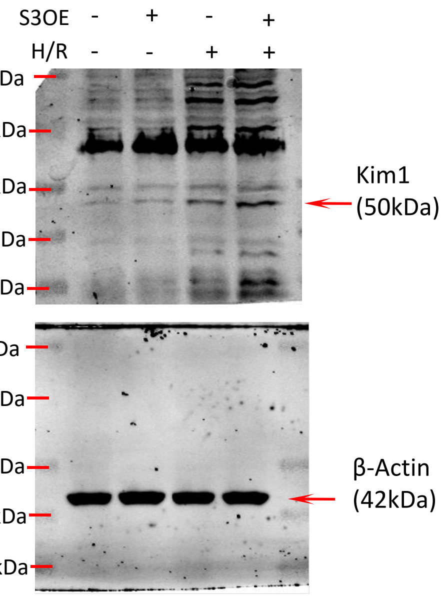

Fig.3d

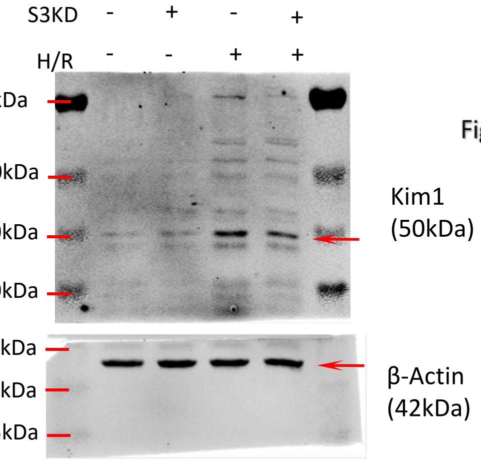

Fig.3i

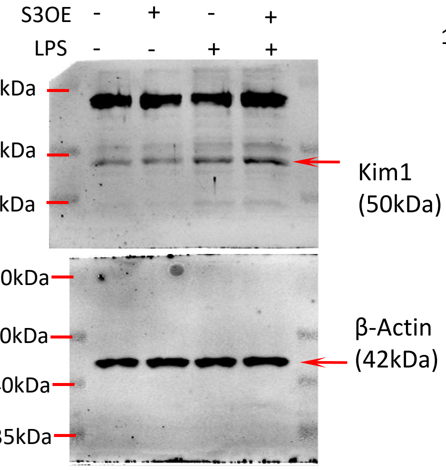

Fig.3o

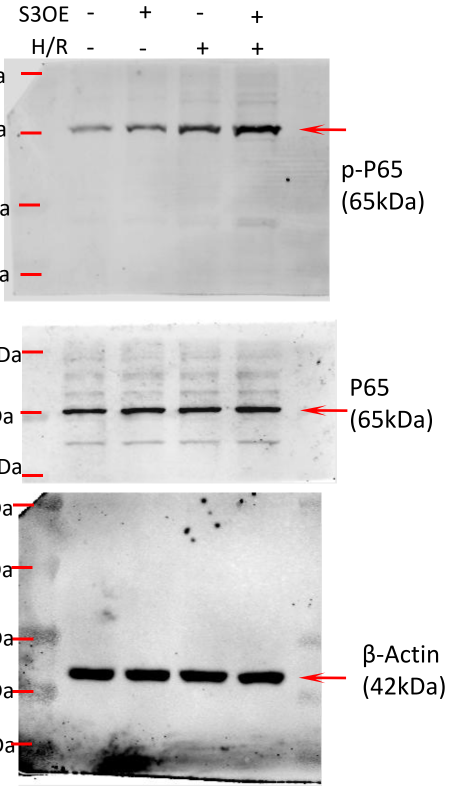

Fig.3e

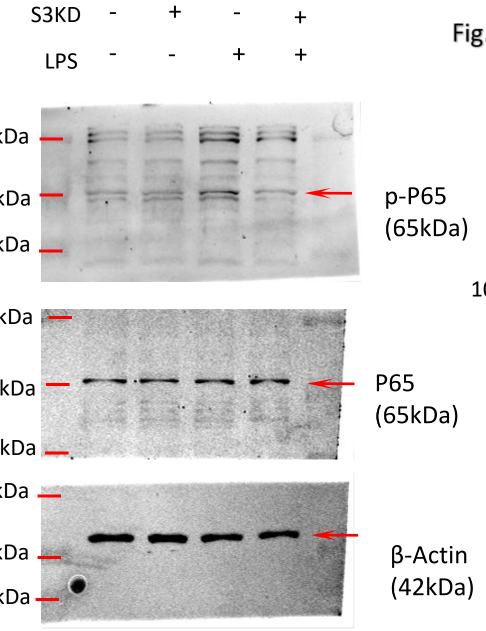

Fig.3j

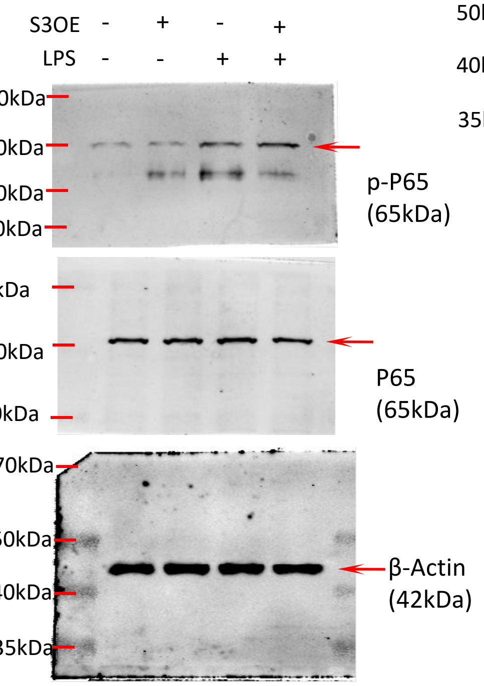

Fig.4j

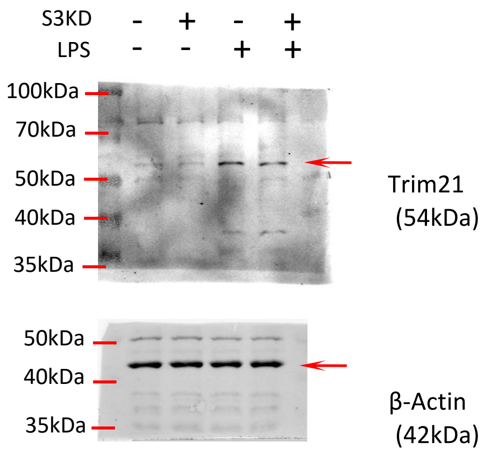

Fig.4m

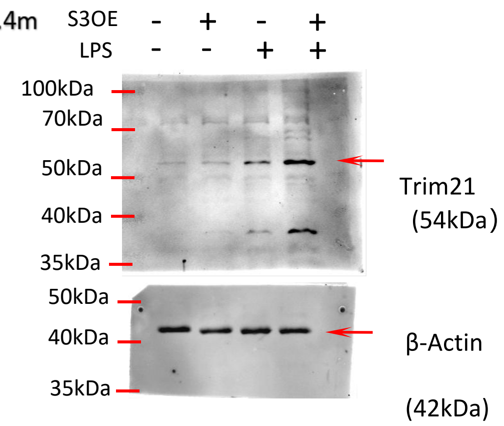

Fig.4k

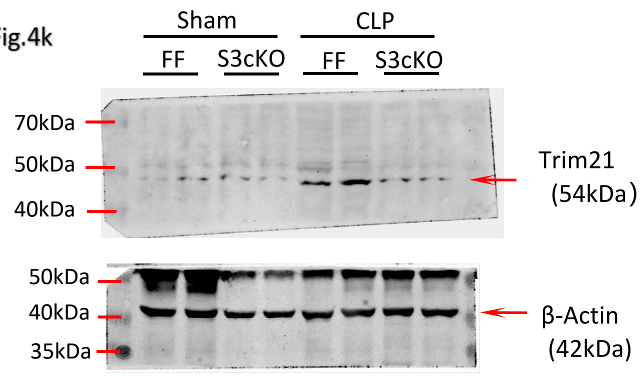

Fig.5a.

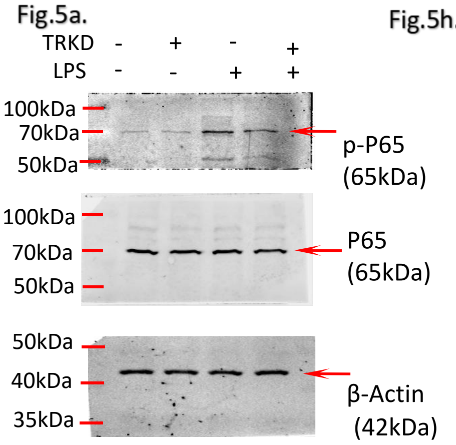

Fig.5h.

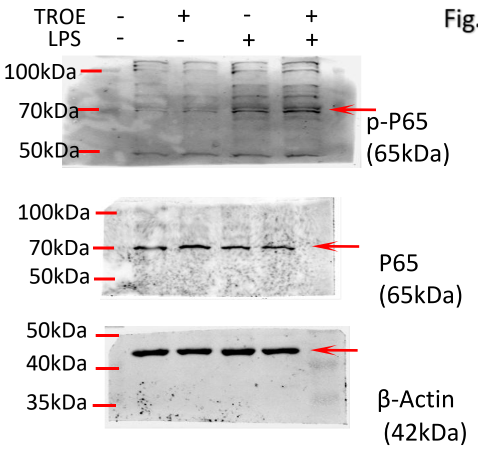

Fig.5k.

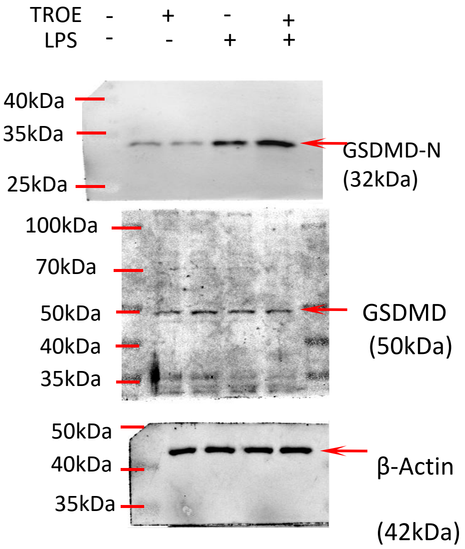

Fig.5d.

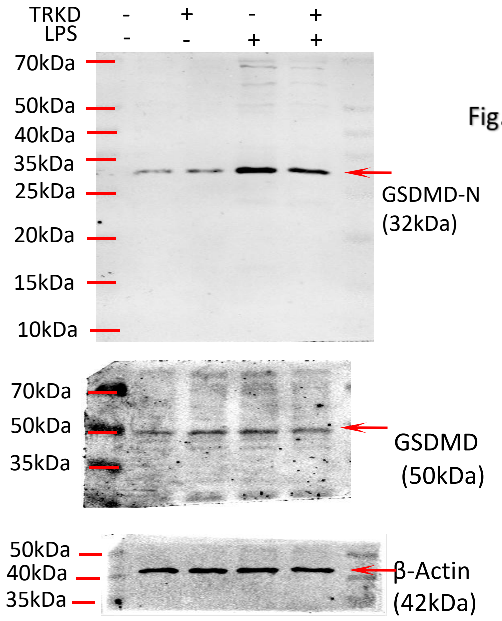

Fig.5n.

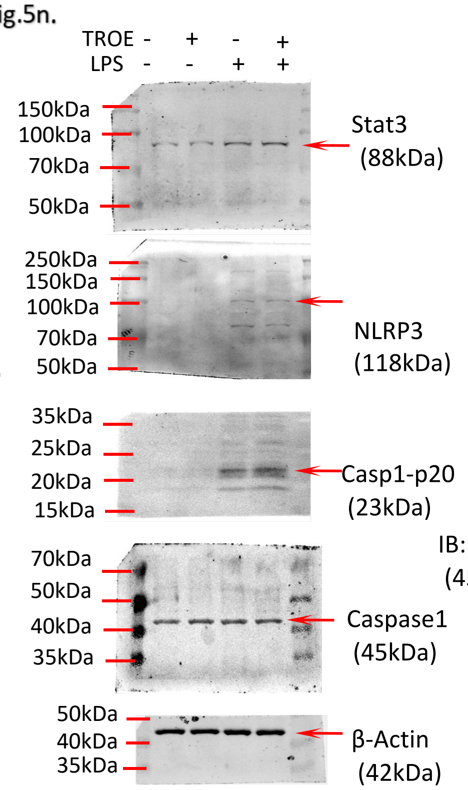

Fig.5o.

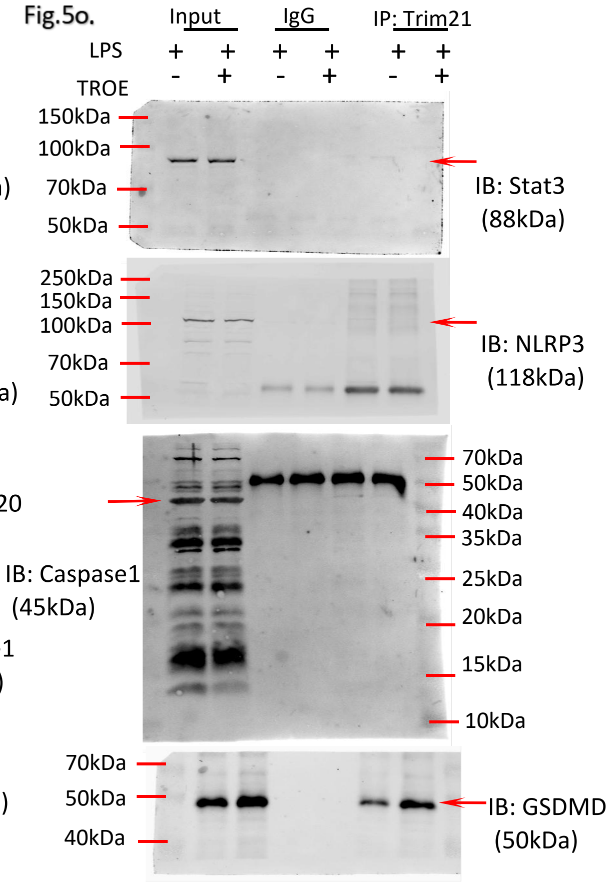

Fig.5q.

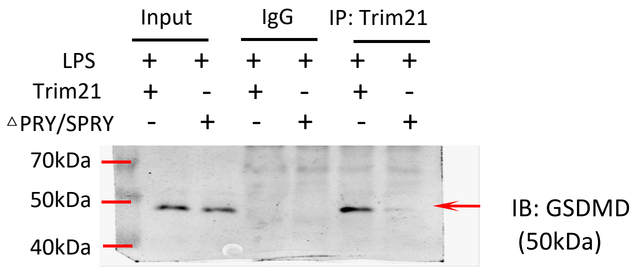

Fig.6a.

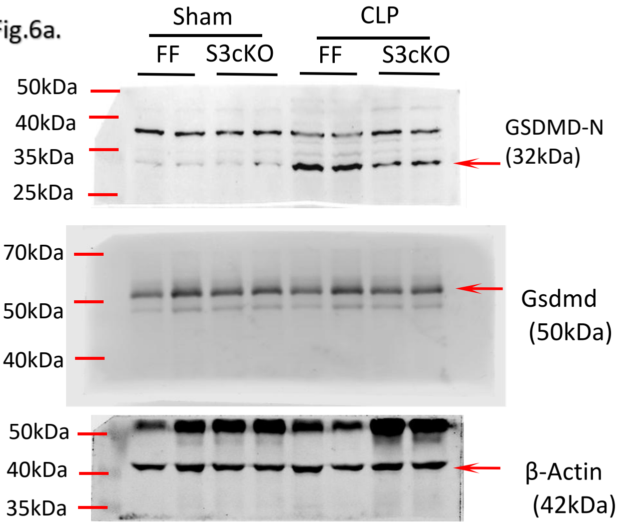

Fig.6j.

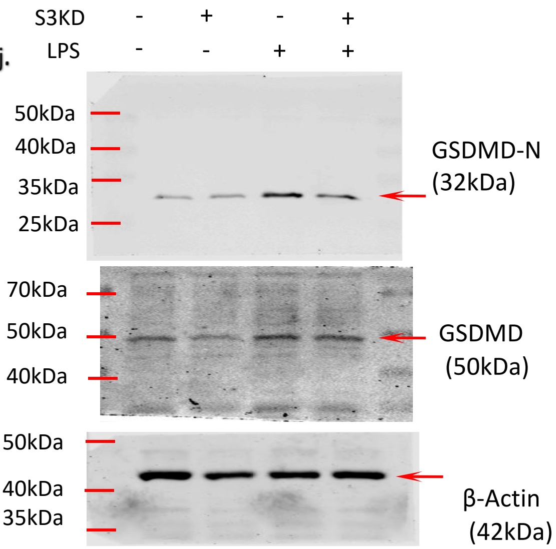

Fig.6m.

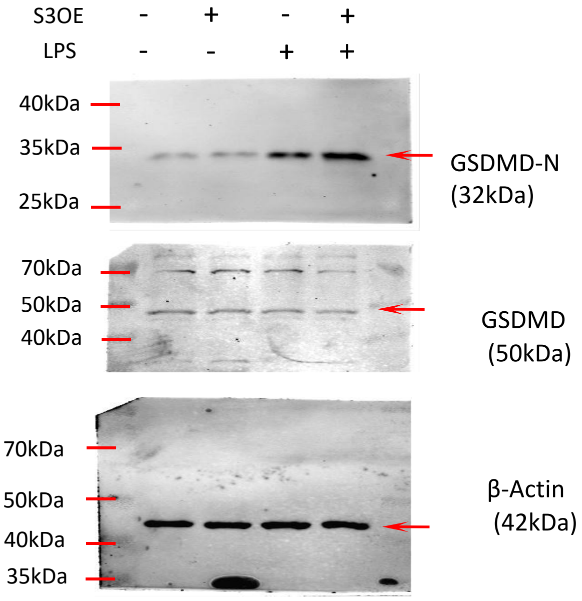

Fig.6n.

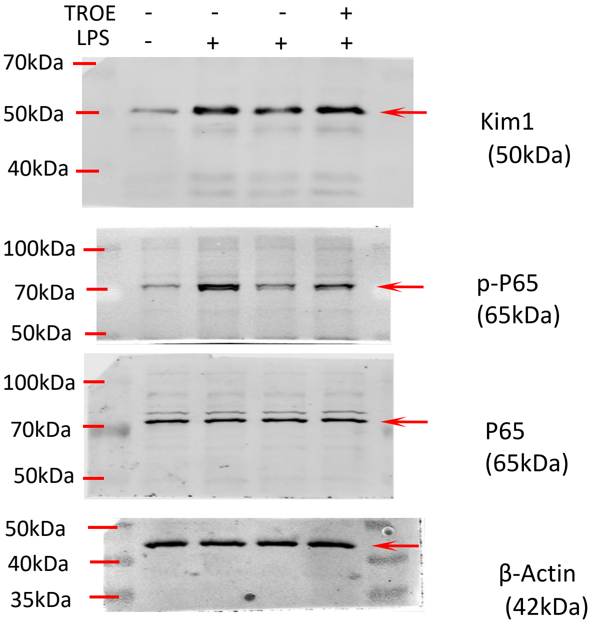

Fig.6p.

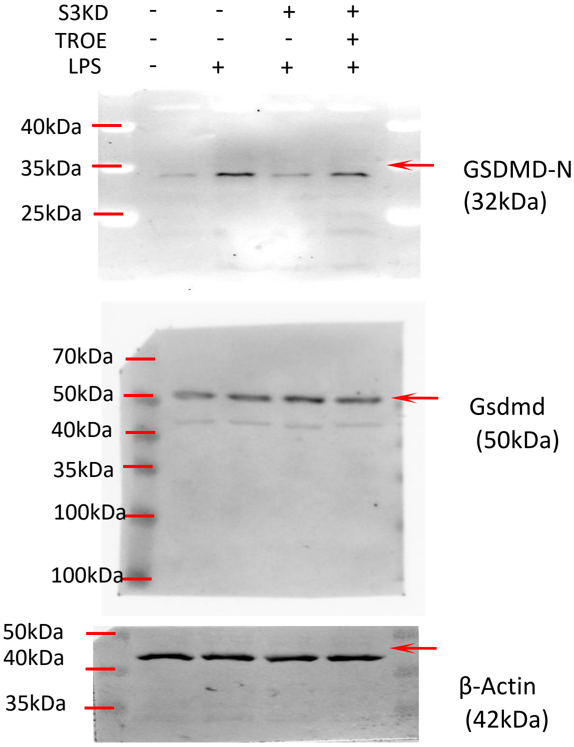

Fig.7b.

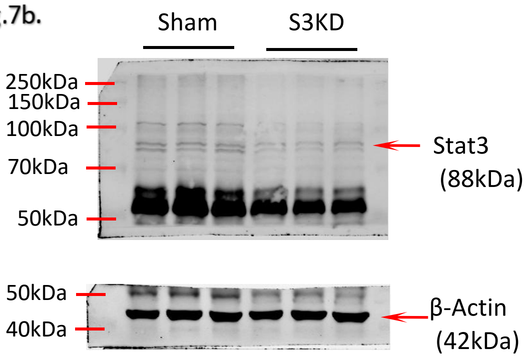

Fig.7h.

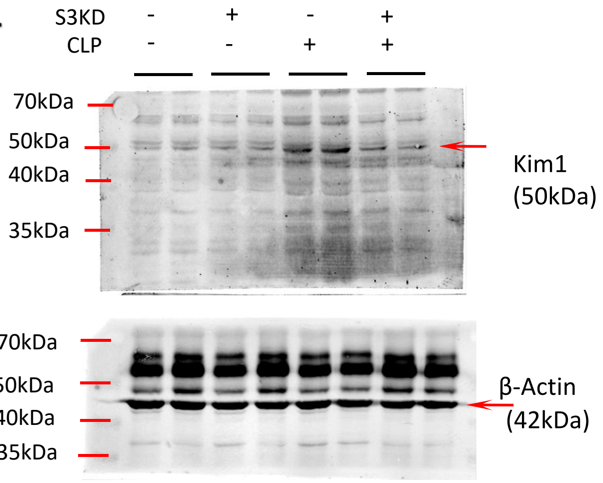

Fig.7i.

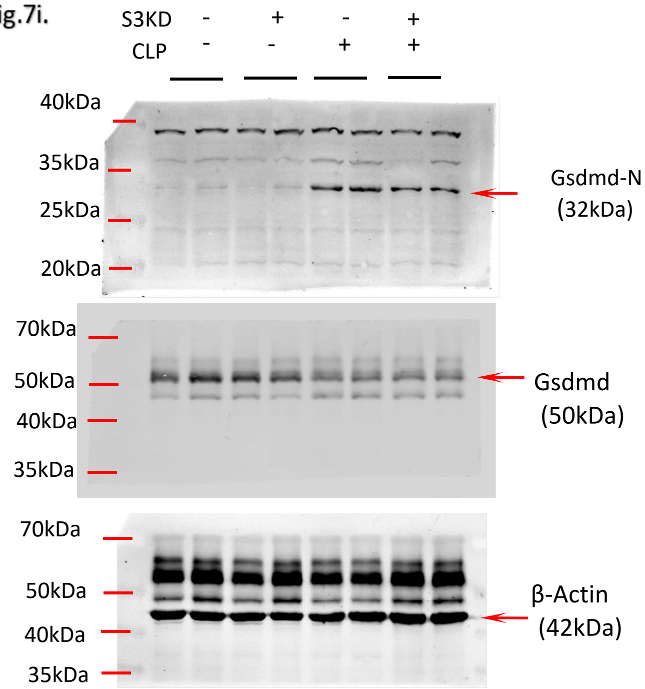

Fig.7o.

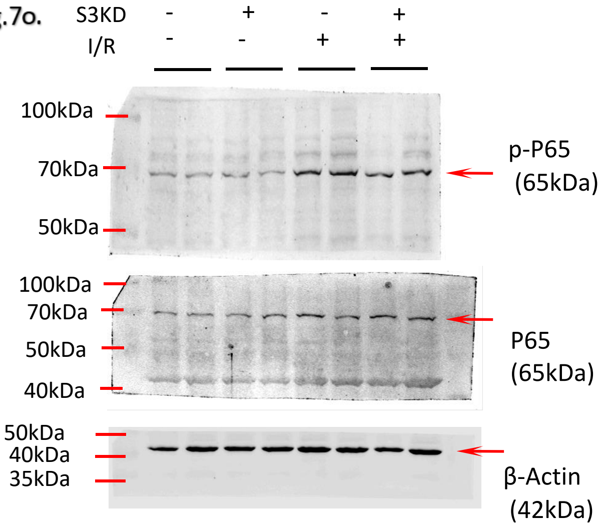

Fig.7q.

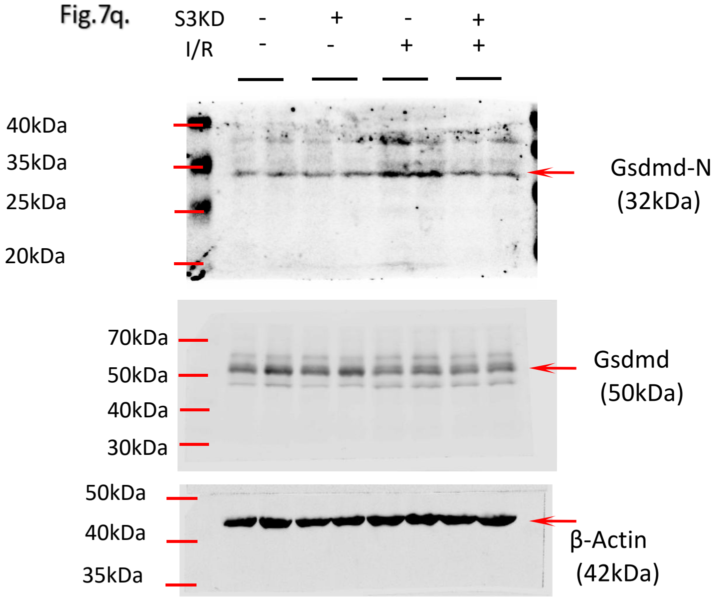

Fig.8b.

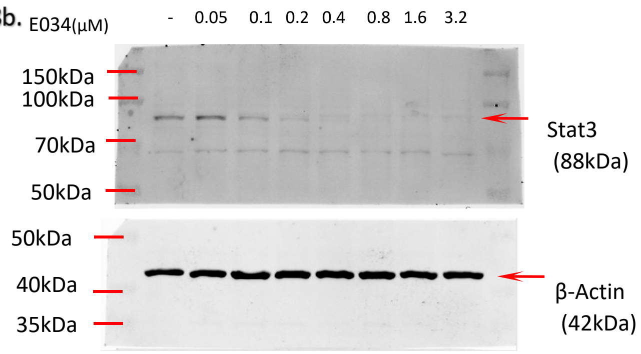

Fig.8c.

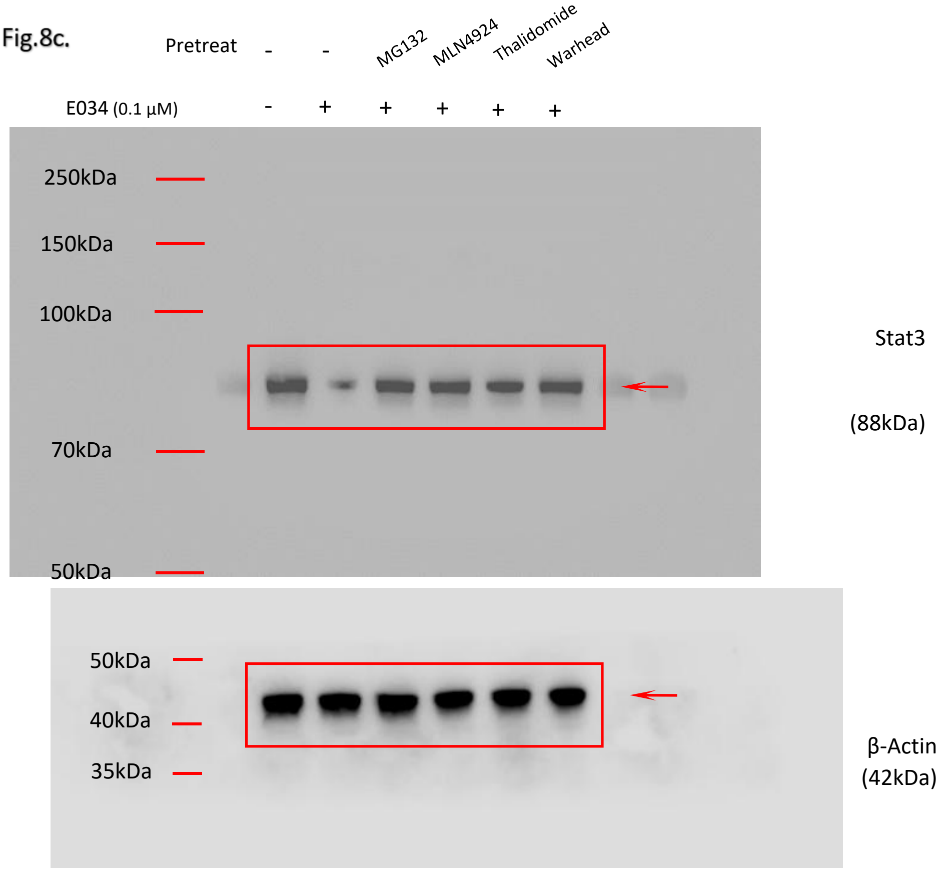

Fig.8e.

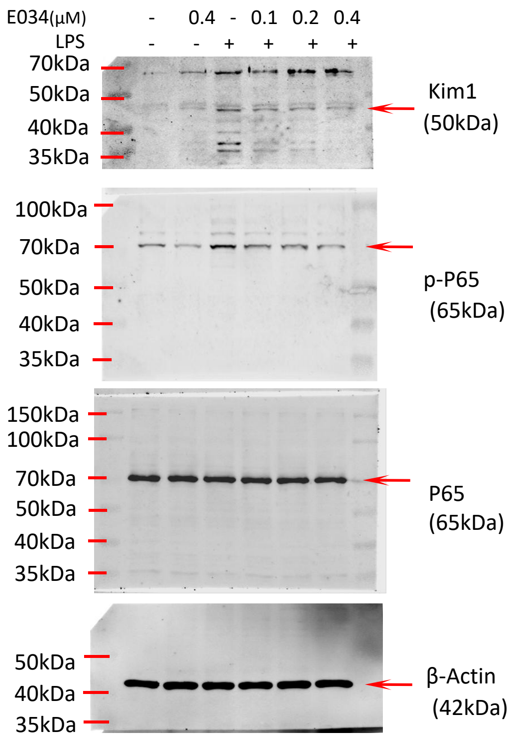

Fig.8g.

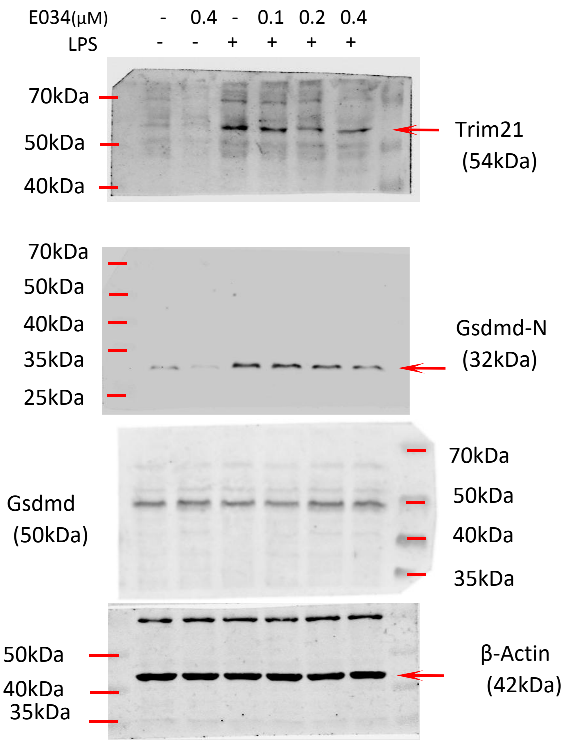

Fig.8k.

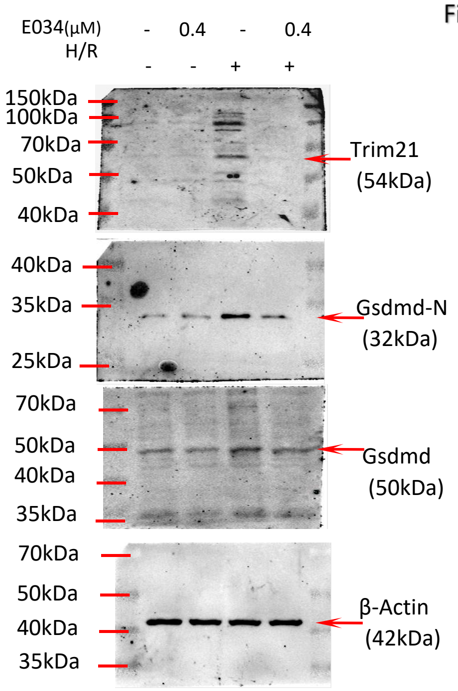

Fig.8n.

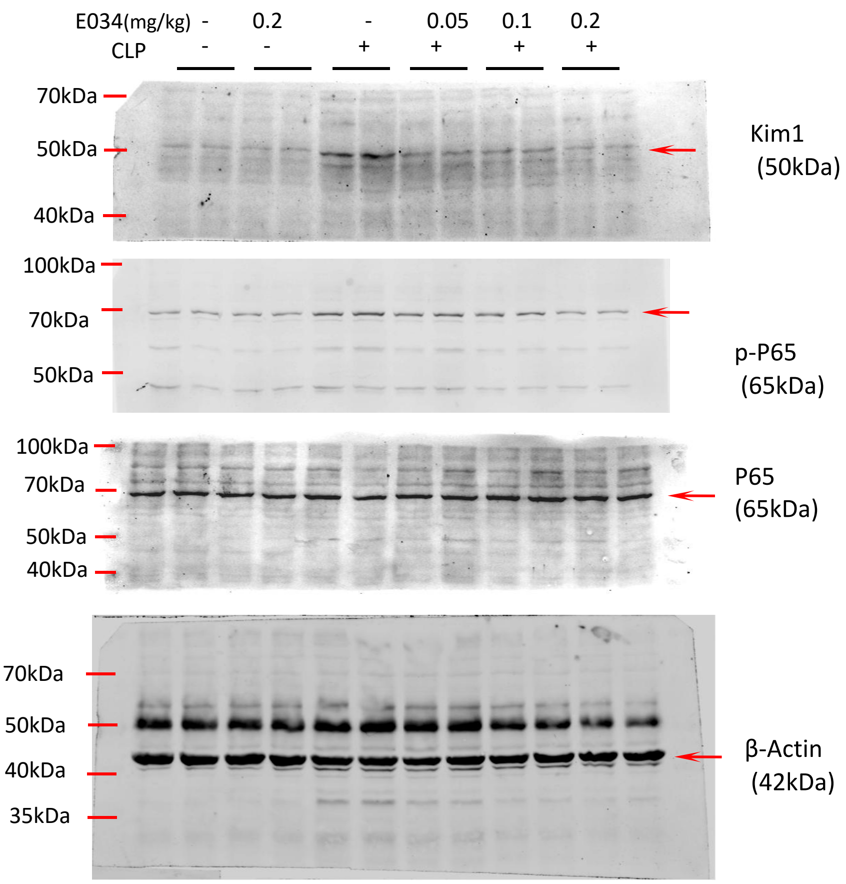

Fig.8q.

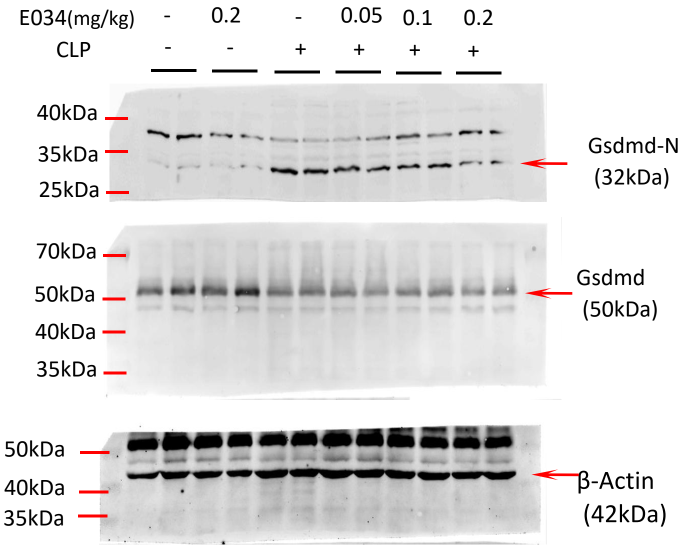

Fig.8s.

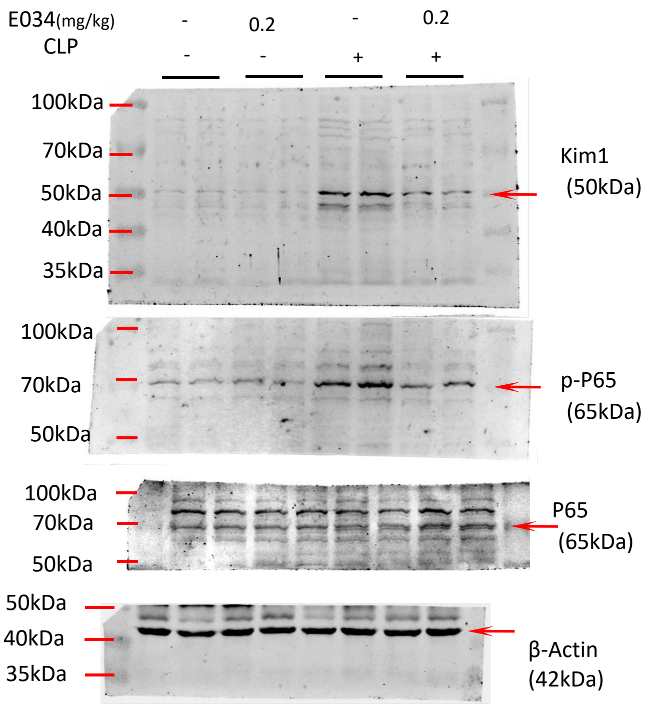

Fig.8t.

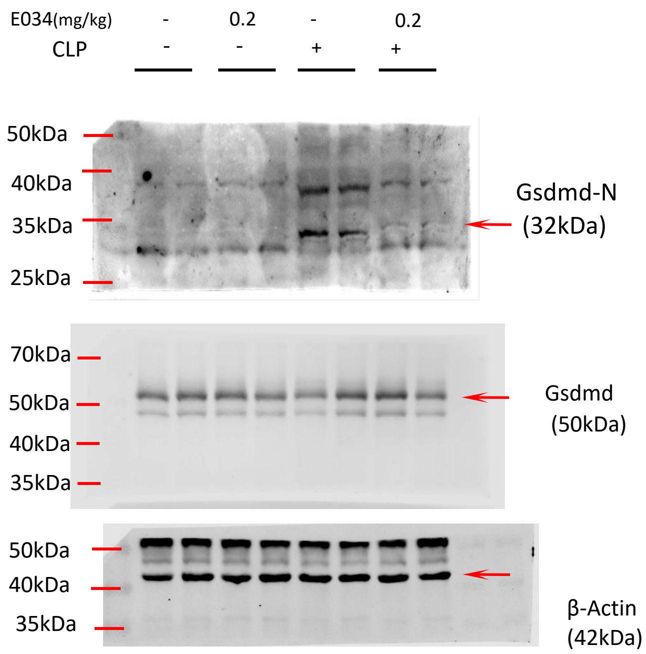

Fig.S1a

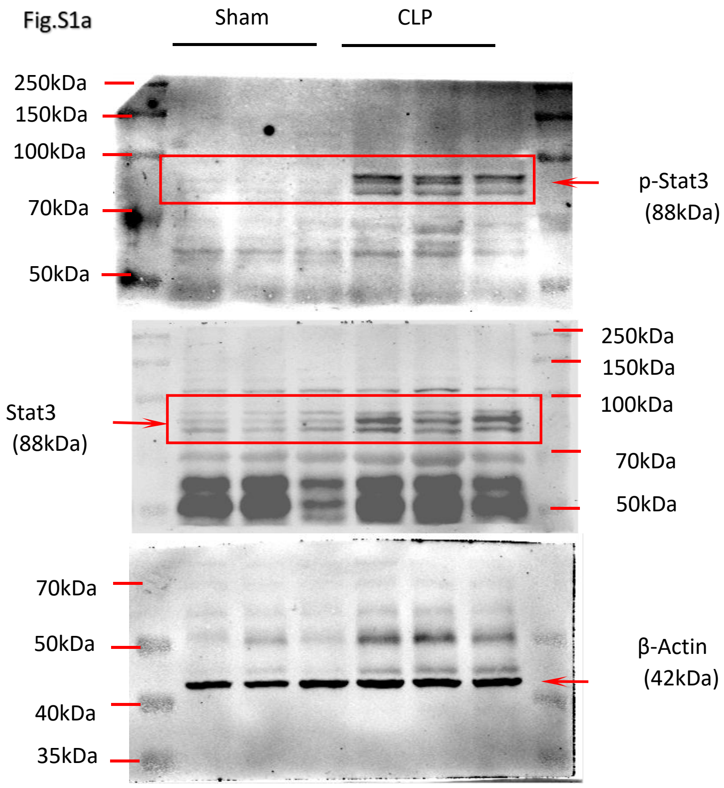

Fig.S1e

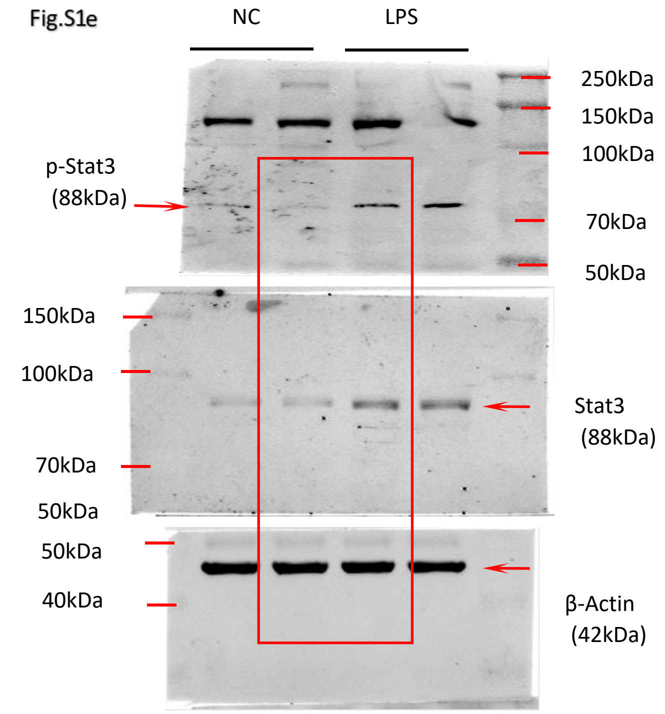

Fig.S1b

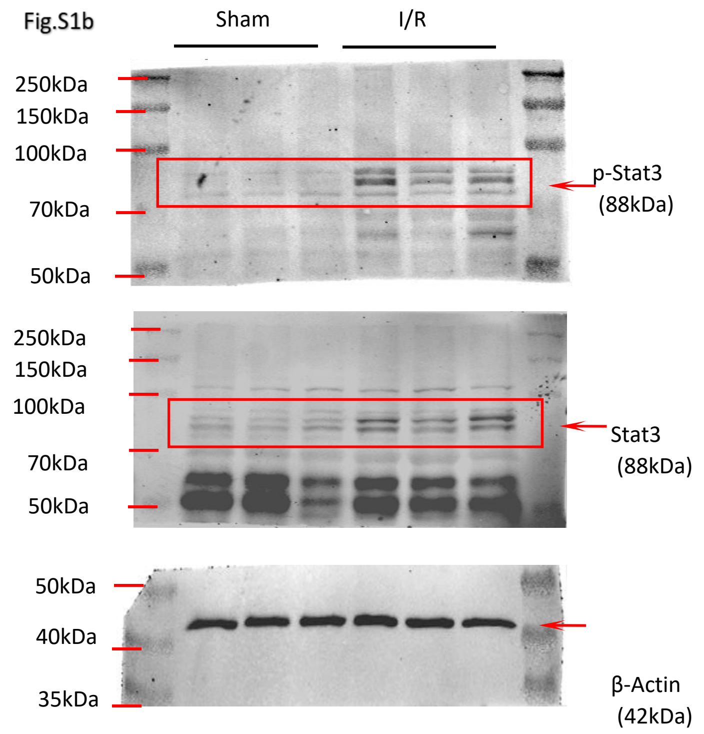

Fig.S1f

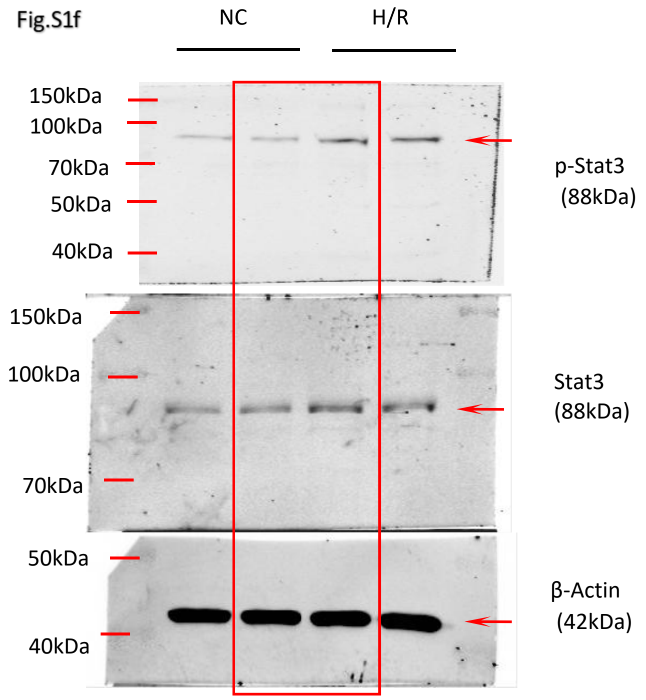

Fig.S2a.

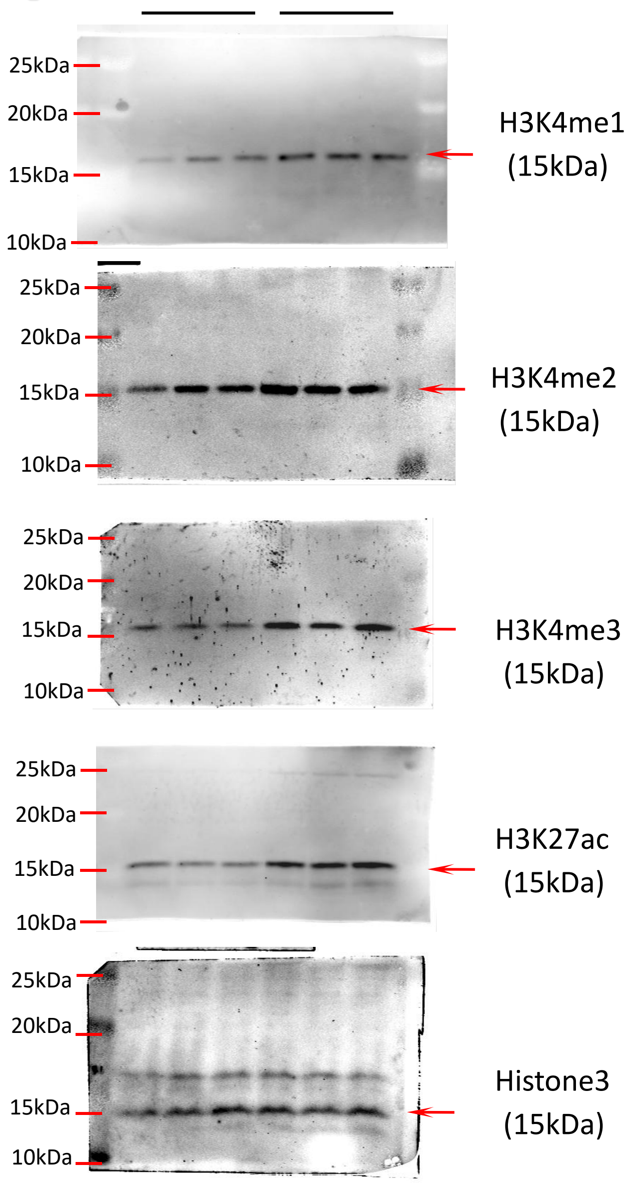

Fig.S2b.

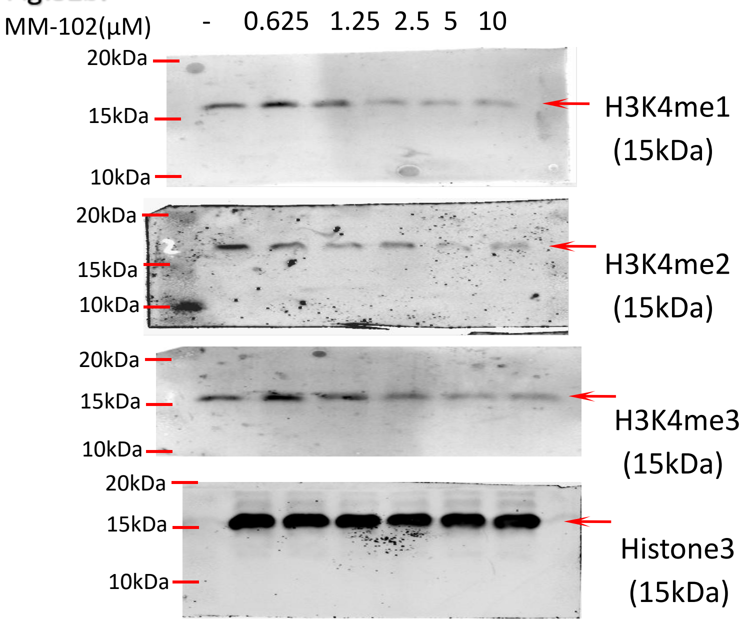

Fig.S2e.

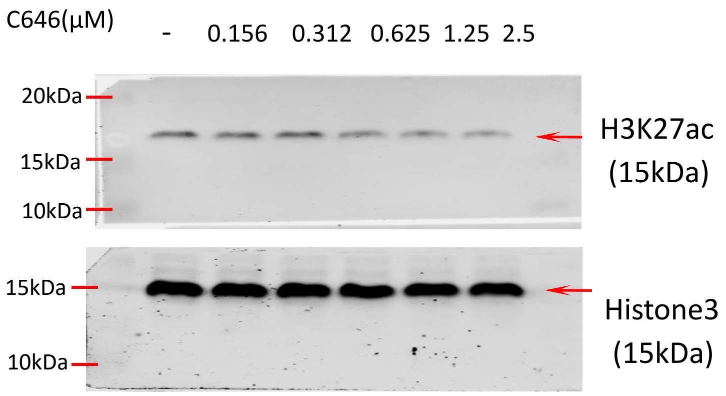

Fig.S3b.

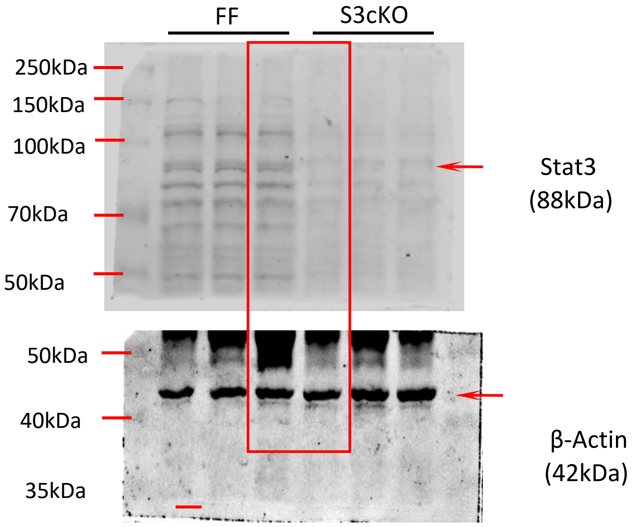

Fig.S3g.

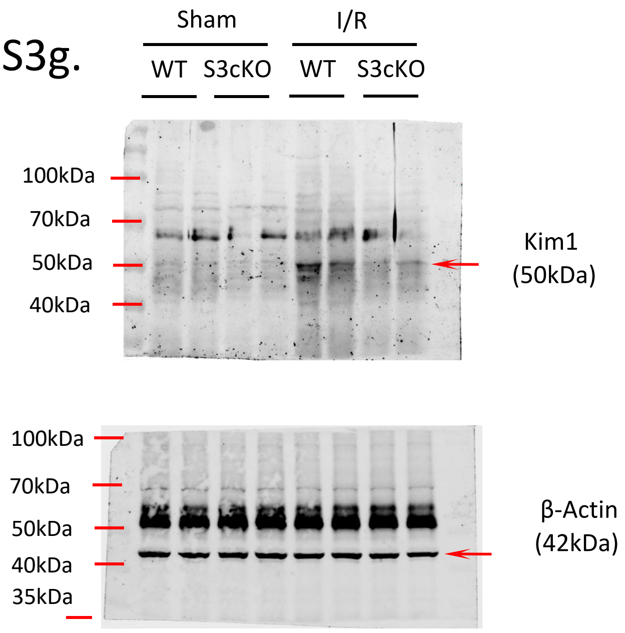

Fig.S3l.

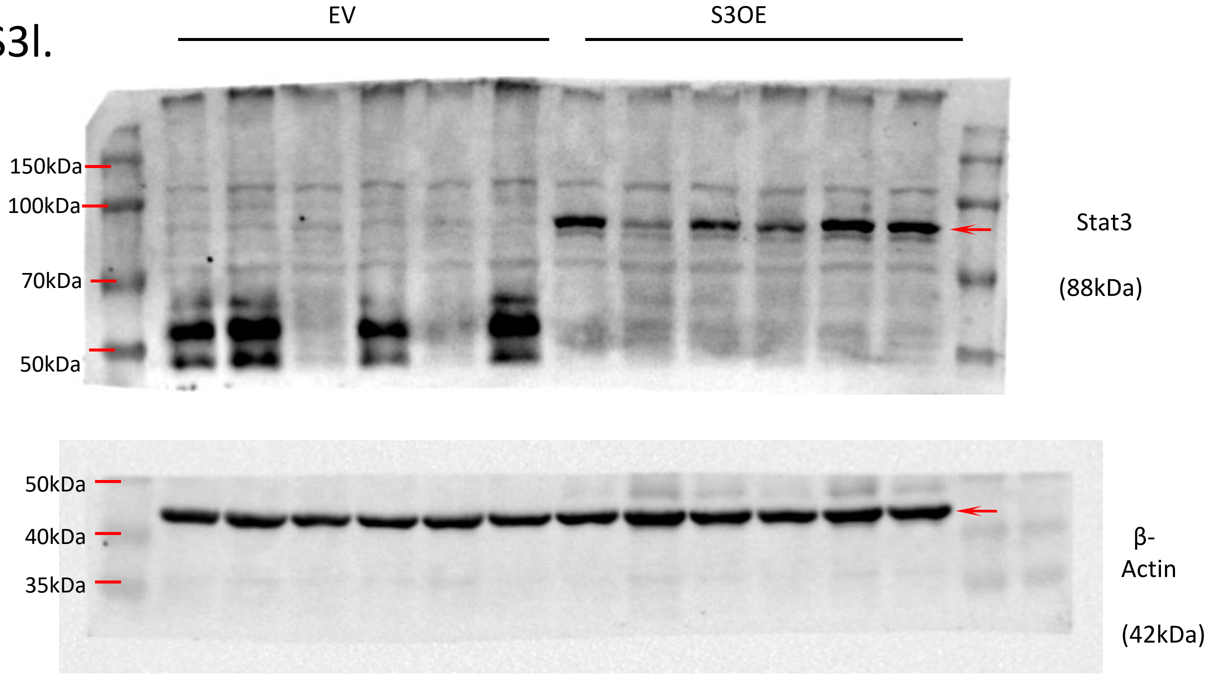

Fig.S3r.

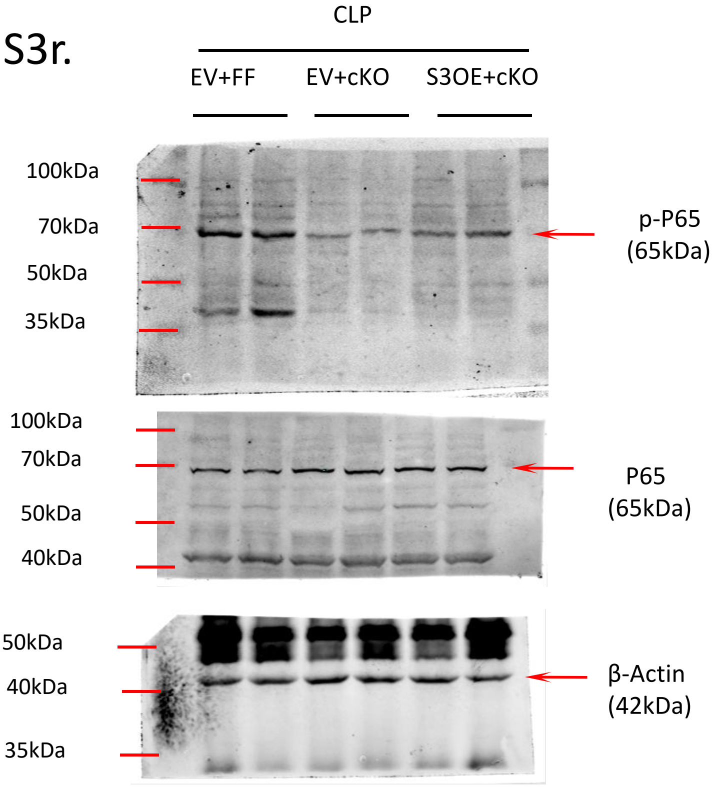

Fig.S4b.

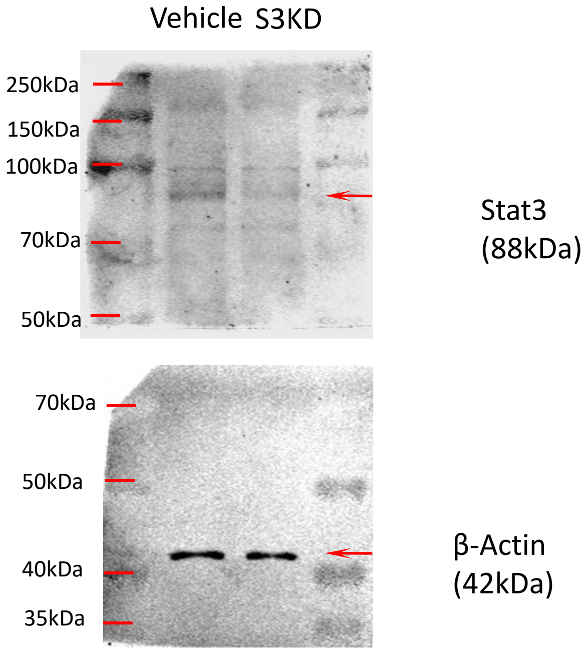

Fig.S4e.

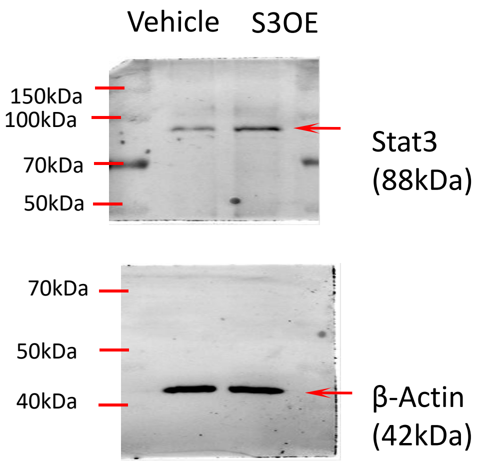

Fig.S5a.

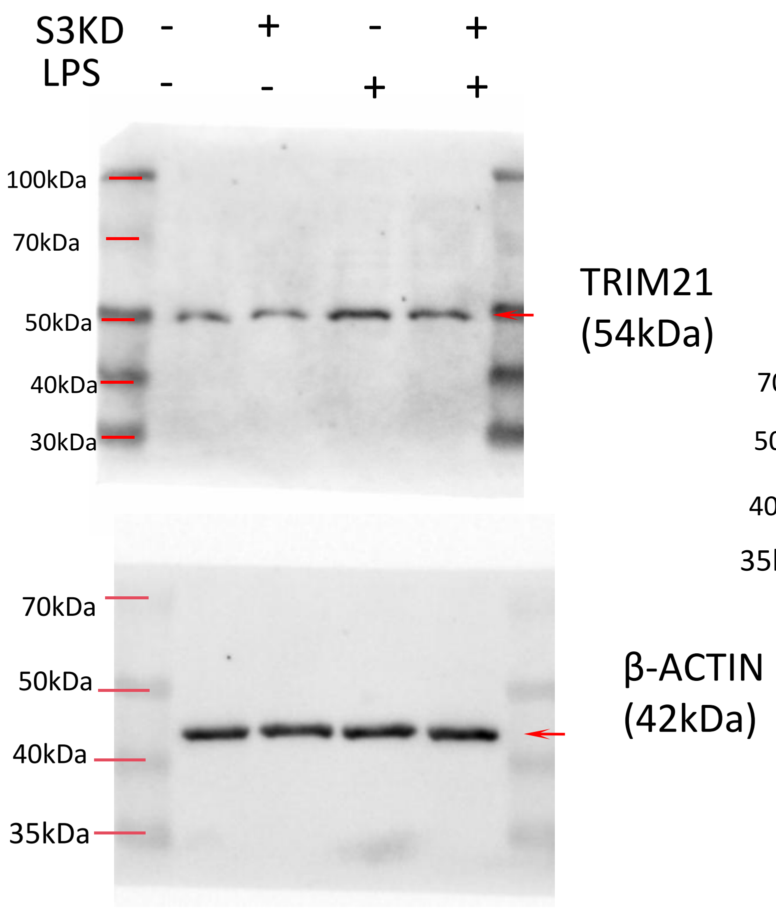

Fig.S5b.

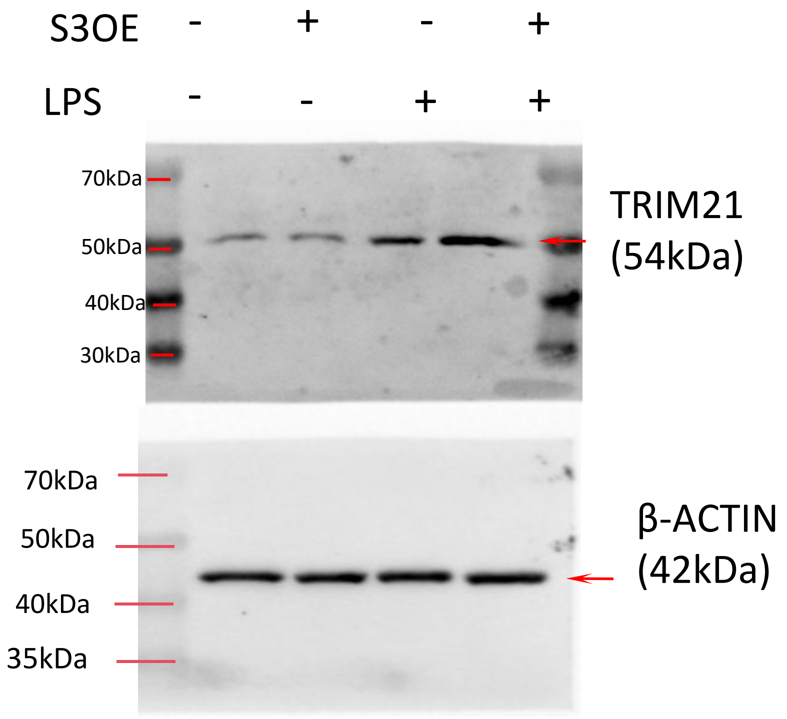

Fig.S6b.

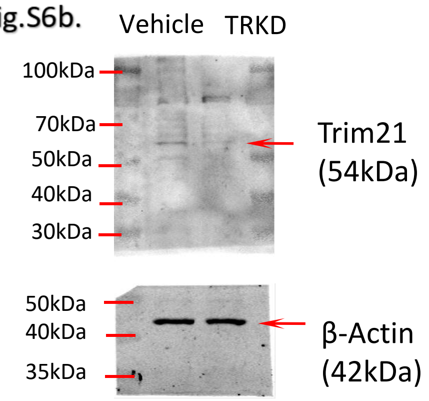

Fig.S6c .

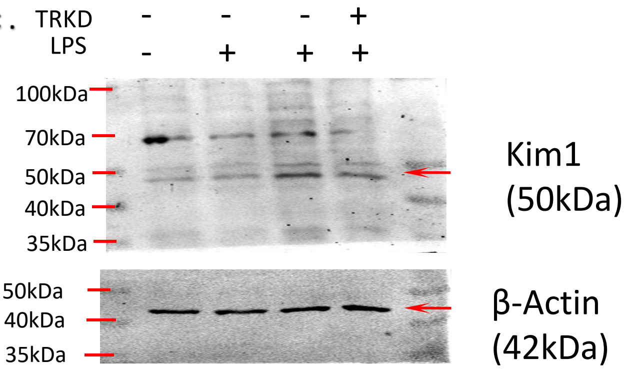

Fig.S6e.

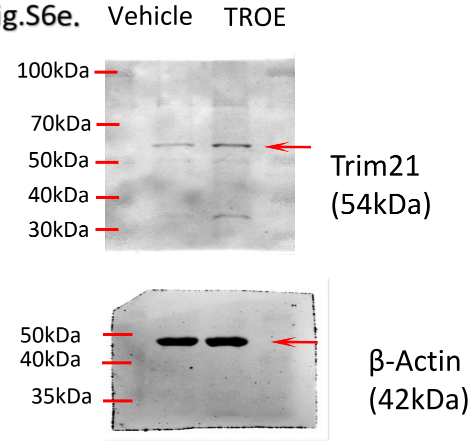

Fig.S6f.

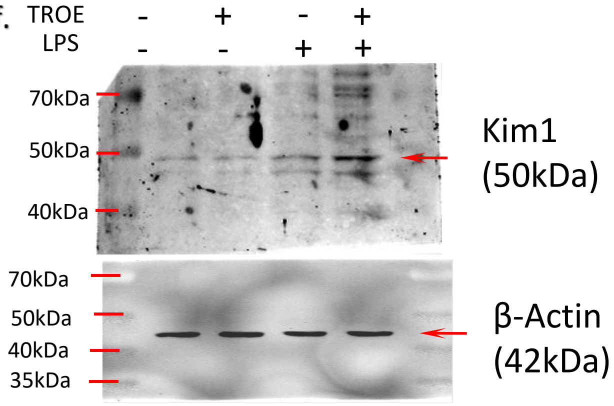

Fig.S6i.

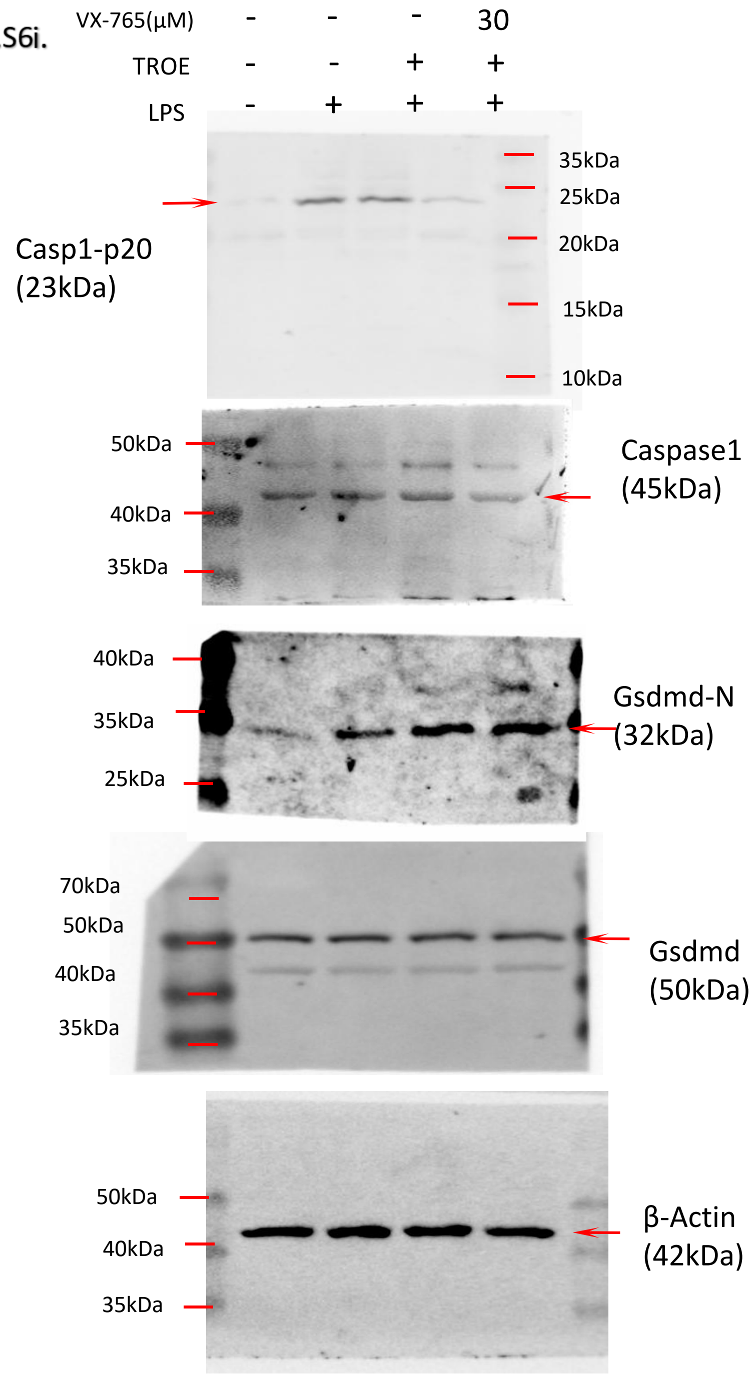

Fig.S6h.

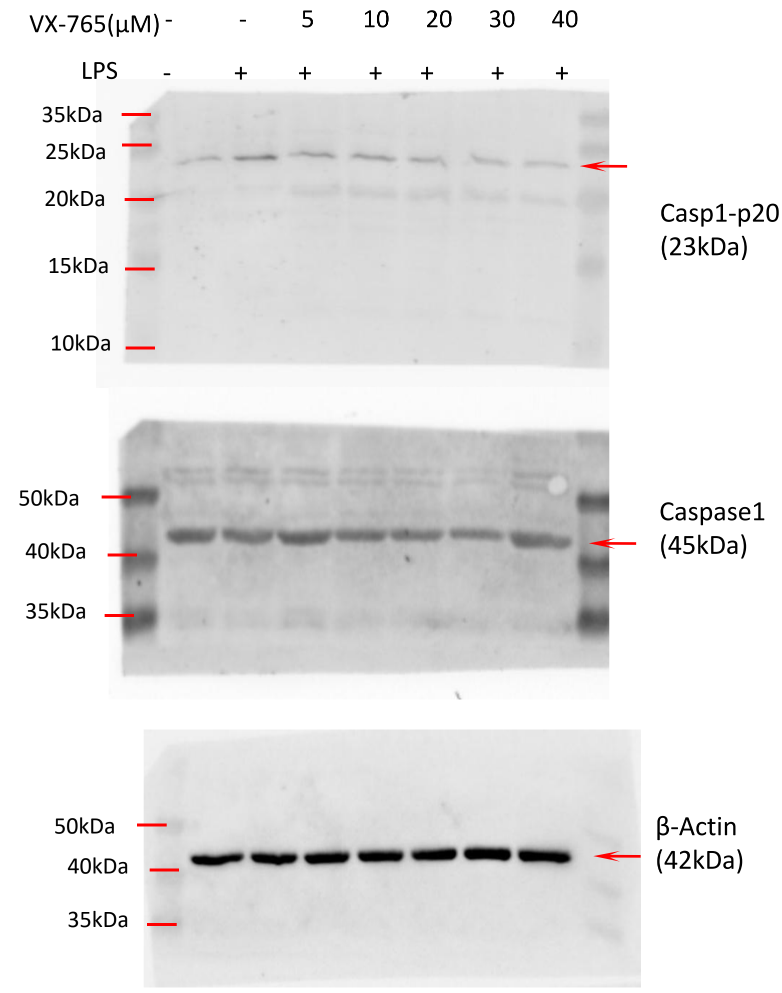

Fig.S7c .

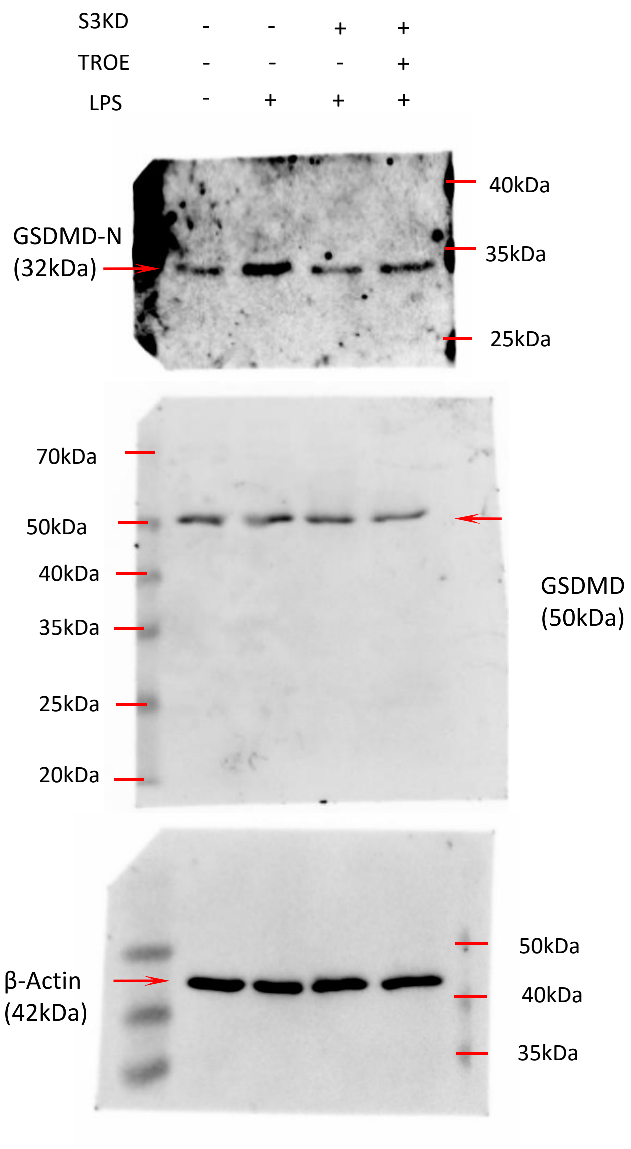

Fig.S7f .

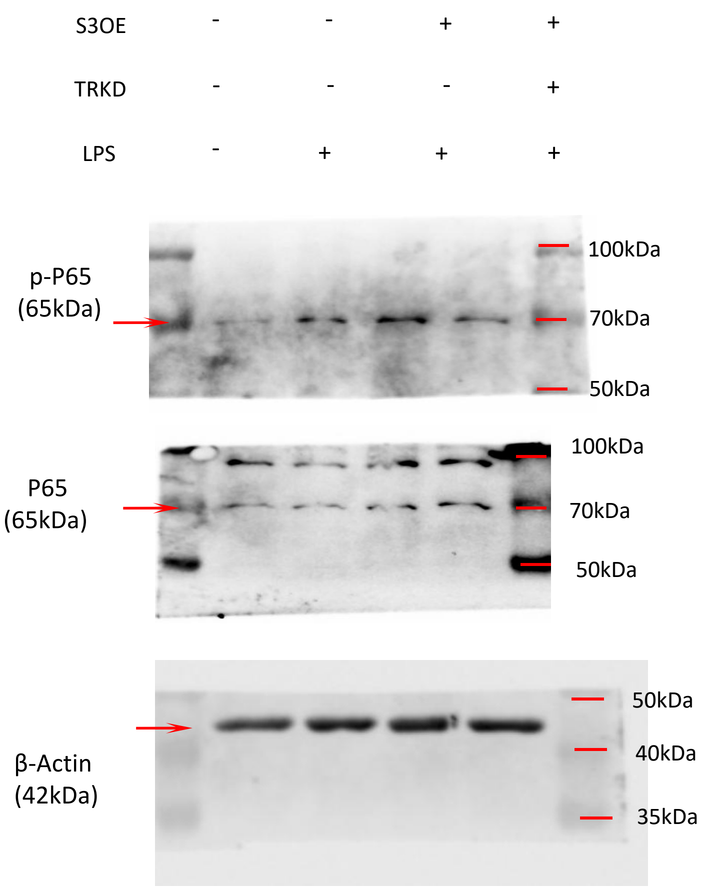

Fig.S8b.

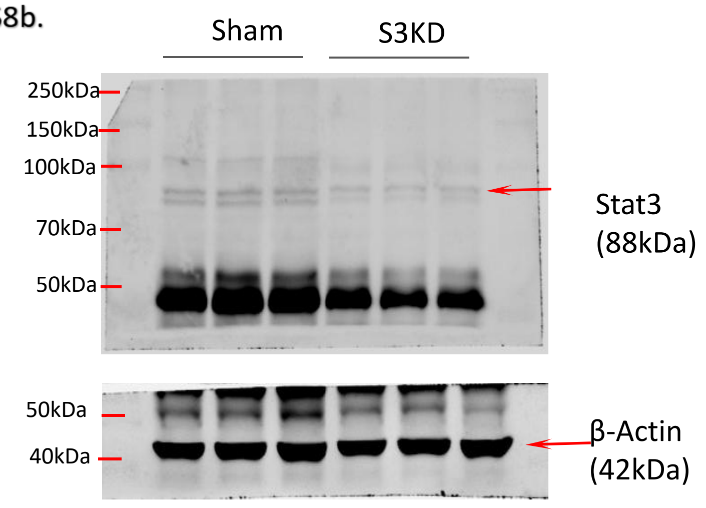

Fig.S8e.

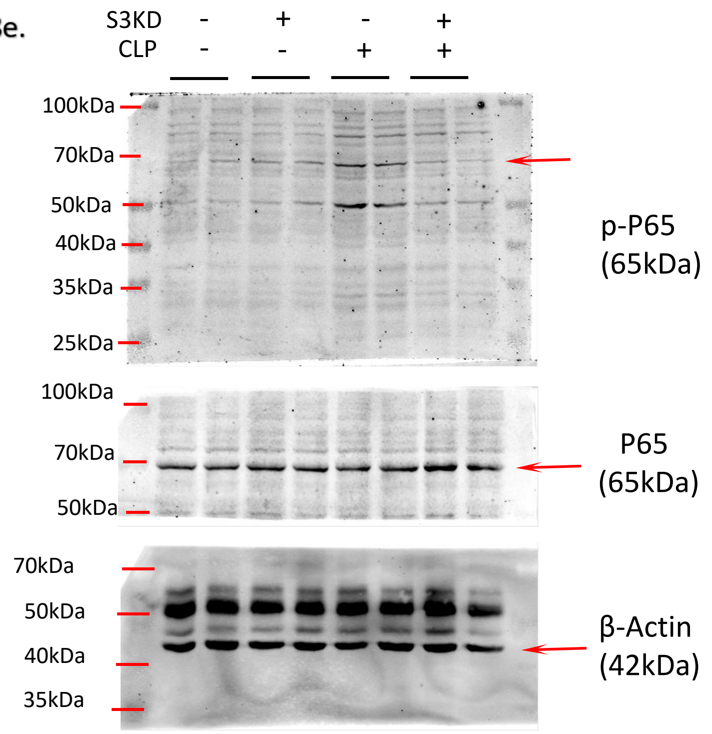

Fig.S11b.

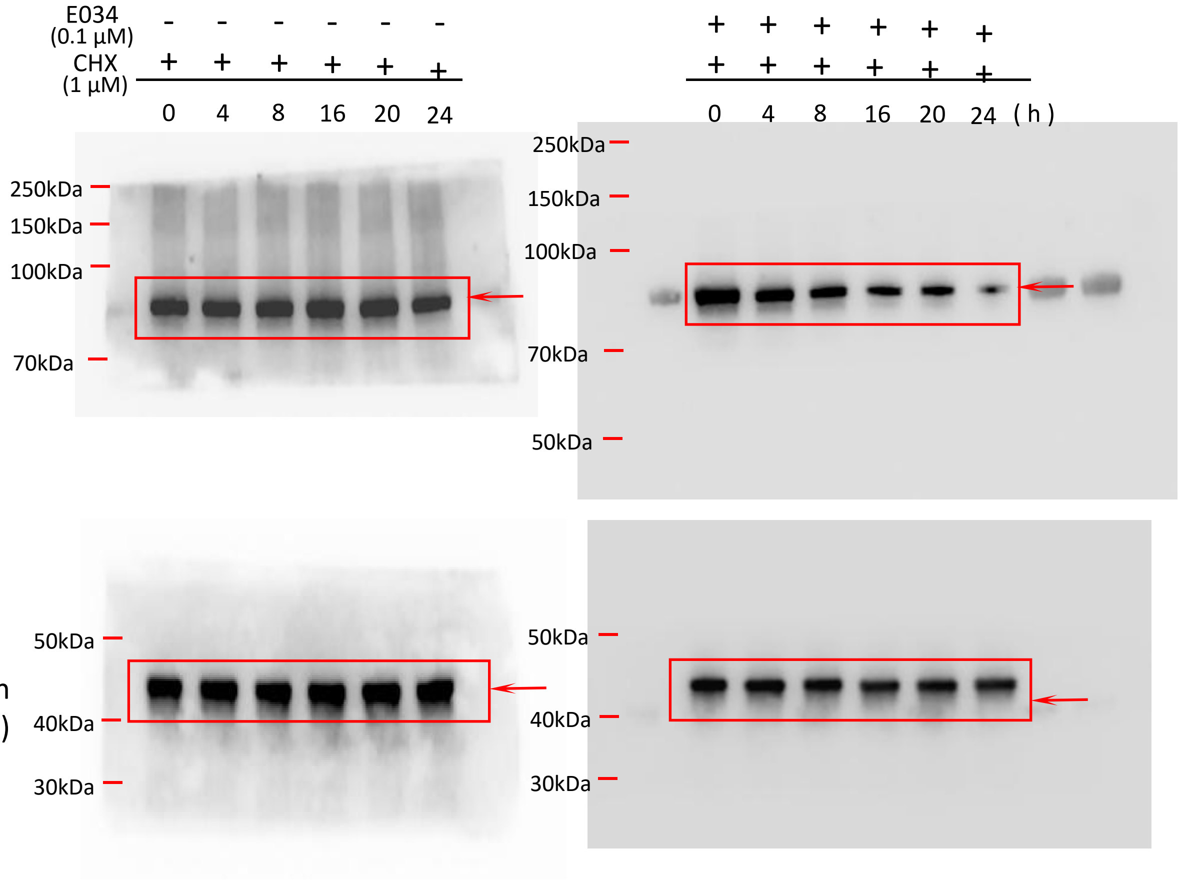

Fig.S11c.

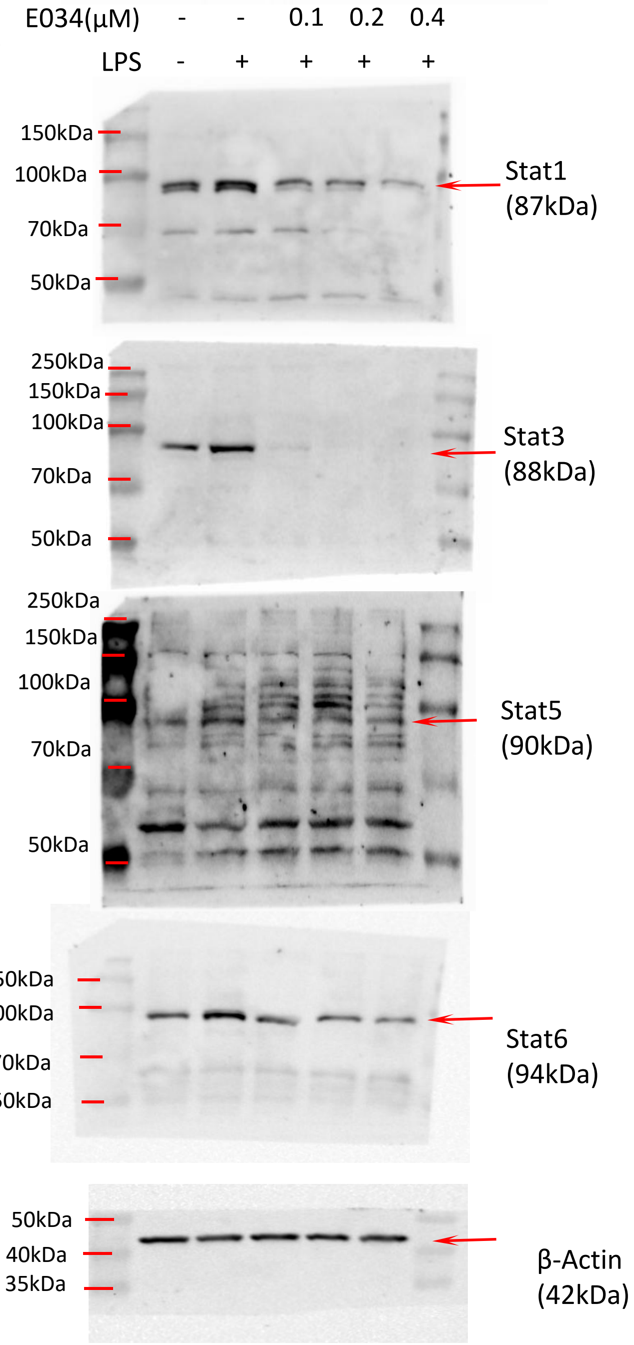

Fig.S11f.

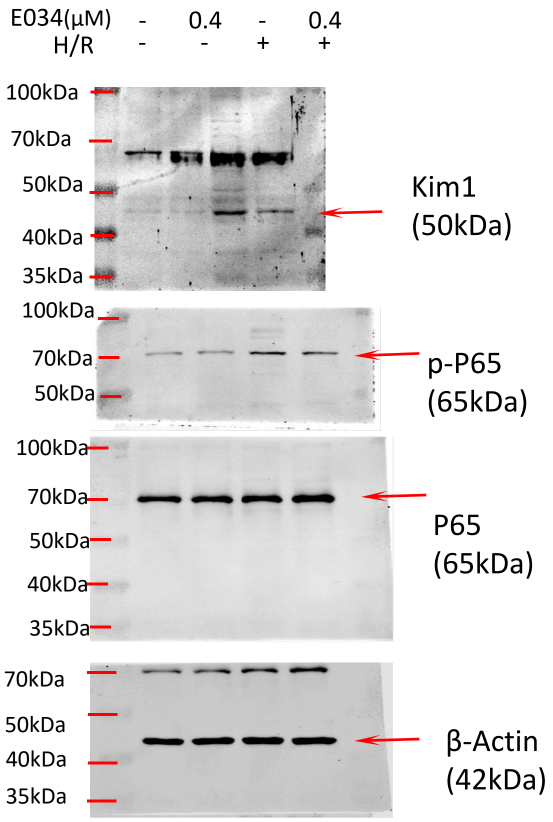

Fig.S12b.

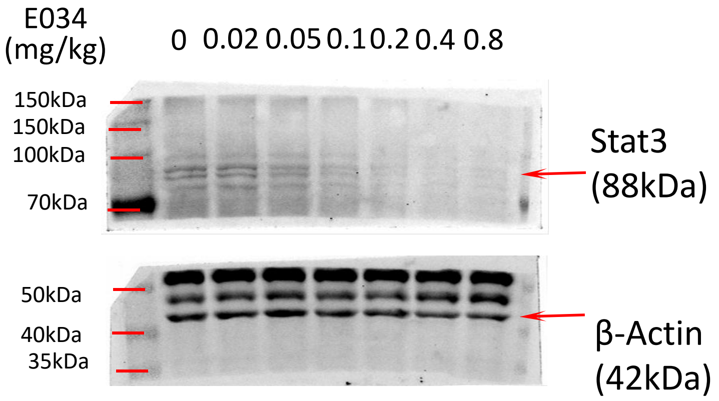
E034
(mg/kg)
0
0.02
0.05
0.1
0.2
0.4
0.8
150kDa
150kDa
100kDa
70kDa
Stat3
(88kDa)
50kDa
40kDa
35kDa
β-Actin
(42kDa)
