# Supplementary material for: Targeting Stat3 with conditional knockout or PROTAC technology alleviates renal injury by Limiting pyroptosis
Source: eBioMedicine. 2025 May 8;116:105739. doi: 10.1016/j.ebiom.2025.105739 (PMC12136849; doi:10.1016/j.ebiom.2025.105739)
Supplement: Reagent Validation file [file mmc8.docx]

**Reagent Validation Statement**

All reagents used in this study, including antibodies and cell lines, were commercially sourced. Antibody information, including catalogue numbers, vendors and Research Resource Identifier (RRID), is provided in Table S1. The full Western blot membranes for antibody specificity detection are shown in Figure S16 - S23. The detailed documentation of reagent validation is available in Table S2. Source Information for Model Organisms are shown in Table S3. Cell lines were authenticated through STR profiling, and the STR results are included in Table S4 and file “mTECs STR Identification” and “HK-2 Cell STR Identification”. The shRNA and siRNA sequences used are shown in Table S5. The primer sequences used are shown in Table S6.

**Table S1.** **Antibodies for western blot, IF/IHC, ChIP assay**

| **Antibody** | **Clone** | **Dilution** | **Manufacturer** | **Cat#** | **RRID** |
| --- | --- | --- | --- | --- | --- |
| Anti–Stat3 | 5H4 | WB: 1:1000；IF/IHC: 1:200 | Bioss | bsm-33223M | AB_3676445 |
| Anti-Phospho-Stat3 | Monoclonal | WB: 1:1000；IF/IHC: 1:200；ChIP: 1:50 | CST | #9145 | AB_2491009 |
| Anti-F4/80 | Clone D2S9R | IF/IHC: 1:200 | CST | #70076 | AB_2799771 |
| Anti-Histone H3 | C200234 | WB: 1:1000；ChIP: 1:50 | CST | #4620 | AB_1904005 |
| Anti-Trim21 | Polyclonal | WB: 1:1000；IF: 1:200 | Proteintech | 12108-1-AP | AB_2209469 |
| Anti-H3K4me1 | Polyclonal | WB: 1:1000 | ABclonal | A2355 | AB_2764315 |
| Anti-H3K4me2 | Polyclonal | WB: 1:1000 | ABclonal | A2356 | AB_2764316 |
| Anti-H3K4me3 | Polyclonal | WB: 1:1000 | ABclonal | A22146 | AB_3066542 |
| Anti-H3K27Ac | Monoclonal | WB: 1:1000; ChIP: 1:50 | ABclonal | A7253 | AB_2767797 |
| Anti-Caspase-1 | 14F468 | IB: 1:200 | Santa Cruz Biotechnology | sc-56036 | AB_781816 |
| Anti-Caspase-1(p20) | ARC60705 | WB: 1:1000 | ABclonal | A23429 | AB_3683536 |
| Cleaved Caspase-1 (Asp296) | Monoclonal | WB: 1:1000 | CST | 89332 | AB_2923067 |
| Anti-P300 | NM11 | ChIP: 1:20 | Santa Cruz Biotechnology | sc-32244 | AB_628076 |
| Anti-CBP | G-8 | ChIP: 1:20 | Santa Cruz Biotechnology | sc-365387 | AB_10846188 |
| Anti-Tim1(Kim1) | JE39-41 | WB: 1:1000；IF: 1:200 | HUABIO | HA721535 | AB_3072651 |
| Anti-P65 | SZ10-04 | WB: 1:1000 | HUABIO | ET1603-12 | AB_3069668 |
| Anti-P65 | Monoclonal | WB: 1:1000 | CST | 8242t | AB_10859369 |
| Anti-p-P65 | SP07-00 | WB: 1:1000 | HUABIO | ET1604-27 | AB_3069692 |
| Anti-p-P65 | Monoclonal | WB: 1:1000 | CST | 3033t | AB_331284 |
| Anti-gasdermin D | A8D7 | WB: 1:1000 | HUABIO | HA601046 | AB_3073484 |
| Anti-gasdermin D (N terminal) | Polyclonal | WB: 1:1000; IF: 1:200; IB: 1:1000 | HUABIO | ER1901-37 | AB_3069329 |
| Anti-gasdermin D (N terminal) | Polyclonal | WB: 1:1000 | SAB | 55651 | AB_3683529 |
| Anti-Nlrp3 | SC06-23 | IB: 1:1000 | HUABIO | ET1610-93 | AB_3069980 |
| Anti-β-actin | Polyclonal | WB: 1:1000 | HUABIO | R1207-1 | AB_3073201 |
| Anti-Stat1 | Monoclonal | WB: 1:1000 | Diagbio | db12458 | AB_3677655 |
| Anti-Stat5 | JJ08-78 | WB: 1:1000 | HUABIO | ET1701-45 | AB_3070213 |
| Anti-Stat6 | SY13-09 | WB: 1:1000 | HUABIO | ET1605-49 | AB_3069716 |
| IRDye® 800CW Goat anti-Mouse IgG Secondary Antibody | Polyclonal | WB: 1:10000 | LICORBio | P/N: 925-32210 | AB_621842 |
| IRDye® 800CW Goat anti-Rabbit IgG Secondary Antibody | Polyclonal | WB: 1:10000 | LICORBio | P/N: 926-32211 | AB_621843 |
| Lotus Tetragonolobus Lectin (LTL), Fluorescein | / | IF: 1:200 | Vectior | FL-1321-2 | AB_2336559 |
| Anti-Calbindin | / | IF: 1:200 | Servicebio | GB12268-100 | AB_3683528 |
| Dolichos Biflorus Agglutinin (DBA), Fluorescein | / | IF: 1:200 | Vectior | FL-1031 | AB_2336394 |

**
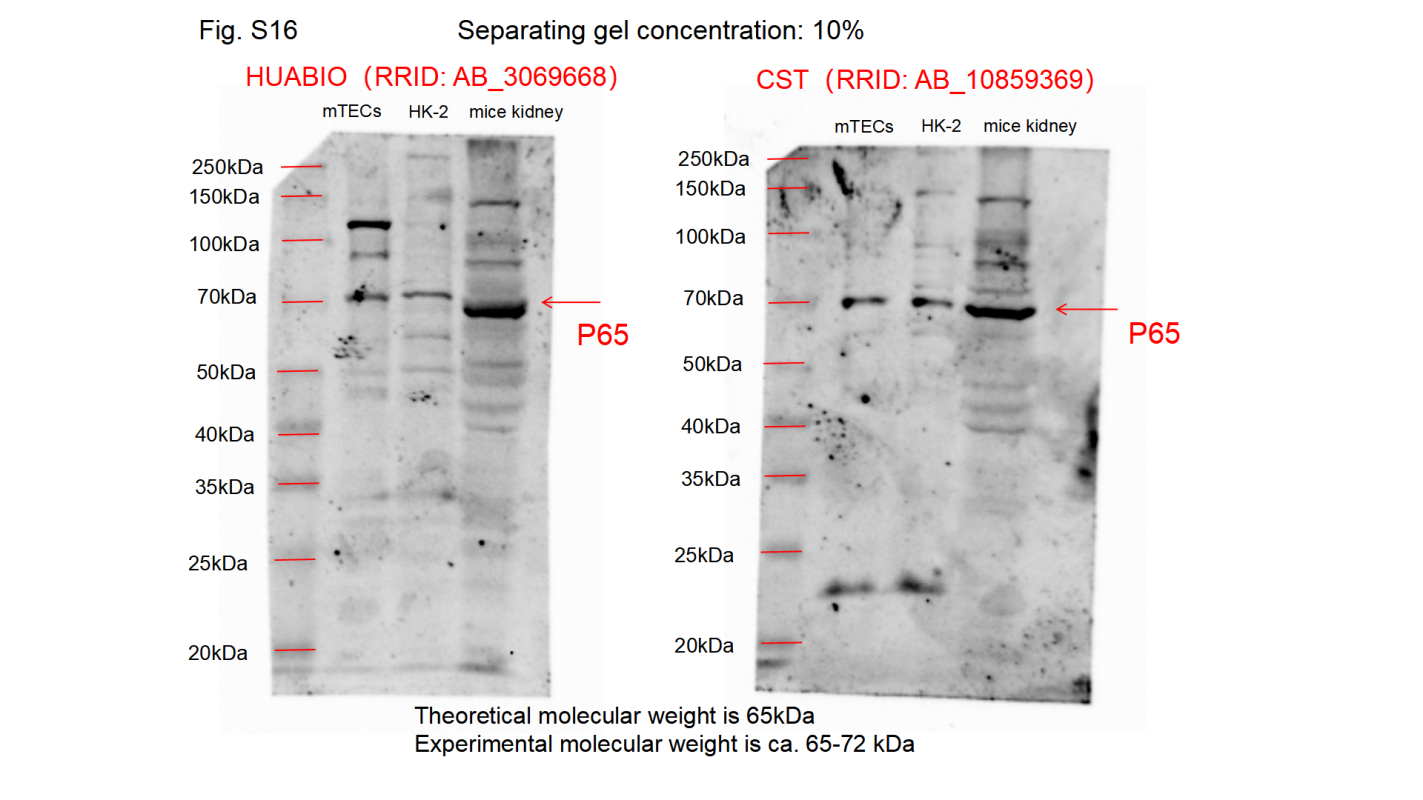
**

**Figure S16. The intact membrane dedicated to validating the specificity of P65 antibody.**

**
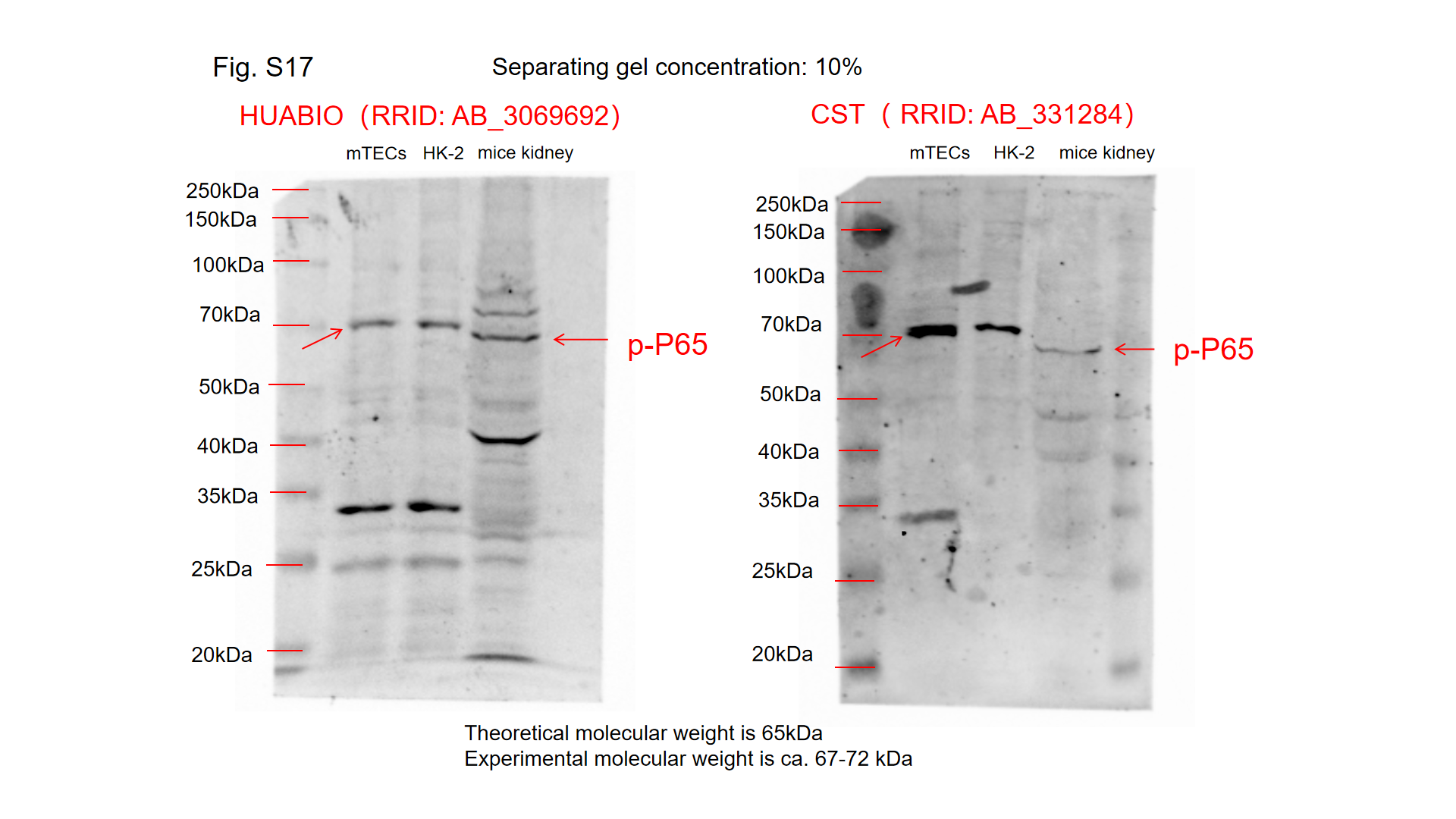
**

**Figure S17. The intact membrane dedicated to validating the specificity of p-P65 antibody.**

**
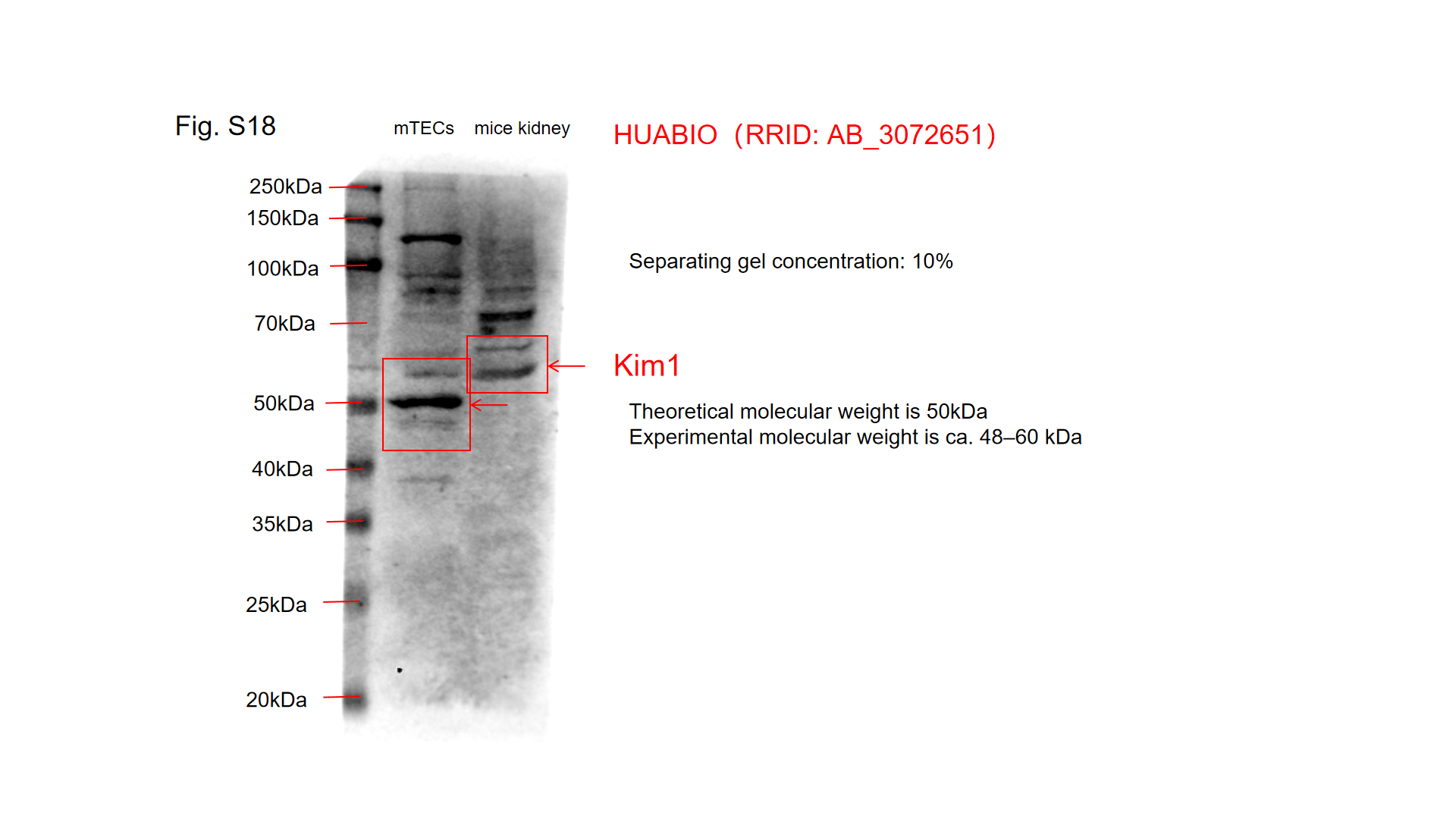
**

**Figure S18. The intact membrane dedicated to validating the specificity of Kim1 antibody.**

**
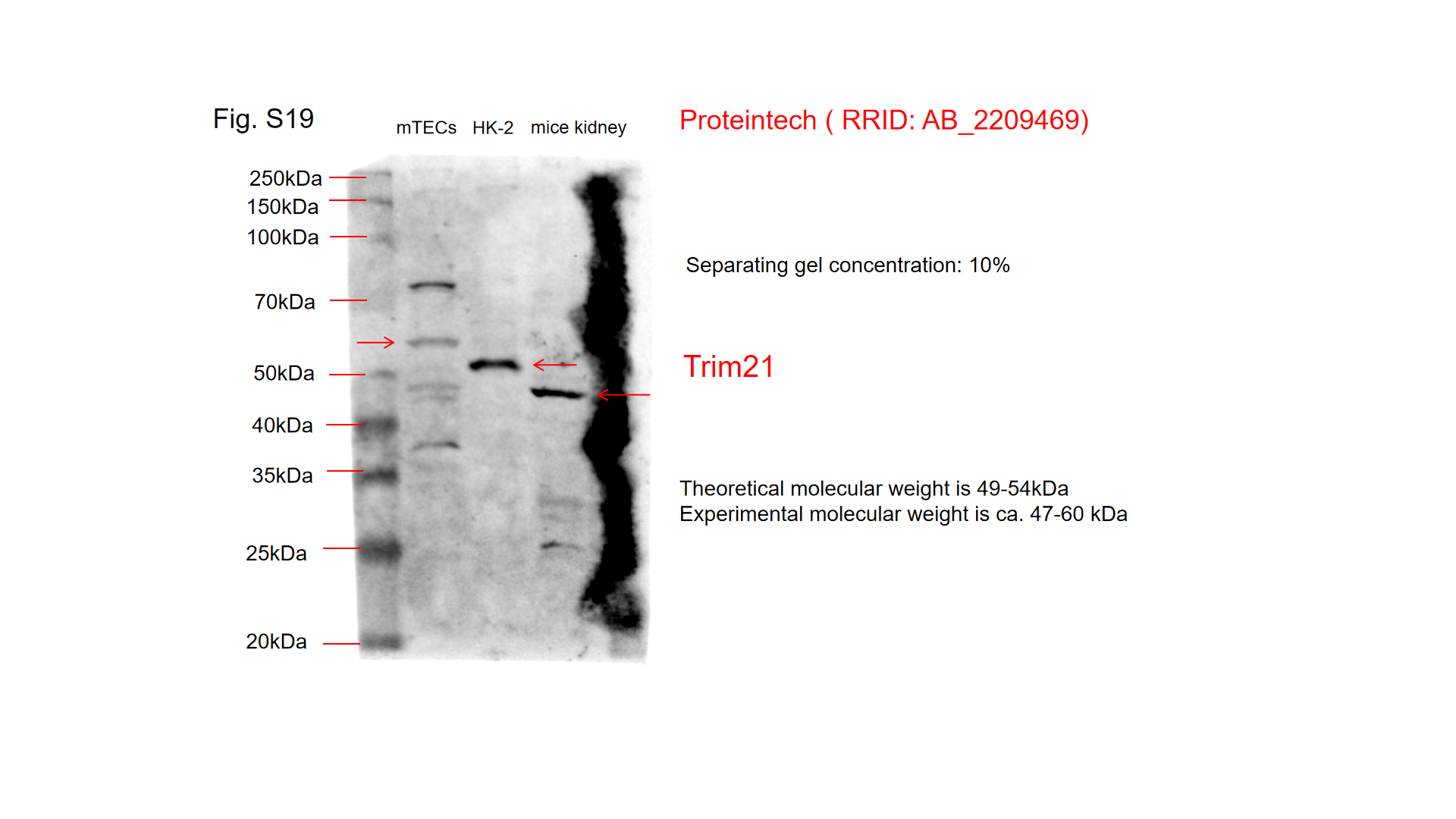
**

**Figure S19. The intact membrane dedicated to validating the specificity of Trim21 antibody.**

**
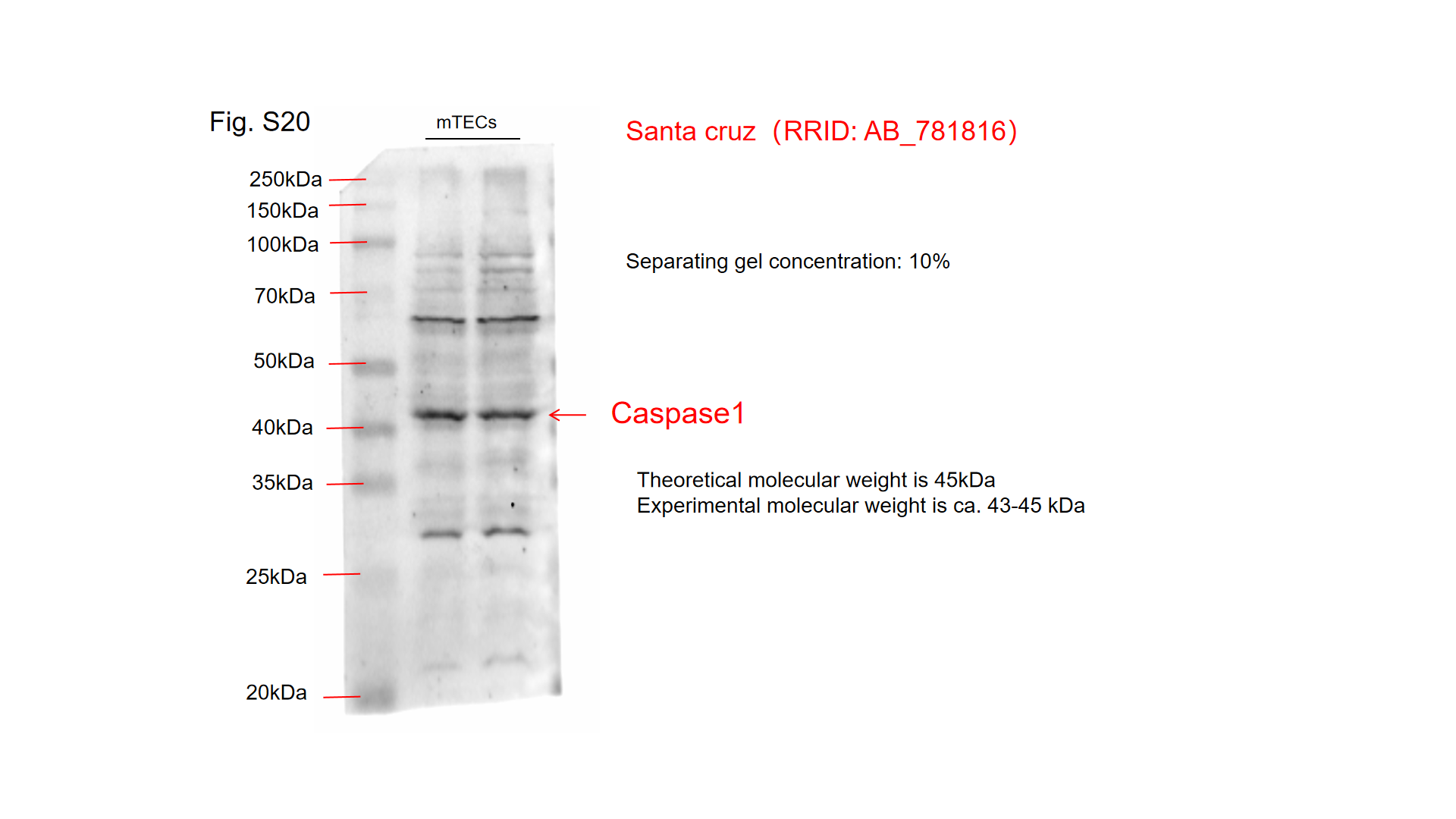
**

**Figure S20. The intact membrane dedicated to validating the specificity of Caspase1 antibody.**

**
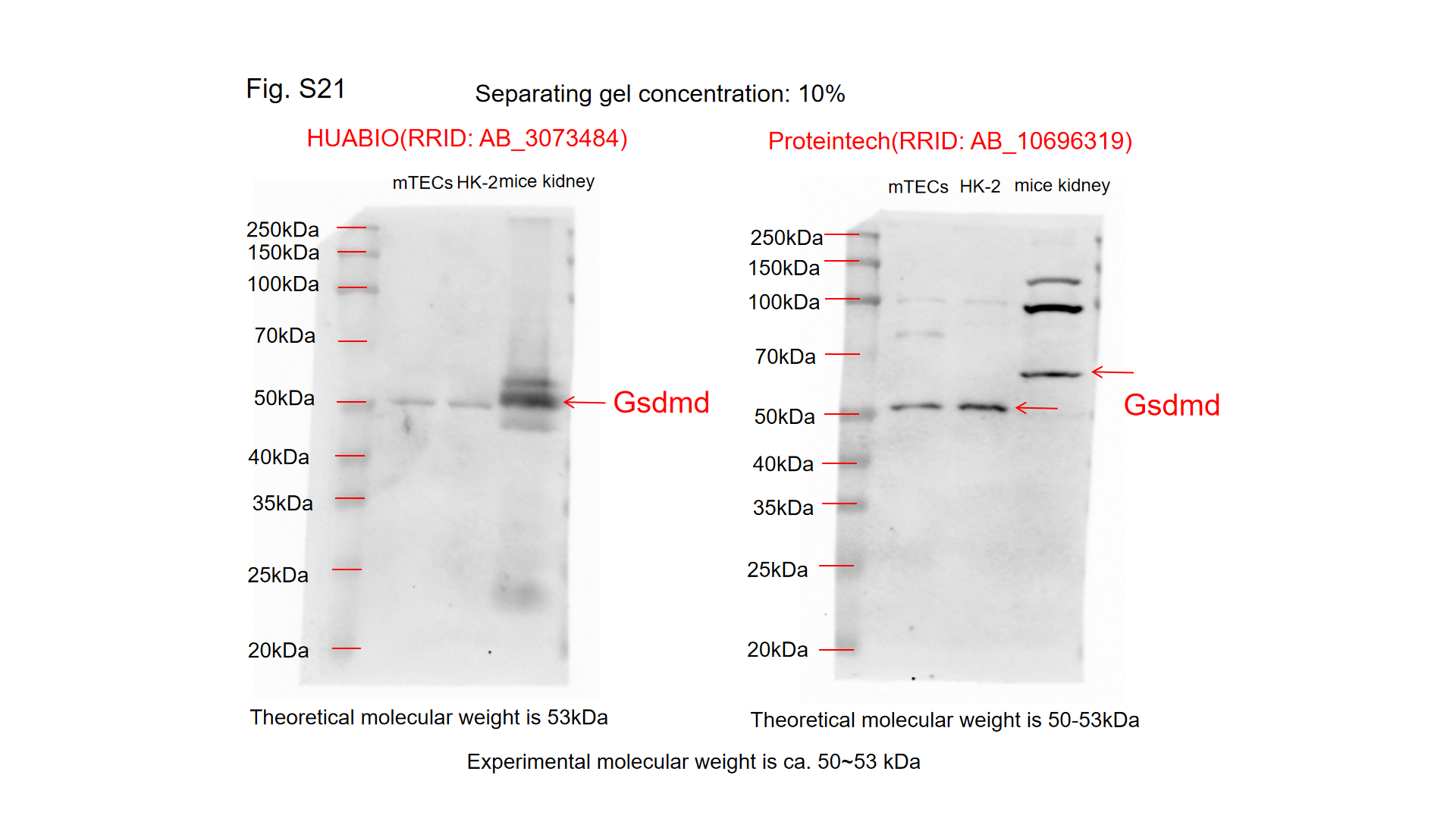
**

**Figure S21. The intact membrane dedicated to validating the specificity of Gsdmd antibody.**

**
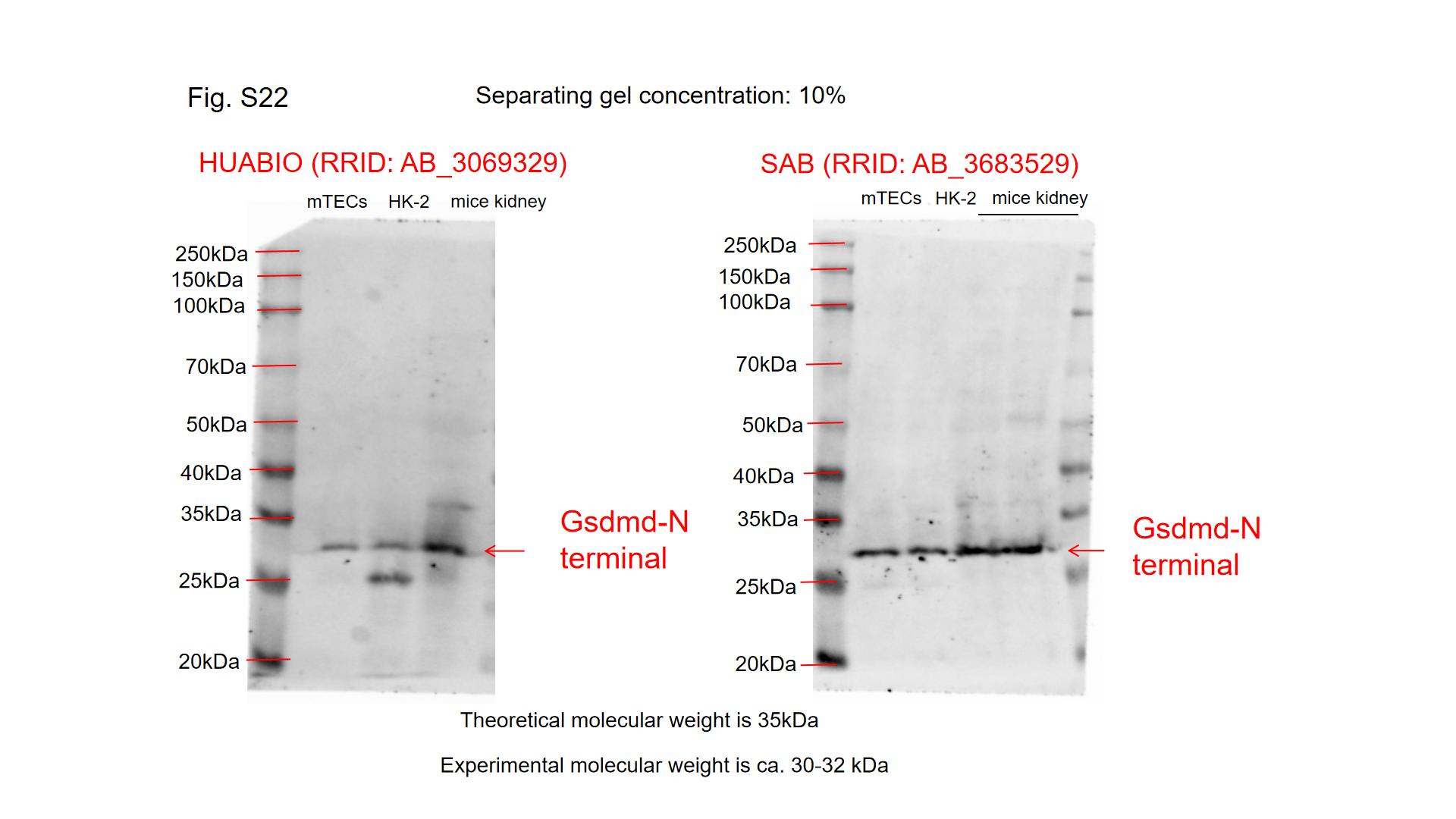
**

**Figure S22. The intact membrane dedicated to validating the specificity of Gsdmd-N antibody.**

**
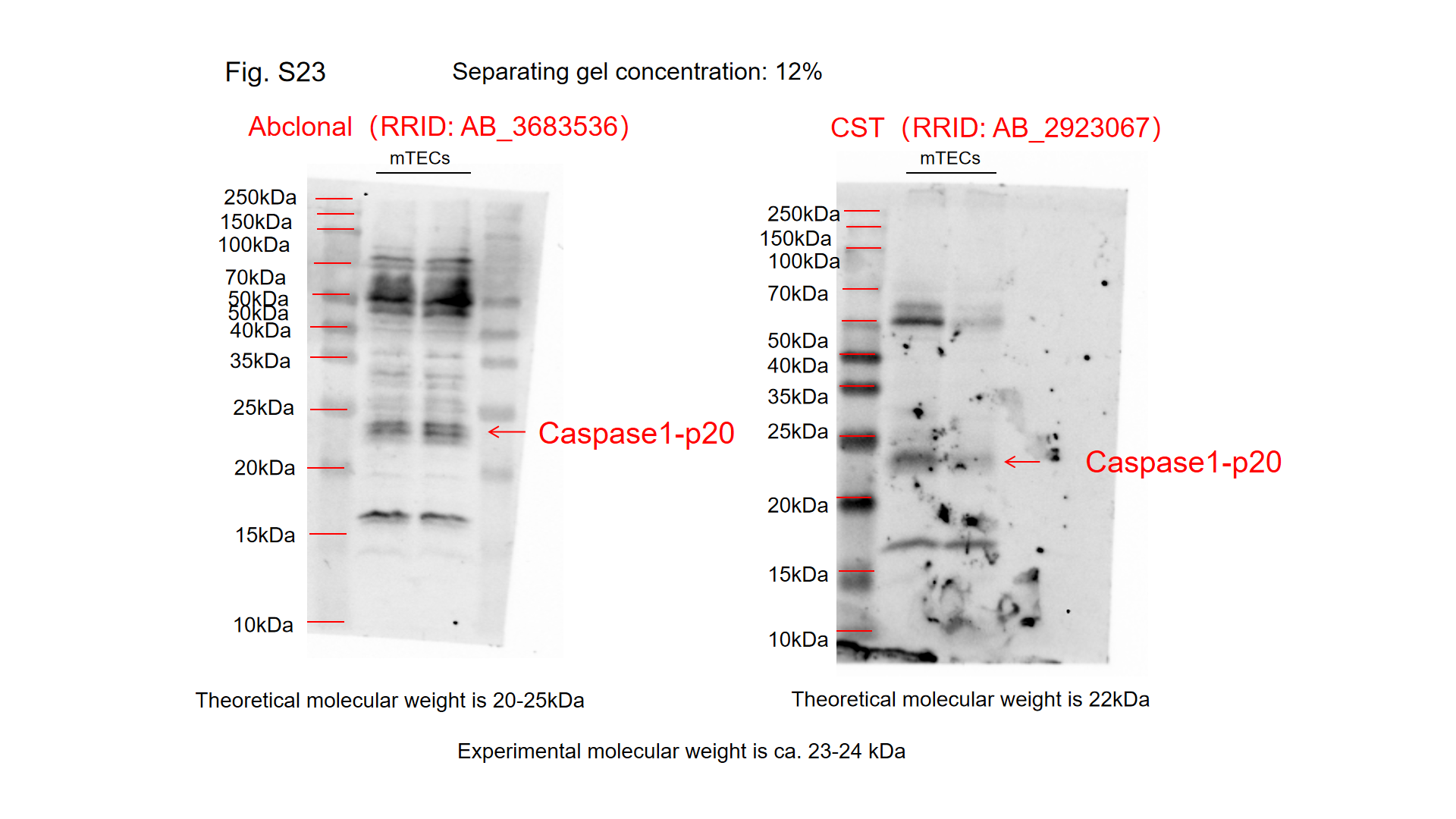
**

**Figure S23. The intact membrane dedicated to validating the specificity of Caspase-p20 antibody.**

**Table S2.** **Reagents used in the article**

| **Reagent** | **Manufacturer** | **Cat#** | **Other information** |
| --- | --- | --- | --- |
| Glycine | Solarbio | G8200 | CAS: 56-40-6 |
| Tris | Solarbio | T8060 | CAS: 77-86-1 |
| SDS | Solarbio | S8010 | CAS: 151-21-3 |
| LPS | Sigma-Aldrich | L2880 | CAS: 93572-42-0 |
| C646 | MCE | HY-13823 | CAS: 328968-36-1 |
| VX-765 | MCE | HY-13205 | CAS: [273404-37-8](https://www.medchemexpress.cn/cas/273404-37-8.html) |
| MM-102 | APExBIO | B1582 | CAS: 1417329-24-8 |
| DMSO | Solarbio | D8371 | CAS: 67-68-5 |
| MTT (Thiazolyl Blue) | MCE | HY-15924 | CAS: 298-93-1 |
| Mouse Il-1β ELISA Kit | Epizyme Biotech | HJ177 | RRID: AB_3676447 |
| Mouse Il-18 ELISA Kit | Elabscience | E-EL-M0730 | RRID: AB_3676448 |
| DMEM/F12 Cell Medium | ZWK | A-DF03 | / |
| DMEM Cell Medium (Low Sugar) | ZWK | A-D01D | / |
| Fetal bovine serum | Wisent | 086-150 | / |
| Trypsin-EDTA Solution | beyotime | C0201-100ml | / |
| PBS | Servicebio | G4202-500ML | / |
| Universal two-step test kit Mouse/Rabbit enhanced polymer test system | ZSGB-BIO | PV-9000 | / |
| RIPA Lysis Buffer (Strong) | beyotime | P0013B | / |
| Protease Phosphatase Inhibitor Mixture | beyotime | P1045 | / |
| Three-color Prestain Protein Marker | Ya Enzyme | WJ103 | / |
| PAGE Gel Fast Preparation Kit (12%) | Ya Enzyme | PG113 | / |
| PAGE Gel Fast Preparation Kit (10%) | Ya Enzyme | PG112 | / |
| Western Primary Antibody Dilution Buffer | beyotime | P023A-500ml | / |
| AG RNAex Pro Reagent | AG Bio | AG21102 | / |
| Nuclease-free water | biosharp | BL510B | / |
| 5X Evo M-MLV RT Master Mix *1 | AG Bio | AG11706 | / |
| 2X SYBR Green Pro Taq HS Premix* | AG Bio | AG11701 | / |
| 20×Tris-EDTA Antigen repair solution (pH8.0) | Servicebio | G1206-250ML | / |
| SimpleChIP(R)Kit(Magnetic Beads) 4C＆RT Reagents | CST | 91820S | / |
| NP40 lysis buffer | Beyotime | P0013F | / |
| Protein A/G magnetic beads | MCE | HY-K0202 | / |
| Universal Tissue Fixative (Neutral) | Servicebio | G1101-500ML | / |
| Anti-fluorescence quenching sealing solution | Beyotime | P0126-5ml | / |
| PAS assay kit | Solarbio | G1281 | / |
| Hematoxylin-Eosin(HE) Stain Kit | Solarbio | G1120 | / |
| CRE assay kits | Nanjing Jiancheng Bioengineering Institute | C011-2-1 | / |
| BUN assay kits | Nanjing Jiancheng Bioengineering Institute | C013-2-1 | / |
| Bovine Serum Albumin V(BSA) | Servicebio | 9048-46-8 | / |
| DAPI | Servicebio | G1012-10ML | / |
| Cy3 conjugated Goat Anti-Rabbit IgG (H+L) | Servicebio | GB21303 | / |
| FITC conjugated Goat Anti-Mouse IgG (H+L) | Servicebio | GB22301 | / |

**All reagents are from commercial suppliers.**

**Table S3. Source Information for Model Organisms**

| **Identifier** | **Manufacturer** | **Cat#** | **MGI ID** | **RRID** |
| --- | --- | --- | --- | --- |
| Stat3-Flox | Nanjing Model Organisms Co., Ltd. (China) | NM-CKO-200050 | / | / |
| Cdh16-CreERT2 | Nanjing Model Organisms Co., Ltd. (China) | NM-KI-225084 | J:82531 | RRID:IMSR_JAX:012237 |

**Table S4. Cell line origin and verification**

| **Cell line** | **Source (Manufacturer)** | **Cat#** | **Remark** |
| --- | --- | --- | --- |
| HK-2 | Pricella | CL-0109 | Registered in the ATCC repository (Cat# CRL-2190) |
| mTECs | Y. Lan Team  (The Chinese University of Hong Kong) | / | Primary cell immortalization |

For detailed information, please refer to the file ‘STR Identification’.

**Table S5. shRNA and siRNA sequences**

| **Name** | **Sequences** | **Manufacturer** |
| --- | --- | --- |
| Mice Stat3 shRNA | 5'→3':CAGGTATCTTGAGAAGCCAATGGAA | Genepharma |
| Mice Ep300 siRNA | 5'→3':GGAUUAAGUUUGAUAAAUA | Sangon Biotech |
|  | 3'→5':UAUUUAUCAAACUUAAUCC |  |
| Mice Crebbp siRNA | 5'→3':GAUGCUGCGUCCAAACAUA |  |
|  | 3'→5':UAUGUUUGGACGCAGCAUC |  |
| Mice Trim21 siRNA | 5'→3':GGACAUGLUGGGUUCAUAU | Tsingke Biotechnology |
|  | 3'→5':AUAUGAACCCAACAUGUCC |  |
| Human STAT3 siRNA | 5'→3':GCACAATCTACGAAGAATCAA | Hanbio |
|  | 3'→5':UUGAUUCUUCGUAGAUUGUGC |  |

**Table S6. Primer sequences**

| **Primer Name** | **Species** | **F(5'-3')** | **R(3'-5')** |
| --- | --- | --- | --- |
| Stat3 | Mice | CACCTTGGATTGAGAGTCAAGAC | AGGAATCGGCTATATTGCTGGT |
| Lcn2 | Mice | GGACCTCATGGTGGTTACTTTC | TCCCAGGGGCCGTAAACTT |
| Kim1 | Mice | CAGGGAAGCCGCAGAAAA | GAGACACGGAAGGCAACCAC |
| Tnf-α | Mice | CATCTTCTCAAAATTCGAGTGACAA | TGGGAGTAGACAAGGTACAACCC |
| Il-1β | Mice | GCTTCAGGCAGGCAGTAT | ACAAACCGCTTTTCCATCT |
| MCP-1 | Mice | CTTCTGGGCCTGCTGTTCA | CCAGCCTACTCATTGGGATCA |
| β-Actin | Mice | CATTGCTGACAGGATGCAGAA | ATGGTGCTAGGAGCCAGAGC |
| Il-6 | Mice | CTGCAAGAGACTTCCATCCAG | AGTGGTATAGACAGGTCTGTTGG |
| Mrc2 | Mice | TCTCCCGGAACCGACTCTTC | AACTGGTCCCCTAGTGTACGA |
| Ifi44 | Mice | ATGCTCCAACTGACTGCTCG | ACAGCAATGCCTCTTGTCTTT |
| Trim21 | Mice | TGGTGGAGCCTATGAGTATCG | GGCACTCGGGACATGAACTG |
| Cxcl10 | Mice | CCAAGTGCTGCCGTCATTTTC | GGCTCGCAGGGATGATTTCAA |
| Xkr6 | Mice | TCACCATCTCATCCCGAGTTAT | GATCCCTACCACCATGTTGAAG |
| Baiap3 | Mice | CTTCAGTGACCCGTACTGTATGC | TTGACCTCCGTGACCTGGATA |
| Slfn2 | Mice | AAAATGGGCATCAGTGTTGATCT | GGAGAGCACATACAGCTAGAGA |
| Crebbp | Mice | GGCTTCTCCGCGAATGACAA | GTTTGGACGCAGCATCTGGA |
| Ep300 | Mice | TTCAGCCAAGCGGCCTAAA | CGCCACCATTGGTTAGTCCC |
| STAT3 | Human | CAGCAGCTTGACACACGGTA | AAACACCAAAGTGGCATGTGA |
| KIM1 | Human | CTGCAGGGAGCAATAAGGAG | TCCAAAGGCCATCTGAAGAC |
| IL-1β | Human | GAAATGATGGCTTATTACAGTGGC | GTGGTCGGAGATTCGTAGCTG |
| TRIM21 | Human | TCAGCAGCACGCTTGACAAT | GGCCACACTCGATGCTCAC |
| β-ACTIN | Human | TGGCACCCAGCACAATGAA | CTAAGTCATAGTCCGCCTAGAAGCA |

**All primers were designed and synthesized from General Biol.**
