# Supplementary material for: Targeting Stat3 with conditional knockout or PROTAC technology alleviates renal injury by Limiting pyroptosis
Source: eBioMedicine. 2025 May 8;116:105739. doi: 10.1016/j.ebiom.2025.105739 (PMC12136849; doi:10.1016/j.ebiom.2025.105739)
Supplement: Supplementary Figure Legends and Tables [file mmc9.docx]

**Supplementary Figure Legends and Tables**

**Figure S1. Localization of Stat3 and p-Stat3 in the I/R-induced AKI mouse model.** (a) Western blot analysis of Stat3 and p-Stat3 proteins in a CLP-induced AKI mouse model. (b) Western blot analysis of Stat3 and p-Stat3 proteins in an I/R-induced AKI mouse model. (c) IF staining of Stat3 with LTL, Calbindin and DBA in the I/R-induced AKI mouse model. (d) IF staining of p-Stat3 with LTL, Calbindin and DBA in the I/R-induced AKI mouse model. (Scale bar = 50 μm and 10 μm). (e) Western blot analysis of Stat3 and p-Stat3 proteins in mTECs treated with LPS. (f) Western blot analysis of Stat3 and p-Stat3 proteins in mTECs subjected to H/R treatment.

**Figure S2. Validation of Stat3 histone modification and dose selection for MM-102 and C646 treatment.** (a) Western blot analysis of H3K4me1, H3K4me2, H3K4me3, and H3K27ac proteins in the CLP-induced mouse AKI model (n=6). (b) Western blot analysis of H3K4me1, H3K4me2, and H3K4me3 proteins in mTECs induced by LPS and treated with MM-102. (c) Cell viability assay in mTECs treated with different doses of MM-102 (n=6). (d) Optical microscopy observation of cells treated with different doses of MM-102. (e) Western blot analysis of H3K27ac protein in mTECs induced by LPS and treated with C646. (f) Cell viability assay in mTECs treated with different doses of C646. (g) Optical microscopy observation of cells treated with different doses of C646. (h) Knockdown of Crebbp with 3 potential si RNA sequences in mTECs is confirmed by real-time PCR (n=3). (i) Real-time PCR analysis of Stat3 mRNA level in LPS-induced cell model with or without knockdown of Crebbp (n=3). (j) Knockdown of Ep300 with 3 potential si RNA sequences in mTECs is confirmed by real-time PCR (n=3). (k) Real-time PCR analysis of Stat3 mRNA level in LPS-induced cell model with or without knockdown of Ep300 (n=3). (Data are presented as mean ± SEM; ***P < 0.001, ****P < 0.0001, one-way ANOVA. Scale bar = 400 μm)

**Figure S3. Conditional knockout of Stat3 in renal tubular epithelial cells mitigates kidney injury and inflammation induced by CLP and I/R in mice.** (a) Real-time PCR analysis of Stat3 mRNA levels in cKO mice (n=6, t-test). (b) Western blot analysis of Stat3 in cKO mice. (c-d) IF staining of Stat3 with Calbindin and DBA in the CLP-induced AKI mouse model (Scale bar = 50 μm). (e) Real-time PCR analysis of Kim1 mRNA levels in CLP-induced AKI mice with or without conditional knockout of Stat3 (n=6, one-way ANOVA). (f) Quantifying tubular damage in CLP-induced AKI mice with or without conditional knockout of Stat3 (n=6, the Kruskal-Wallis test). (g) Western blot analysis of Kim1 in Stat3^Flox/Flox^ and cKO mice with I/R-induced AKI. (h) Real-time PCR analysis of Lcn2 mRNA levels in I/R-induced AKI mice with or without conditional knockout of Stat3 (n=6, one-way ANOVA). (i) PAS staining of kidney sections of I/R-induced AKI mice with or without conditional knockout of Stat3. (j) Quantifying tubular damage in I/R-induced AKI mice with or without conditional knockout of Stat3 (n=6, the Kruskal-Wallis test).(k) Real-time PCR analysis of Il-1β mRNA levels in I/R-induced AKI mice with or without conditional knockout of Stat3 (n=6, one-way ANOVA). (l) Western blot analysis of Stat3 in aav9-mediated Stat3 overexpression in Stat3^flox/flox^ mice. (m) PAS staining of CLP-induced AKI mice kidney sections after the rescue of Stat3. (n) Quantifying tubular damage in CLP-induced AKI mice after rescue of Stat3 (n=6, the Kruskal-Wallis test). (o) Serum BUN in CLP-induced AKI mice after the rescue of Stat3 (n=6, one-way ANOVA). (p-q) Real-time PCR analysis of Kim1 and Lcn2 mRNA levels in CLP-induced AKI mice after the rescue of Stat3 (n=6, one-way ANOVA). (r) Western blot analysis of p-P65 and P65 in CLP-induced AKI mice after rescue of Stat3. (Data are presented as mean ± SEM; *P < 0.05, **P < 0.01, ***P < 0.001, ****P < 0.0001)

**Figure S4. Verification of Stat3 silencing and overexpression and regulation of Kim1 mRNA levels in mTECs and HK-2.** (a) Knockdown of Stat3 is confirmed by real-time PCR in mTECs (n=3, t-test). (b) Knockdown of Stat3 is confirmed by western blot analysis in mTECs. (c) Real-time PCR analysis of Kim1 mRNA levels in H/R-induced mTECs with or without knockdown of Stat3 (n=3, one-way ANOVA). (d) Overexpression of Stat3 is confirmed by real-time PCR in mTECs (n=3, t-test). (e) Overexpression of Stat3 is confirmed by western blotting in mTECs. (f) Real-time PCR analysis of Kim1 mRNA levels in H/R-induced mTECs with or without overexpression of Stat3 (n=3, one-way ANOVA). (g) Knockdown of STAT3 is confirmed by real-time PCR in HK-2 (n=3, t-test). (h) Real-time PCR analysis of KIM1 mRNA levels in LPS-induced HK-2 with or without knockdown of STAT3 (n=3, one-way ANOVA). (i) Overexpression of STAT3 is confirmed by real-time PCR in HK-2 (n=3, t-test). (j) Real-time PCR analysis of KIM1 mRNA levels in LPS-induced mTECs with or without overexpression of STAT3 (n=3, one-way ANOVA). (Data are presented as mean ± SEM; *P < 0.05, **P < 0.01, ***P < 0.001, ****P < 0.0001)

**Figure S5. Verification of Stat3 regulation of Trim21 protein levels in HK-2.** (a) Western blot analysis of TRIM21 protein in LPS-induced HK-2 with or without knockdown of STAT3. (b) Western blot analysis of TRIM21 protein in LPS-induced HK-2 with or without overexpression of STAT3.

**Figure S6. Protein Levels of Kim1 Mediated by Trim21 Silencing or Overexpression and the Effect of VX-765 on Gsdmd.** (a) Knockdown of Trim21 with 3 potential si RNA sequences in mTECs is confirmed by real-time PCR (n=3, one-way ANOVA). (b) The knockdown of Trim21 in mTECs has been confirmed by western blotting. (c) Western blot analysis of Kim1 protein in LPS-induced cells with or without knockdown of Trim21. (d) Overexpression of Trim21 in mTECs is confirmed by real-time PCR (n=3, t-test). (e) Overexpression of Trim21 in mTECs is confirmed by western blotting. (f) Western blot analysis of Kim1 protein in LPS-induced cells with or without overexpression of Trim21. (g) Cell viability assay of mTECs treated with different concentrations of VX-765 (n=6, one-way ANOVA). (h) Effect of VX-765 on the inhibition of Caspase1 activity. (i) Western blot analysis of Caspase1-p20, Caspase1, Gsdmd-N and Gsdmd proteins in LPS-induced cells with overexpression of Trim21 under treatment of VX-765. (Data are presented as mean ± SEM; *P < 0.05, **P < 0.01, ***P < 0.001, ****P < 0.0001)

**Figure S7. Trim21 Rescue Experiments in mTECs and HK - 2 and the Effects of Overexpressing Stat3 and Silencing Trim21 on Inflammation and Injury in mTECs *in vitro*. (a)** ELISA detection of Il-18 levels in the supernatant of cells after Trim21 rescue *in vitro* (n=3, one-way ANOVA). (b) Overexpression of TRIM21 is confirmed by real-time PCR in HK-2 (n=3, t-test). (c) Western blot analysis of GSDMD-N and GSDMD in LPS-induced HK-2 with or without TRIM21 rescue after STAT3 knockdown. (d-e) Real-time PCR analysis of KIM1 and IL-1β mRNA levels in LPS-induced HK-2 with or without TRIM21 rescue after STAT3 knockdown (n=3, one-way ANOVA). (f) Western blot analysis of p-P65 and P65 in LPS-induced mTECs with or without Trim21 knockdown after Stat3 overexpression. (g-h) Real-time PCR analysis of Kim1 and Mcp-1 mRNA levels in LPS-induced mTEC with or without Trim21 knockdown after Stat3 overexpression (n=3, one-way ANOVA). (Data are presented as mean ± SEM. *P < 0.05, **P < 0.01, ***P < 0.001, ****P < 0.0001)

**Figure S8. *In vivo*, AAV9-mediated silencing of Stat3 mitigates kidney injury, inflammatory response, and pyroptosis induced by CLP and I/R.** (a) Knockdown of Stat3 in mTECs is confirmed by real-time PCR (n=6, t-test). (b) Western blot analysis of Stat3 in mice with or without knockdown of Stat3. (c) Real-time PCR analysis of Il-6 mRNA levels in CLP-induced mice with or without knockdown of Stat3 (n=6, one-way ANOVA). (d) Real-time PCR analysis of Kim1 mRNA levels in CLP-induced mice with or without knockdown of Stat3 (n=6, one-way ANOVA). (e) Western blot analysis of p-65 and p65 in CLP-induced mice with or without knockdown of Stat3. (f) Serum BUN in I/R-induced AKI mice with or without knockdown of Stat3 (n=6, one-way ANOVA). (g-h) Real-time PCR analysis of Trim21 and Tnf-α mRNA levels in I/R-induced mice with or without knockdown of Stat3 (n=6, one-way ANOVA). (i) ELISA for the determination of Il-18 levels in the serum from I/R-induced mice with or without knockdown of Stat3 (n=6, one-way ANOVA). (Data are presented as mean ± SEM. **P < 0.01, ***P < 0.001, ****P < 0.0001)

**Figure S9. Synthesis of degrader E034.** Reagents and conditions: (a) 1) Pd(PPh3)2Cl2, CuI, Et3N, DMF, 80℃; 2) TFA/DCM, 0℃; (b) 1) HATU, DIEA, DMF, 20℃; 2) TFA/DCM, 0℃; (c) HATU, DIEA, DMF, 20℃; (d) TMSI, BSTFA, DCM, 0℃.

**Figure S10. The structural characterization data of degrader E034.** (a) HRMS of degrader E034. (b) ^1^H NMR spectrum of degrader E034. (c) ^19^F NMR spectrum of degrader E034. (d) ^31^P NMR spectrum of degrader E034. (e) HPLC spectrum of degrader E034.

**Figure S11. *In vitro* dose selection and effects of E034 on LPS- and H/R-induced cell injury, inflammation, and pyroptosis.** (a) Cell viability assay of mTECs treated with different concentrations of E034 (n=6, one-way ANOVA). (b) Effect of E034 on the degradation rate of Stat3. (c) Western blot analysis of Stat1, Stat3, Stat5 and Stat6 LPS-induced cells treated with 0.1, 0.2 and 0.4 μM E034. (d) IF staining of Kim1 in LPS-induced cells treated with 0.1, 0.2, and 0.4 μM E034 (Scale bar = 50 μm). (e) ELISA for determining Il-18 levels in the supernatant of LPS-induced cells treated with 0.1, 0.2 and 0.4 μM E034 (n=3, one-way ANOVA). (f) Western blot analysis of Kim1, p-65, and p65 H/R-induced cells treated with 0.4 μM E034. (g-h) Real-time PCR analysis of Kim1, Mcp-1 and Tnf-α mRNA levels H/R-induced cells treated with 0.4 μM E034 (n=3, one-way ANOVA). (i-j) ELISA for determining Il-1β and Il-18 levels in the supernatant of H/R-induced cells treated with 0.4 μM E034 (n=3, one-way ANOVA). (Data are presented as mean ± SEM. **P < 0.01, ***P < 0.001, ****P < 0.0001)

**Figure S12. *In vivo* dose selection of E034 and preventive treatment strategy.** (a) Serum BUN in mice treated with different doses of E034 (n=3, one-way ANOVA). (b) Western blot analysis of Stat3 in mice treated with different doses of E034. (c) Schematic diagram of the preventive treatment strategy. (d) Serum BUN in CLP-induced AKI mice after pre-treatment with 0.05, 0.1, and 0.2 mg/kg E034 (n=6, one-way ANOVA). (e) Quantification of tubular damage (n=6, the Kruskal-Wallis test). (f) Real-time PCR analysis of Kim1 mRNA levels in CLP-induced AKI mice after pre-treatment with 0.05, 0.1, and 0.2 mg/kg E034 (n=6, one-way ANOVA). (g) IHC staining of F4/80+ macrophage infiltration in CLP-induced AKI mice after pre-treatment with 0.05, 0.1, 0.2 mg/kg E034. (h) ELISA was used to determine Il-18 levels in the serum from CLP-induced AKI mice after pre-treatment with 0.05, 0.1, and 0.2 mg/kg E034 model (n=6, one-way ANOVA). (Scale bar = 50 μm; Data are presented as mean ± SEM. *P < 0.05, **P < 0.01, ****P < 0.0001)

**Figure S13. Treatment strategy with E034.** (a) Schematic diagram of the treatment strategy. (b) Serum BUN in CLP-induced AKI mice treated with 0.2 mg/kg E034. (c) PAS staining of kidney sections of CLP-induced AKI mice treated with 0.2 mg/kg E034 (Scale bar = 50 μm). (d) Quantification of tubular damage. (e) ELISA for the determination of Il-1β levels in the serum from CLP-induced AKI mice treated with 0.2 mg/kg E034 (n=6; Data were analyzed using one-way ANOVA and are presented as mean ± SEM. **P < 0.01, ****P < 0.0001.)

**Figure S14. E034 exhibits minimal toxic side effects on other organs.** (a) Serum AST and ALT levels after treatment with 0.2 mg/kg E034. (b) H&E staining of the heart, liver, spleen and lungs after treatment with 0.2 mg/kg E034. Scale bar = 200 μm. (n=6; Data are presented as mean ± SEM; one-way ANOVA)

**Figure S15. Graphic summary**

**Figures S16-23** are included in the **Reagent Validation file.**

**Tables S1-6** are included in the **Reagent Validation file.**

**Table S7.** **The clinical characteristics of the patients with AKI in this study**

| **Group** | **Sex** | **Age (years)** | **Race** | **Diagnosis** | **Serum creatinine (umol/l)** | **Serum urea nitrogen (mmol/l)** |
| --- | --- | --- | --- | --- | --- | --- |
| Para-carcinoma | male | 45 | East Asian | Renal carcinoma paracancerous tissue | - | - |
| Para-carcinoma | male | 67 | East Asian | Renal carcinoma paracancerous tissue | - | - |
| Para-carcinoma | female | 50 | East Asian | Renal carcinoma paracancerous tissue | - | - |
| Para-carcinoma | female | 60 | East Asian | Renal carcinoma paracancerous tissue | - | - |
| AKI | male | 44 | East Asian | acute serious tubular damage | 702 | 17.5 |
| AKI | male | 74 | East Asian | acute serious tubular damage | 424 | 15.46 |
| AKI | male | 55 | East Asian | septic acute kidney injury | 973 | 23.32 |
| AKI | female | 62 | East Asian | acute serious tubular damage | 658 | 19.29 |
